# Ethnomedicinal knowledge of plants used in alternative medicine as uterotonics in Lubumbashi, Haut-Katanga, Democratic Republic of Congo

**DOI:** 10.1371/journal.pone.0355373

**Published:** 2026-08-14

**Authors:** Bashige Chiribagula Valentin, Okusa Ndjolo Philippe, Cedrick Shakalenga Mutombo, Manya Mboni Henry, Numbi wa Ilunga Evodie, Bakari Amuri Salvius, Jean Claude Nkwanga, Muyumba Singoy Sarah, Mwamba Maseho Faustin, Biayi Benaja Martin, Sangwa Kamulete Gregoire, Mbayo Kalubandika Glaubert, Muhona Melman, Lumbu Simbi Jean Baptiste

**Affiliations:** 1 Department of Pharmacology, Laboratory of Therapeutic Chemistry and Analysis of Natural Substances, Faculty of Pharmaceutical Sciences (UNILU), Commune Kampemba, Lubumbashi, Democratic Republic of Congo; 2 Department of Pharmacology, Laboratory of Pharmacognosy, Faculty of Pharmaceutical Sciences, University of Lubumbashi (UNILU), Commune Kampemba, Lubumbashi, Democratic Republic of Congo; 3 Department of Chemistry, Faculty of Sciences, University of Lubumbashi (UNILU), Commune of Lubumbashi, Democratic Republic of Congo; Guru Ghasidas Vishwavidyalaya, INDIA

## Abstract

**Background:**

Traditional medicine constitutes a principal component of primary healthcare in Democratic Republic of Congo (DRC), particularly in Lubumbashi. However, ethnobotanical knowledge related to its use remains incompletely documented and underutilized. The present study aimed to systematically document medicinal plants used as uterotonic among residents of Lubumbashi.

**Methods:**

Ethnobotanical data were collected through structured interviews with 207 informants (housewives, 𝑛 = 103; herbalists, 𝑛 = 26; traditional healers, 𝑛 = 78; sex ratio M/F = 0.5; mean age 35.6 ± 4.3 years; mean professional experience 13 ± 4.2 years).

**Results:**

Informants cited 98 medicinal plant species, predominantly trees (38 species), distributed across 43 botanical families, with Fabaceae being the most represented family (16 species). Malaria was the most frequently reported non‑uterotonic indication (46 species). A total of 122 traditional oxytocic formulations were recorded; most involved leaves (43 recipes) and were principally administered as cataplasm (44 recipes). To our knowledge, 34 species were reported for the first time with oxytocic uses, of which eight are documented for the first time as medicinal plants in DRC. Among taxa, *Hylodesmum repandum* (relative citation frequency, RCF = 0.20) and *Crassocephalum montuosum* (RCF = 0.19) exhibited the highest citation frequencies, while *Securidaca longepedunculata* (use value, UV = 0.27) and *Hibiscus sabdariffa* (UV = 0.25) showed the greatest overall use values.

**Conclusion:**

These findings provide a baseline inventory for pharmacological, phytochemical, and toxicological studies, and support prioritization of bioassay-guided validation and conservation strategies for culturally important species.

## 1. Introduction

Childbirth remains a major public health challenge in Sub-Saharan Africa and other low- and middle-income countries, including the Democratic Republic of Congo (DRC). Despite advances in modern medicine, maternal and neonatal mortality rates remain high, with a reported rate of 444 maternal deaths per 100,000 births over the past decade [1–3]. The main causes include hemorrhage, hypertensive disorders, infections, unsafe abortions and obstructed labor. These complications which are common due to socioeconomic inequalities, poverty, limited access to healthcare facilities and a shortage of qualified staff, particularly in rural areas [4,5], could be prevented with rapid access to quality care [5].

Uterotonics are substances that stimulate myometrial receptors, thereby increasing uterine tone and contractions [6]. These include oxytocin, Carbetocin, misoprostol, ergot and prostaglandin derivatives [7]. Concurrently, pregnant women also utilize natural substances to facilitate childbirth [8,9]reflecting their reliance on traditional medicine due to the constraints imposed by modern medical options.

In addition, conventional medicine has advanced obstetric care through technologies such as prenatal imaging, electronic fetal monitoring, and artificial intelligence algorithms that support conventional uterotonics [10,11]. These advances are inaccessible to a significant proportion of the African population, particularly in DRC, where human and material resources are severely limited [12].

In DRC, traditional medicine plays a central role in childbirth management using plants with uterotonic properties. A review of the literature from 1980 to 2010 identified 280 African oxytocic plants [13], while another reported 16 plants [14] of which the following demonstrated experimental uterotonic activity: *Heimia salicifolia* Link, Lythraceae [15], the roots of *Azanza garckeana* (F. Hoffm.) Exell & Hillc., a synonym for *Thespesia garckeana* F. Hoffm, Malvaceae [16], as welle and the stem bark of *Viburnum opulus* L.,Viburnaceae [17].

In the city of Lubumbashi, to the best of our knowledge, there has been no recorded study systematically documenting uterotonic plants. However, an ethnobotanical investigation conducted by Nama Mwengu (2021) within the Ruashi commune of Lubumbashi documented six plant species traditionally employed for their uterine effects: *Phyllanthus muellerianus* (Kuntze) Exell, *Hibiscus hiernianus* Exell & Mendonça (syn. *Hibiscus gilletii* subsp. hiernianus), *Acacia macrocentra* Walp (Syn. *Mimosa arenosa var. arenosa*), *Pseudolachnostylis maprouneifolia* Pax, *Strychnos innocua* Delile, and *Annona senegalensis* Pers [18]. Complementarily, a subsequent survey undertaken in Kipushi in 2017, a locality proximal to Lubumbashi, identified eleven plants reputed to facilitate childbirth. Of these, six species: *Strychnos cocculoides* Baker, *Uapaca kirkiana* Müll.Arg, *Phyllanthus muellerianus*, *Ficus capensis* Thunb (Syn. *Ficus sur* Forssk), *Byrsocarpus orientalis* (Baill.) Baill (Syn. *Rourea orientalis* Baill), and *Aframomum alboviolaceum* (Ridl.) K.Schum, were specifically cited for their putative uterotonic properties [19].

This study aimed to identify medicinal plants and recipes with uterotonic effects, used in Lubumbashi’s traditional medicine to facilitate childbirth.

## 2. Materials and methods

### 2.1. Study area

**Fig 1:**
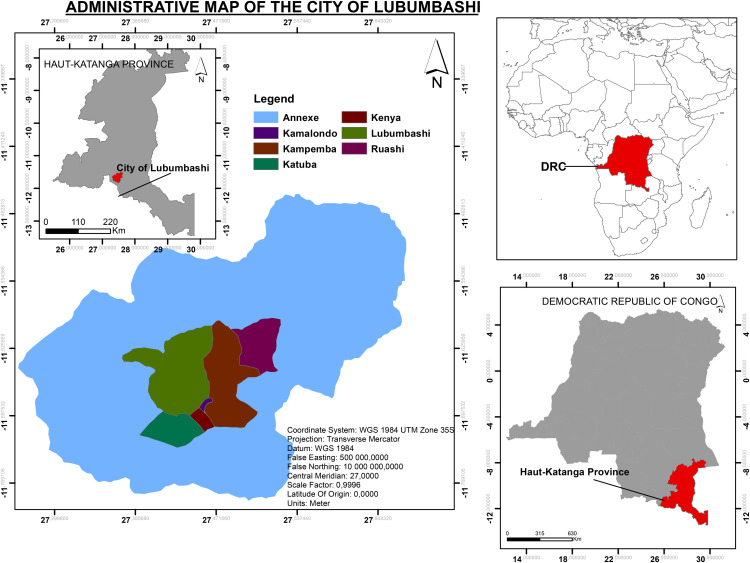
Lubumbashi, Haut-Katanga province in Democratic Republic of Congo.

The study was conducted in the seven communes of Lubumbashi: Annexe, Kampemba, Katuba, Kamalondo, Kenya, Lubumbashi, and Ruashi, in the Haut-Katanga province in the Democratic Republic of Congo (Fig 1).

Lubumbashi is located between 11°26′ and 11°55′ South latitude and 27°15′ and 27°40′ East longitude, at an altitude of approximately 1,230 m above sea level. The region has a tropical climate characterized by a rainy season (November–April) and a dry season (May–October), with a mean annual temperature of 22.4 °C and average annual rainfall of approximately 512 mm. The dominant vegetation type is Miombo woodland, a drought-adapted ecosystem that constitutes an important source of medicinal plant diversity for local communities [20,21].

### 2.2. Ethnobotanical data collection

Between November 2024 and October 2025, a cross-sectional descriptive ethnobotanical survey was conducted among 207 informants comprising, 103 women who had experienced difficult labor and used medicinal plants to facilitate delivery, 26 herbalists, and 78 traditional healers, through direct, open-ended interviews guided by a Semi-structured questionnaire (S1 Table). This survey was pre-tested with 10 women who had experienced difficult childbirth and 5 traditional healers, which helped improve the informants’ understanding and compliance.

To determine the minimum sample size to interview among women who have previously experienced difficulties in childbirth, we used probabilistic sampling, given that their number is unknown but estimated to be substantial. This sample size was calculated using the following Cochran formula:


$$\begin{array}{c} \text{n }=\;\frac{z^{\text{2}}*p*(\text{1}-p)}{e^{\text{2}}} \end{array}$$ (1)

(z = 1.96 for 95% confidence, p = 0.50, e = 0.05), yielding 196 informants. These women were randomly selected from different households in Lubumbashi. Of the 196 women consulted, only 103 (52.5%) reported using plants to accelerate uterine contractions.

The herbalists included in this study were recruited from six major traditional medicine markets in Lubumbashi (Mzee, Kenya, Zambia, Kamalondo, Kalala Banza, and Moise), which serve as dynamic hubs of ethnobotanical knowledge and living plant traditions. We approached all herbalists encountered in these markets who consented to participate. In total, thirty-six practitioners were interviewed. Through direct interviews, they shared the therapeutic recipes they employ, revealing that twenty-six (72%) reported at least one specific uterotonic herbal formulation.

Traditional healers included in this study were initially selected from a pre-established list of 50 practitioners compiled during a previous survey [20]. This process resulted in the identification of 47 traditional healers, of whom only 45 reported using uterotonic medicinal plants. To increase the sample size, snowball sampling was subsequently employed, resulting in the inclusion of 33 additional healers, one of whom had already appeared on the initial list.

### 2.3. Data processing and analysis

Plant specimens were collected in the presence of informants, photographed, pressed, and dried following standard herbarium procedures. Taxonomic identification was performed by qualified botanists at the Kipopo Herbarium (Lubumbashi, DRC). Voucher specimens were deposited under assigned reference numbers. Scientific names were verified and updated using Plants of the World Online (POWO: https://powo.science.kew.org/) and World Flora Online databases (WFO: http://www.worldfloraonline).

To determine the status of each inventoried taxon in relation to the contemporary scientific record, the accepted botanical name of each species was systematically queried in principle bibliographic databases (Google Scholar, PubMed, Scopus). For every species, we documented the presence or absence of previous ethnobotanical reports of use as a uterotonic; experimentally verified uterotonic activity; documentation of medicinal use within the Democratic Republic of Congo; documentation of medicinal use specifically from Lubumbashi; and documentation of medicinal use from regions outside the DRC. These categorical assessments were employed to characterize the current state of knowledge for each plant.

Given the current controversy surrounding ethnobotanical indexes [22]We have used only two ethnobotanical indices to assess the significance of the reported species: the Use Value (UV) and the Relative Citation Frequency (RCF). However, apart from these two ethnobotanical indices, we have expressed the results as percentages (%).

The formula determines the use value (UV):


$$\begin{array}{c} \text{UV }=\;\frac{\text{Number of uses mentioned by an informant for a plant }}{\text{Number of informants who cited the plant}} \end{array}$$ (2)

The Relative citation frequency on the plant (organ) (RCF) was calculated by the formula:


$$\begin{array}{c} \text{RCF }=\frac{\text{Number of informant who cited the plant }}{\text{Number of informants who took part in the survey}} \end{array}$$ (3)

In this study, the UV index is used to assess the medicinal importance of a plant in the study area; the RCF index measures a plant’s popularity among people who use it as uterotonic. This also implies a consensus among informants regarding its use to stimulate uterine contractions during childbirth.

### 2.4. Ethics considerations

Ethical approval for this study was granted by the Research Medical Ethics Commission of the University of Lubumbashi (UNILU/CEM/035/2024), and we obtained informed verbal consent from all 207 participants after clearly explaining the study’s purpose and procedures. Any individual who declined to consent or had no prior experience using medicinal plants to aid childbirth was respectfully excluded. We reassured each informant that their insights would be used solely for academic research, that personal identifiers would be removed, and that no personal information would be shared with third parties.

## 3. Results

### 3.1. Profile of inventoried medicinal plants

A total of 98 plant species were identified as being used as uterotonic agents in traditional medicine in Lubumbashi, following ethnobotanical surveys of medicinal plants of traditional practitioners, herbalists, and housewives. Of these species, 61 were reported by traditional practitioners (35 of which were also mentioned by the other categories of informants), 35 by herbalists (24 of which were mentioned by the other categories of informants), and 28 by housewives (19 of which were mentioned by the other categories of informants). The general characteristics of these plants and their status as prior knowledge (bibliographic) in relation to uterotonic use are shown in Table 1.

**Table 1:** General information of plants used as uterotonic agents in Lubumbashi.

Species (Family, HC)	Appellation (Ethnicity)^GT, MT^	N^o^ Recipe (n = 107) ^UP,P^	N_C_	RCF (n = 207)	N_u_	UV (n = 60)	Non uterotonic indication	Ref.
*Abrus precatorius* L (Fabaceae, KIP000000489)	Buhiti (Lamba) ^h,p^	**R1** ^w,ε^	9	0.04	9	0.15	Bronchitis, jaundice, hepatic diseases, contraception, tumors, sexual dysfunction, malaria, and snake bites	†: [23,24] ‡: [24,25] ¥: [26]
*Adenia gummifera* (Harv.) Harms (Passifloraceae, KIP000000453)	Kimboyi (Lala) ^k,l^	**R2** ^α,μ^	9	0.04	8	0.13	Infertility, sexually transmitted infections, gastrointestinal diseases, leprosy, respiratory infections, malaria and menstrual problems	†: NR ‡: NR ¥: [26]
*Aerva javanica* (Burm.f.) Juss. Ex Schult. (Amaranthaceae, KIP000000601)	Kapoka (Bemba) ^c,q^	**R3** ^x,δ^	8	0.04	9	0.15	Cancer, wound, helminth, atherosclerosis, diabetes, hyperlipidemia, fungal infection, and hepatic diseases	†: NR ‡: [27] ¥: [28] ₮: [28,29]
*Aframomum alboviolaceum* (Ridl.) K.Schum. (Zingiberaceae KIP000001433)	Ntundulu (Kikongo) ^f,o^	**R4** ^α,δ^	9	0.04	14	0.23	Malaria, helminth, fever, Dysmenorrhea, snake bites, headache, cough, gastritis, amoebic dysentery, hemorrhoids, myoma, and pruritic.	†: NR ‡: NR ₸: [30]
		**R5** ^z,ε^						
*Afrothismia mhoroana* Cheek [Syn *Rauvolfia caffra* L] (Burmanniaceae, KIP000000602)	Mutalala (Bemba)^b,o^	**R6** ^α,δ^	12	0.06	4	0.07	Fever, headaches, and gastrointestinal diseases	†: NR ‡: NR ₮: [31]
*Ageratum conyzoides* L. (Asteraceae, KIP000111014)	Uratulu (Kikongo) ^d,l^	**R7** ^x,δ^	28	0.14	4	0.07	Wounds, skin infections, and respiratory ailments	†: NR ‡: [32] ₸: [33]
*Albizia adianthifolia* (Schumach.) W.Wight (Fabaceae, KIP000001031)	Kapeta nzovu (Bemba) ^f,r^	**R8** ^β,ψ^	29	0.14	11	0.18	Diabetes, eye problems, gastrointestinal diseases, hemorrhoids, headache, neurodegenerative disorders, respiratory ailments, wounds, skin diseases, sexually transmitted infections	†: NR ‡: NR ₸: [33]
*Albizia lebbeck* (L.) Benth (Fabaceae, KIP000111016)	Siriza (Lamba) ^d,r^	**R9** ^w, ε^	11	0.05	11	0.18	Fever, diarrhea, dysentery, asthma, cold, cough, conjunctivitis, arthritis, dental problems and skin disease	†: NR ‡: NR ¥: [34]
		**R10** ^w, λ^						
*Alchornea floribunda* Müll.Arg (Euphorbiaceae, KIP000000603)	Lubumbidi (Kiyombe) ^f,p^	**R11** ^γ,μ^	17	0.08	3	0.05	Inflammatory diseases, fungal ailments, and diarrhea	†: NR ‡: NR ¥: [35]
*Allium sativum* L (Amaryllidaceae)	Sabolo (Sanga) ^d,m^	**R12** ^u,δ^	12	0.06	9	0.15	Fungal ailments, cancer, thrombosis, cardiovascular diseases, diabetes, viral diseases and parasitic diseases	†: [36] ‡: [37] ₸: [38]
*Aloe chabaudii* Schönland (Asphodelaceae, KIP000000604)	Kilambwe ne Byana (Bemba) ^f,n^	**R13** ^γ, δ^	17	0.08	8	0.13	Skin diseases, Inflammatory diseases, respiratory ailments, urinary diseases, Poisoning, snake bites, cardiovascular diseases	†: NR ‡: NR ₮: [39,40]
*Aloe vera* (L.) Burm.f (Asphodelaceae, KIP000000605)	Cizimyamuliro (Shi) ^d,n^	**R14** ^γ,δ^	15	0.07	11	0.18	Skin diseases, Inflammatory diseases, respiratory ailments, urinary diseases, Poisoning, snake bites, cardiovascular diseases, wounds, diabetes, sexually transmitted infections	†: [41] ‡: [42] ₸: [43]
*Anchusa ovata* Lehm (Boraginaceae, KIP000000606)	Kandasole (Sanga) ^j,l^	**R15** ^α,μ^	12	0.06	6	0.10	Ulcer, cough, fever, wounds, skin diseases	†: NR ‡: NR ₮: [44]
		**R16** ^α, δ^						
*Annona senegalensis* Pers (Annonaceae, KIP452120003)	Mulolo (Bemba) ^i,r^	**R17** ^y, ε^	7	0.03	7	0.12	Prostate cancers, respiratory diseases, poisoning, rheumatic pains, infertility care and syphilis	†: NR ‡: [32] ₸: [43]
*Antidesma venosum* E.Mey. ex Tul (Phyllanthaceae, KIP293612638)	Kifubia (Luba-Kat) ^h,p^	**R18** ^β, ε^	35	0.17	7	0.12	Typhoid, gastrointestinal diseases, diabetes, pneumonia, wounds, and sexually transmitted infections	†: NR ‡: NR ₸: [33]
*Asparagus africanus* Lam. (Asparagaceae, KIP000111026)	Atangwali (Hemba) ^h,p^	**R19** ^γ, ε^	5	0.02	17	0.28	Malaria, helminth, fever, Dysmenorrhea, headache, cough, gastritis, amoebic dysentery, stomach pain, ulcers, hemorrhoids, myoma, syphilis, gonorrhea, and pruritis.	†: [45] ‡: [46,47] ₮: [48]
*Azadirachta indica* A. Juss. (Meliaceae, KIP000000607)	Nfwama (Sabwa) ^d,r^	**R20** ^γ, ε^	4	0.02	13	0.22	Fever, malaria, wounds, headache, ulcers, respiratory diseases, cancer, diabetes, leprosy, dengue fever, chickenpox, and skin diseases	†: [41] ‡: [27] ₸: [43]
		**R21** ^z, λ^						
*Baphiopsis parviflora* Benth. ex-Baker (Fabaceae, KIP000000608)	Waase (Tabwa) ^a,r^	**R22** ^β, ε^	5	0.02	9	0.15	Wounds, coughs, hypertension, erectile dysfunction, skin infections, urinary tract infections, respiratory ailments, and gastro-intestinal diseases.	†: NR ‡: NR ¥: [49,50]
*Bidens pilosa* L (Asteraceae, KIP000000609)	Mbamba ngoma (Luba-Kat) ^d,l^	**R23** ^γ, θ^	5	0.02	6	0.10	Intoxication, hypertension, dysentery, hepatic diseases, ulcers	†: [41,51] ‡: [52] ₮: [30]
*Bobgunnia madagascariensis* (Desv.) J.H.Kirkbr. & Wiersema (Fabaceae, KIP000000610)	Munienze (Luba-Kat) ^f,r^	**R24** ^β, ε^	6	0.03	9	0.15	Bilharzia, leprosy, cataract, dysentery, headache, cough, diarrhea, and sexually transmitted infections	†: NR ‡: NR ₮: [30]
*Boerhavia diffusa* L (Nyctaginaceae, KIP000000611)	Alini (Bemba) ^h,l^	**R25** ^γ, δ^	5	0.02	8	0.13	Cough, hypertension, malaria, fever, diabetes, wound, gastro-intestinal diseases	†: NR ‡: NR ¥: [53]
*Bombax ceiba* L. (Malvaceae, KIP000000612)	Mulul ilav (Rund) ^d,r^	**R26** ^y, ε^	5	0.02	6	0.10	Cholera, coughs, urinary tract infections, dysentery, and gastro-intestinal diseases	†: NR ‡: NR ₮: [54]
*Bothriocline longipes* (Oliv. & Hiern) N.E.Br. (Asteraceae, KIP000000613)	Lwibaye (Shi) ^f,q^	**R27** ^γ,θ^	6	0.03	14	0.23	Fever, malaria, wound, headache, ulcers, respiratory diseases, cancer, diabetes, leprosy, dengue fever, chickenpox, and skin diseases, stomach	†: [55] ‡: [25] ₮: [56]
		**R28** ^α,δ^						
*Cajanus cajan* (L.) Huth (Fabaceae, KIP000000614)	Golyolyo (Luba-Kat) ^d,n^	**R29** ^z, ε^	7	0.03	11	0.18	Diabetes, malaria, ulcers, gastro-intestinal diseases, hemorrhoids, headache, respiratory ailments, wounds, skin diseases, sexually transmitted infections	†: [57] ‡: [58] ₮: [38]
*Capsicum annuum* L. (Solanaceae, KIP000000615)	Pilipili (Swahili) ^d,l^	**R30** ^z, δ^	12	0.06	6	0.10	Cancers, wounds, fever, malaria, stomach	†: NR ‡: [32] ₮: [59]
*Cassia sieberiana* DC (Fabaceae, KIP000000616)	Kafunga nanshi (Bemba) ^f,r^	**R31** y, ε	9	0.04	8	0.13	Fever, indigestion, constipation, Drepanocytosis, diabetes, malaria, and skin diseases	†: NR ‡: NR ¥: [26]
*Cissampelos mucronata* A.Rich. (Menispermaceae, KIP000000617)	Mukoko (Luba-Kat) ^h,n^	**R32** ^γ, δ^	7	0.03	6	0.10	Diarrhea, dysentery, sexually transmitted infections, infertility, fever	†: [60] ‡: [58] ₮: [61,62]
*Cissus schmitzii* Dewit (Vitaceae, KIP000000618)	Lenda (Luba-Kat) ^a,p^	**R33** ^α, ε^	9	0.04	7	0.12	Wounds, ulcers, fever, respiratory ailments, sexual dysfunction, diabetes	†: NR ‡: NR ₸: [63]
*Clausena anisata* (Willd.) Hook.f. ex Benth (Rutaceae, KIP000000619)	Umusanga (Bemba) ^f,r^	**R34** ^γ, ε^	10	0.05	5	0.08	Hypertension, respiratory ailments, fever, stomach	†: NR ‡: [64] ₮: [64]
*Clematis scabiosifolia* DC [Syn *Clematis villosa* subsp. Villosa] (Ranunculaceae, KIP000000620)	Karhula (Shi) ^a,n^	**R35** ^γ, θ^	11	0.05	7	0.12	Cancer, Pain, Inflammatory diseases, headaches, skin diseases, sexually transmitted infections	†: NR ‡: [65] ₸: [65]
*Cochlospermum planchonii* Hook.f. ex Planch (Bixaceae, KIP000000621)	Sowaga (Tabwa) ^f,q^	**R36** ^γ, θ^	15	0.07	6	0.10	Malaria, diarrhea, hepatic diseases, Inflammatory diseases, stomach	†: NR ‡: NR ₮: [66,67]
*Combretum molle* R.Br. ex G.Don (Combretaceae, KIP000000622)	Mulema (Bemba) ^f,p^	**R37** ^γ, μ^	14	0.07	8	0.13	Drepanocytosis, diarrhea, helminth, wound, ulcers Poisoning, headaches	†: NR ‡: NR ₸: [38]
*Commelina africana* L (Commelinaceae, KIP000000623)	Idangabani (Bemba) ^i,n^	**R38** ^y, ε^	13	0.06	6	0.10	Drepanocytosis, urinary tract infections, diarrhea, hemorrhoids, stomach	†: [41] ‡: [68] ¥: [69]
*Crassocephalum montuosum* (S.Moore) Milne-Redh. (Asteraceae, KIP437629969)	Cifula (Shi) ^i,l^	**R39** ^γ, δ^	39	0.19	14	0.23	Fever, malaria, wound, headache, ulcers, respiratory diseases, cancer, diabetes, leprosy, rheumatism, dengue fever, chickenpox, and skin diseases	†: NR ‡: NR ₸: [70]
		**R40** ^y,δ^						
*Crassocephalum vitellinum* (Benth.) S.Moore (Asteraceae, KIP000000624)	Shungululu (Shi) ^f,l^	**R41** ^γ, δ^	47	0.23	7	0.12	Fever, malaria, sexually transmitted infections, wounds, gastro-intestinal diseases, stomach	†: NR ‡: [25] ¥: [65]
*Cucurbita pepo* L (Cucurbitaceae, KIP000000625)	Liboga (Swahili) ^d,l^	**R42** ^z, δ^	11	0.05	7	0.12	hepatic diseases, diabetes, cancer, ulcer, wound, stomach	†: NR ‡: [71] ¥: [72]
*Cyanthillium cinereum* (L.) H.Rob. [syn *Vernonia cinerea* (L.) Less] (Asteraceae, KIP000000626)	Musubgu bululu (Luba-kas) ^g,n^	**R43** ^γ, δ^	13	0.06	5	0.08	Urinary tract infections, diarrhea, cough, respiratory diseases, stomach	†: NR ‡: NR ₸: [38]
*Cymbopogon citratus* (DC.) Stapf (Poaceae, KIP000000627)	Masela mputu (Luba-Kas) ^d,n^	**R44** ^α, ε^	11	0.05	8	0.13	Fever, stomach, headache, malaria, inflammatory diseases, cough, Amoebae	†: [73] ‡: [32] ₸: [20,33]
*Cynodon dactylon* (L.) Pers. (Poaceae, KIP000000628)	Lukaka (Sanga) ^e,l^	**R45** ^x, δ^	12	0.06	6	0.10	Inflammatory diseases, fungal ailments, hypertension and diarrhea, wound	†: NR ‡: [74] ₸: [75]
*Dalbergia boehmii* Taub (Fabaceae, KIP000000629)	Katambo (Zela) ^f,q^	**R46** ^β, δ^	12	0.06	7	0.12	Diabetes, urinary tract infections, skin diseases, pain, Diarrhea, fever	†: NR ‡: [74] ₸: [38]
*Diplorhynchus condylocarpon* (Müll.Arg.) Pichon [Syn *Diplorhynchus mossambicensis* Benth] (Apocynaceae, KIP361753009)	Mwenge (Sanga) ^f,p^	**R47** ^y, δ^	14	0.07	6	0.10	Headache, snake bites, hepatic diseases, respiratory diseases, gastrointestinal diseases	†: NR ‡: [76] ₸: [38,63]
*Ekebergia capensis* Sparrm (Meliaceae, KIP000000630)	Nyamaru (Bemba) ^h,r^	**R48** ^z, δ^	13	0.06	9	0.15	Fever, malaria, stomach, skin diseases, wounds, cough, headache	†: [77] ‡: [14] ₮: [78]
*Elaeis guineensis* Jacq (Arecaceae, KIP000000631)	Ekaci (Bembe) ^f,r^	**R49** ^β, δ^	9	0.04	6	0.10	headaches, pains, rheumatism, cardiovascular diseases, wounds	: NR ‡: [79] ₸: [38]
*Eleusine indica* (L.) Gaertn (Poaceae, KIP000000632)	Palasi (Zela) ^i,l^	**R50** ^γ, θ^	4	0.02	6	0.10	asthma, diarrhea, stomach, dysentery, respiratory diseases	†: NR ‡: [52] ₸: [38]
*Erythrina abyssinica* Lam (Fabaceae, KIP000000633)	Kisungwa (Bemba) ^f,r^	**R51** ^y, μ^	5	0.02	12	0.20	Tuberculosis, malaria, HIV/AIDS, diarrhea, cancer, meningitis, inflammatory diseases, urinary tract infections, wounds, diabetes, and skin diseases.	†: NR ‡: [58] ₸: [38]
*Euphorbia hirta* L. (Euphorbiaceae, KIP000000634)	Butonvitonvi (Bemba) ^f,l^	**R52** ^x, ε^	13	0.06	5	0.08	Cancer, Asthma, wounds, gastrointestinal diseases	†: NR ‡: [32,58] ₸: [33,38]
*Ficus capensis* Thunb [*Ficus sur* Forssk] (Moraceae, KIP539378804)	Mukiluakilua (Bemba) ^h,r^	**R53** ^y, δ^	37	0.18	8	0.13	Drepanocytosis, diarrhea, fever, cough, ulcers, skin diseases, stomach	†: NR ‡: [32] ₸: [38]
*Ficus stuhlmannii* Warb (Moraceae, KIP000111046)	Mupulampaka (Bemba) ^f,r^	**R54** ^y, δ^	39	0.19	6	0.10	Drepanocytosis, wounds, hemorrhoids, diabetes, hepatic diseases	†: NR ‡: NR ₸: [33,63]
*Flueggea virosa* (Roxb. ex Willd.) Royle (Phyllanthaceae, KIP000000635)	Kasansubwanga (Bemba) ^f,p^	**R55** ^γ, ε^	12	0.06	5	0.08	Fever, gastrointestinal diseases, skin diseases, stomach	†: NR ‡: NR ₸: [38]
*Grona adscendens* (Sw.) H.Ohashi & K.Ohashi (Syn *Desmodium adscendens* (Sw.) DC] (Fabaceae, KIP000000636)	Busundu (Shi) ^f,n^	**R56** ^γ, δ^	17	0.08	5	0.08	asthma, diarrhea, stomach, dysentery, respiratory diseases	†: NR ‡: NR ₮: [80,81]
*Gymnanthemum amygdalinum* (Delile) Sch.Bip. [Syn *Vernonia amygdalina* Delile] (Asteraceae, KIP312286606)	Mubirizi (Shi) ^f,p^	**R57** ^β, ε^	20	0.10	9	0.15	fever, malaria, diarrhea, cough, dysentery, headache, stomach pains, and hepatic diseases	†: [41,68,82] ‡: [58] ₸: [38]
*Harungana madagascariensis* Lam. Ex Poir (Hypericaceae, KIP000000637)	Kafifi (Bemba) ^f,r^	**R58** ^y,ε^	8	0.04	6	0.10	Drepanocytosis, malaria, gastrointestinal diseases, diabetes, hepatic diseases	†: NR ‡: NR ₸: [38]
*Hexalobus monopetalus* (A.Rich.) Engl. & Diels (Annonaceae, KIP000000638)	Nkoyo (Luba-kat) ^f,r^	**R59** ^α,μ^	19	0.09	5	0.08	Fever, headache, gastrointestinal diseases, stomach	†: NR ‡: NR ₸: [63]
*Hibiscus sabdariffa* L (Malvaceae, KIP000111148)	Mulenda (Swahili) ^f,l^	**R60** ^γ,θ^	8	0.04	15	0.25	Cancers, wounds, fever, malaria, stomach pain, cardiovascular diseases, diabetes, viral diseases, parasitic diseases, gastrointestinal diseases, diabetes, hyperlipidemia, and hypertension.	†: [83] ‡: [27] ₸: [84]
*Hylodesmum repandum* (Vahl) H.Ohashi & R.R.Mill [*Desmodium repandum* (Vahl) Poir.] (Fabaceae, KIP000000638)	Irhuza (Shi) ^f,n^	**R61** ^β,ε^	41	0.20	6	0.10	Fever, ulcers, gastrointestinal diseases, malaria, stomach	†: NR ‡: NR ¥: [85]
*Hymenocardia acida* Tul (Phyllanthaceae, KIP464414491)	Kapempe (Shi) ^f,r^	**R62** ^β,μ^	11	0.05	5	0.08	Malaria, skin diseases, hemorrhoids, Parasites diseases, parasitic diseases, and diabetes	†: NR ‡: NR ₸: [86]
*Juglans nigra* L (Juglandaceae, KIP000000639)	Nusumbau (Bemba) ^d,r^	**R63** ^z,μ^	12	0.06	5	0.08	Parasitic diseases, gastrointestinal diseases, diabetes, ulcers	†: NR ‡: NR ₸: [87]
*Khaya nyasica* Stapf ex Baker f (Meliaceae, KIP000001372)	Mbamba (Luba-kat) ^b,r^	**R64** ^y, ε^	36	0.17	8	0.13	Cancers, diabetes, Drepanocytosis, ulcers, hepatic diseases, gastrointestinal diseases	†: NR ‡: NR ₸: [33,38]
*Kigelia aethiopum* (Fenzl) Dandy [*Kigelia africana* subsp. Africana] (Bignoniaceae, KIP000001376)	Kivungwe (Tabwa) ^f,r^	**R65** ^y, ε^	27	0.13	7	0,12	Wounds, fever, gastrointestinal diseases, skin diseases, respiratory diseases, cancers	†: NR ‡: NR ₸: [87]
*Kigelia africana* (Lam.) Benth (Bignoniaceae, KIP000000640)	Kivungwila (Luba-kat) ^f,r^	**R66** ^β,θ^	51	0.25	10	0.17	Malaria, Wounds, fever, gastrointestinal diseases, skin diseases, respiratory diseases, cancers, ulcers, urinary tract infection	†: NR ‡: [32] ₸: [87]
*Leonotis nepetifolia* (L.) R.Br (Lamiaceae, KIP000000641)	Kacumucumu (Shi) ^g,l^	**R67** ^γ, θ^	18	0.09	10	0.17	Fever, cough, malaria, rheumatism, hypertension, gastro-intestinal diseases, skin diseases, respiratory diseases, pain	†: NR ‡: NR ¥: [88]
*Leucas martinicensis* (Jacq.) R.Br. (Lamiaceae, KIP000000642)	Kanyamafundo (Shi) ^f,l^	**R68** ^v, θ^	6	0.03	9	0.15	kidney disorders, rheumatism, cough, diarrhea, fevers, gastro-intestinal diseases, skin diseases, neurodegenerative disorders	†: NR ‡: [58] ₸: [65]
*Luffa cylindrica* (L.) M.Roem [*Cucumis melo* L] (Cucurbitaceae, KIP000111051)	Kikenda mbayo (Luba-kat) ^f,l^	**R69** ^z, μ^	17	0.08	11	0.18	Respiratory diseases, urinary tract infection, ulcers, Parasites diseases, hypertension, hyperlipidemia, inflammatory diseases, viral diseases, diabetes, wounds	†: [41,89] ‡: [58] ¥: [56]
*Malus domestica* (Suckow) Borkh (Rosaceae, KIP000000643)	Chungwa nuni (Swahili) ^d,r^	**R70** ^z,ε^	6	0.03	6	0.10	hypertension, diabetes, hepatic diseases, diarrhea, fevers	†: NR ‡: [52] ₸: [43]
*Millettia griffoniana* Baill. (Fabaceae, KIP000000644)	Umuzimbe (Shi) ^f,r^	**R71** ^β, ε^	16	0.08	4	0.07	Cancers, viral diseases, Inflammatory diseases	†: [90] ‡: [91] ₮: [92]
*Ochna schweinfurthiana* F.Hoffm. (Ochnaceae, KIP000000645)	Ikyoni (Bemba) ^f,r^	**R72** ^β, ε^	7	0.03	8	0.13	Drepanocytosis, helminth, typhoid fever, malaria, skin diseases, respiratory diseases, wounds	†: NR ‡: [93,94] ₸: [94]
*Ocimum americanum* L (Lamiaceae, KIP000000646)	Lueni (Bemba) ^i,l^	**R73** ^x, μ^	15	0.07	5	0.08	Wound, cough, malaria, cough	†: NR ‡: [32] ₸: [33]
*Paramollugo nudicaulis* (Lam.) Thulin (Molluginaceae, KIP000000647)	Eswani (Tabwa) ^i,l^	**R74** ^x, μ^	9	0.04	5	0.08	Coughs, fever, respiratory diseases, wounds	†: NR ‡: NR ₸: [95]
*Parinari curatellifolia* Planch. ex Benth. (Chrysobalanaceae, KIP000001385)	Mupundu (Luba-kat) ^f,r^	**R75** ^β, δ^	14	0.07	4	0.07	Diabetes, cancer, inflammatory diseases, malaria	†: NR ‡: NR ₸: [63]
*Pentaclethra macrophyll*a Benth. (Fabaceae, KIP000111062)	Bubala (Kirega) ^f,r^	**R76** ^y, ε^	11	0.05	9	0.15	Drepanocytosis, wounds, hemorrhoids, diabetes, hepatic diseases, helminth, diarrhea, hyperlipidemia	†: [96] ‡: [32,97] ₸: [98]
*Phyllanthus amarus* Schum. & Thonn. (Phyllanthaceae, KIP000111065)	Kavudi (Luba-kat) ^d,l^	**R77** ^γ, ε^	13	0.06	6	0.10	Wounds, stomach diseases, urinary tract infection, hepatic diseases, gastrointestinal diseases	: NR ‡: [32] ₸: [33]
*Phyllanthus microdendron* Müll.Arg. (Phyllanthaceae, KIP000000648)	Unono (Luba-kat) ^f,r^	**R78** ^γ, ε^	12	0.06	10	0.17	Wounds, stomach diseases, urinary tract infection, hepatic diseases, gastro-intestinal diseases, diabetes, viral diseases, cancers, malaria	†: NR ‡: NR ₸: [99]
*Phyllanthus muellerianus* (Kuntze) Exell (Phyllanthaceae, KIP277202454)	Modere (Sanga) ^f,p^	**R79** ^α, ε^	7	0.03	11	0.18	Wounds, stomach diseases, urinary tract infection, hepatic diseases, gastro-intestinal diseases, diabetes, viral diseases, cancers, malaria, hypertension	†: NR ‡: [100] ₸: [30,63]
*Phyllanthus ovalifolius* Forssk [Syn *Phyllanthus guineensis*] (Phyllanthaceae, KIP000000649)	Mupetualupe (Bemba) ^f,p^	**R80** ^γ, δ^	6	0.03	11	0.18	Wounds, stomach diseases, urinary tract infection, hepatic diseases, gastro-intestinal diseases, diabetes, viral diseases, cancers, malaria, hypertension	†: NR ‡: [25] ₸: [38,63]
		**R81** ^z,δ^						
*Physalis peruviana* L (Solanaceae, KIP000000650)	Imbuhu (Shi) ^d,n^	**R82** ^z,ε^	5	0.02	10	0.17	Cancer, diabetes, ulcers, malaria, asthma, rheumatism, gastro-intestinal diseases, pain, fever.	†: NR ‡: [58] ¥: [26,101]
*Piliostigma thonningii* (Schum.) Millne-Redhead (Fabaceae, KIP000000651)	Kifumbe (Bemba) ^f,r^	**R83** ^γ,δ^	3	0.01	9	0.15	Coughs, fever, respiratory diseases, wounds, sexually transmitted infections, malaria, typhoid fever, hemorrhoids	†: NR ‡: [32] ₸: [20,33]
*Plumbago zeylanica* L (Plumbaginaceae, KIP000111067)	Daunani (Zela) ^i,p^	**R84** ^γ,δ^	4	0.02	6	0.10	Hemorrhoids, inflammatory diseases, diabetes, hypertension, Drepanocytosis	†: [102,103] ‡: [25,103] ¥: [104]
*Psidium guajava* L. (Myrtaceae, KIP000301861)	Ipera (Shi) ^f,r^	**R85** ^β, δ^	7	0.03	12	0.20	Ulcer, cough, fever, wounds, skin diseases, malaria, hepatic diseases, helminth, diarrhea, hyperlipidemia, fungal diseases	†: [105] ‡: [32] ₸: [38]
*Ricinus communis* L (Euphorbiaceae, KIP000000652)	Mubalika (Bemba) ^b,p^	**R86** ^w, ε^	9	0.04	5	0.08	bilharziasis, gastro-intestinal diseases, hepatic diseases, helminth	†: [106] ‡: [27] ₸: [87]
*Securidaca longepedunculata* Fresen (Polygalaceae, KIP000000653)	Lupapi (Bemba) ^f,r^	**R87** ^β, ε^	8	0.04	16	0.27	sexually transmitted infections, malaria, typhoid fever, hemorrhoids, coughs, epilepsy, gastro-intestinal diseases, headaches, skin diseases, hyperlipidemia, sexually transmitted infections, rheumatism, aphrodisiac, epilepsy, tuberculosis, ulcers, stomach pain	†: NR ‡: [107] ₸: [38,63]
*Senegalia polyacantha* (Willd.) Seigler & Ebinger [Syn *Acacia polyacantha* Willd] (Fabaceae, KIP000000654)	Kibombolo (Tabwa) ^h,r^	**R88** ^γ,θ^	4	0.02	8	0.13	snakebites, malaria, diabetes, wounds, skin diseases, bilharziasis, gastrointestinal diseases	†: NR ‡: [108] ₸: [43]
*Senna occidentalis* (L.) Link. (Fabaceae, KIP000000655)	Lukunda bajani (Luba-kas) ^f,q^	R89 ^γ,ε^	5	0.02	9	0.15	Diarrhea, dysentery, constipation, fever, sexually transmitted infections, malaria, diabetes, hepatic diseases	†: [109] ‡: [32] ₸: [38]
		**R90** ^v, λ^						
*Sida acuta* Burm.f (Malvaceae, KIP000000656)	Tumfumfu (Kikongo) ^f,l^	**R91** ^γ,ε^	21	0.10	7	0.12	Malaria, ulcers, constipation, diabetes, hemorrhoids, coughs	†: [110] ‡: [111,112] ¥: [88]
		**R92** ^v, λ^						
*Solanum incanum* L (Solanaceae, KIP000000657)	Ndulwe (Bemba) ^i,q^	**R93** ^γ, δ^	42	0.20	6	0.10	Viral Parasitic diseases, gastro-intestinal diseases, skin diseases, Inflammatory diseases	†: NR ‡: [113] ₸: [86]
*Stereospermum kunthianum* Cham (Bignoniaceae, KIP000000658)	Kasengele (Lamba) ^f,p^	**R94** ^γ,δ^	12	0.06	6	0.10	ulcers, skin diseases, Inflammatory diseases, respiratory diseases, sexually transmitted infections	†: NR ‡: [27] ₮: [114]
*Strychnos spinosa* Lam (Loganiaceae, KIP000111072)	Kisongole (Bemba) ^i,p^	**R95** ^z,δ^	5	0.02	5	0.08	Malaria, diabetes, snakebites, sexually transmitted infections	†: NR ‡: [115] ₸: [20,33,38]
*Syzygium cordatum* Hochst. ex C.Krauss (Myrtaceae, KIP000111074)	Cinyambaganyi (Tabwa) ^h,r^	**R96** ^z,ε^	7	0.03	9	0.15	Malaria, tuberculosis, diabetes, snakebites, sexually transmitted infections, wounds, and respiratory diseases	†: NR ‡: [41,116] ₸: [33]
*Tagetes minuta* L (Asteraceae, KIP000000659)	Mbaje mbuni (Zela) ^d,l^	**R97** ^γ, ε^	9	0.04	5	0.08	stomach pain, coughs, gastrointestinal diseases, pain	†: NR ‡: [58] ¥: [65]
*Thespesia garckeana* F.Hoffm. (Malvaceae, KIP000000660)	Makole (Sanga) ^f,r^	**R98** ^α, δ^	11	0.05	6	0.10	Fever, headache, stomach pain, diabetes, and inflammatory diseases	†: NR ‡: [19] ¥: [19]
*Trema orientale* (L.) Blume (Cannabaceae, KIP000000661)	Nalita (Luba-kat) ^i,p^	**R99** ^γ,θ^	34	0.16	6	0.10	Asthma, fever, cough, Inflammatory diseases, malaria	†: [117] ‡: [32] ₸: [65]
*Uapaca kirkiana* Müll. Arg (Phyllanthaceae, KIP000000662)	Masuku (Bemba) ^f,p^	**R100** ^z,μ^	15	0.07	11	0.18	stomach pain, dysentery, sexually transmitted infections, diabetes, wounds, hyperlipidemia, hypertension, Drepanocytosis, helminth, typhoid fever	†: NR ‡: [19] ₸: [38]
*Vitex congolensis var. thomasii* (De Wild.) Meerts [syn *Vitex thomasii* De Wild.] (Lamiaceae, KIP000000663)	Mufutu mukwa (Luba-kat) ^a,r^	**R101** ^β,ε^	36	0.17	5	0.08	Cancer, Inflammatory diseases, diabetes, gastrointestinal diseases,	†: NR ‡: NR ₸: [118]
*Xylopia aethiopica* (Dunal) A.Rich (Annonaceae, KIP000000664)	Njilu luba (Luba-kat) ^f,r^	**R102** ^β,ε^	7	0.03	11	0.18	rheumatism, diarrhea, dysentery, cough, asthma, hemorrhoids, malaria, diabetes, wounds, sexually transmitted infections	†: [119] ‡: [32] ₸: [38]
*Zanthoxylum chalybeum* Engl. (Rutaceae, KIP000001016)	Pupwe (Bemba) ^f,p^	**R103** ^α,μ^	10	0.05	7	0.12	Drepanocytosis, cancer, malaria, diabetes, tuberculosis, gastrointestinal diseases	†: NR ‡: [25] ₸: [33,63]
*Zanthoxylum zanthoxyloides* (Lam.) Zepern. & Timler (Rutaceae, KIP000000665)	Pupwe bimpa (Lamba) ^f,r^	**R104** ^β,ε^	7	0.03	6	0.10	Drepanocytosis, malaria, fever, tuberculosis, constipation	†: NR ‡: [32] ₮: [120]
*Zingiber officinale* Roscoe (Zingiberaceae, KIP000111077)	Tangawisi (Swahili) ^d,o^	**R105** ^α,θ^	9	0.04	10	0.17	Drepanocytosis, cancer, malaria, diabetes, tuberculosis, gastro-intestinal diseases, wounds, Inflammatory diseases, aphrodisiac	†: [121] ‡: [32,37] ₸: [33]
*Ziziphus abyssinic*a Hochst. ex A.Rich. (Rhamnaceae, KIP000000666)	Sula (Bemba) ^f,r^	**R106** ^α,μ^	14	0.07	9	0.15	Drepanocytosis, cancer, diabetes, gastrointestinal diseases, tuberculosis, malaria, wounds, and hypertension	†: NR ‡: [58] ₸: [38]
*Ziziphus mucronata* Willd (Rhamnaceae, KIP000000667)	Kankona (Bemba) ^f,r^	**R107** ^v,θ^	12	0.06	10	0.17	Drepanocytosis, cancer, diabetes, gastrointestinal diseases, tuberculosis, malaria, wounds, hypertension, stomach pain.	†: NR ‡: [122] ₸: [38,63]

***Legend*:**

† (uterotonic Activity)

‡ (uterotonic uses)

₸ (medicinal uses in Lubumbashi)

¥ (medicinal uses in DRC outside Lubumbashi)

₮ (medicinal uses outside DRC)

UP: used part, P: Preparation

–***Geographical type*** – CA: a, EA: b; NA-MA: c; NAT: d; NA-TA-SA-MA: e; TA: f; TA-MA: g; TA-SA: h; TA-SA-MA: I; WA: j; WA-SA: k- NA: North Africa; CA: Central Africa; TA: tropical Africa; SA: southern Africa; MA: Madagascar. ***Used part***: Bulb: u; Flowers: v; Seeds: w; Whole plant: x; Stem bark: y; Fruits: z; Root: α; Root bark: β; Leaves: γ ***Morphological type*** -Annual herb: l; Bulbous geophyte: m; Perennial herb: n; Rhizomatous geophyte: o; Shrub: p; Subshrub: q; Tree: r; Tuberous subshrub: s–***- Preparation method*** – Cataplasm: δ; Decoction: ε; Infusion: θ; Liniment: λ; Maceration: μ; Ointment: ψ. A ***cataplasm*** is a medicinal preparation that is applied directly to the skin. The suitability of the plant for this purpose is determined by the condition of the skin. Alternatively, they can be heated in water to soften them, and then gently crushed to extract the active ingredients. ***Liniment*** is a composition of oil (palm or olive) and plant powder in the proportions of 5:2. The ointment is applied to the vaginal muscle. ***Ointment*** is a preparation obtained from pork fat with a paste of the plant (parts 3:1). According to informants, on average, childbirth occurs within 30 minutes to two hours after the topical application of the herbal preparation.

#### 3.1.1. Taxonomic diversity of medicinal plants recorded.

**Fig 2:**
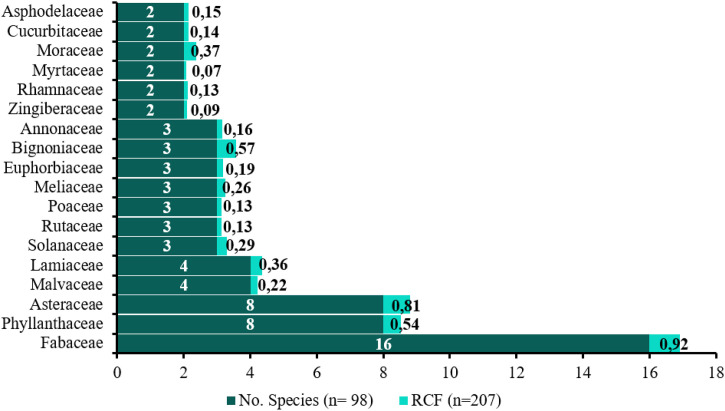
Relative citation frequencies of plant families with n ≥ 2 plants.

The 98 plant species identified in this survey are distributed across 98 genera and 43 botanical families. The most represented family is Fabaceae, with a relative citation frequency (RCF) of 0.92, encompassing 16 species. This is followed by Asteraceae (RCF: 0.81, 8 species), and Phyllanthaceae (RCF: 0.54; 8 species (Fig 2).

#### 3.1.2. Variation in geographical, morphological types, and nomenclature.

**Fig 3:**
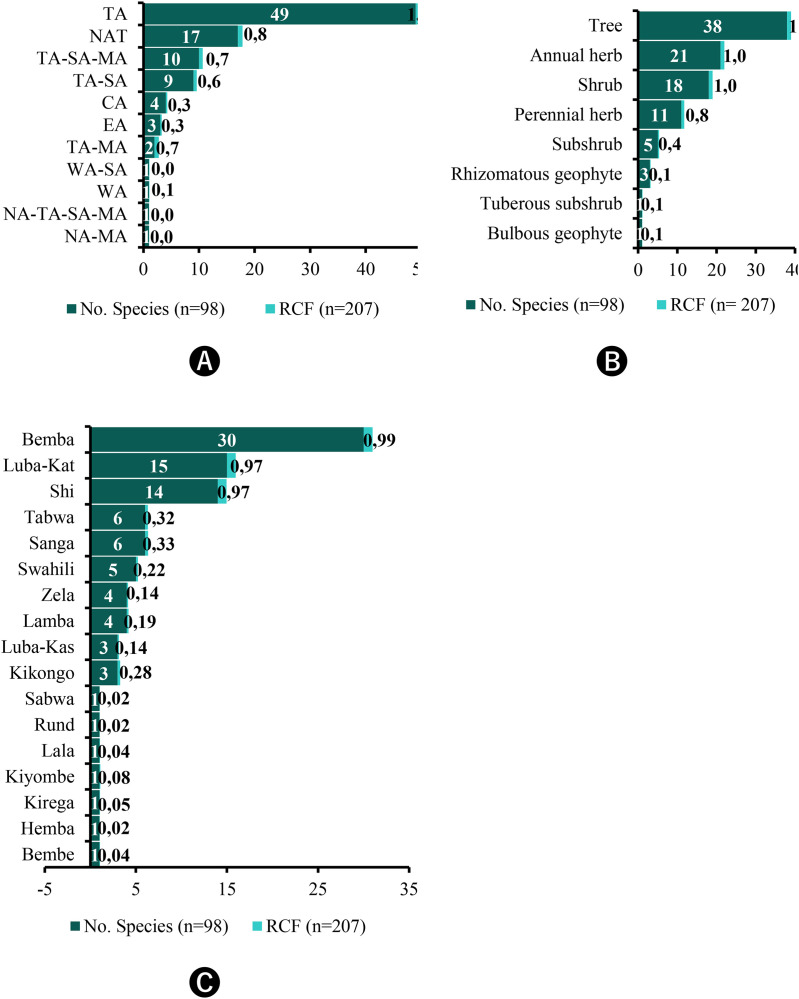
Morphological (a) and geographical (b) type of plants, category of informants (c), and Languages of Local Plant Names (C).

The plant species documented in our ethnobotanical survey are classified into 11 geographical types. The TA type is the most prevalent, with 49 species, and an RCF of 1.0. They exhibit eight distinct morphological types, with the tree form (38 species; RCF: 1.0) as the most prevalent, followed by annual herbs (21 species; RCF: 1.0) and shrubs (18 species; RCF: 1.0). These species have names in 17 local Congolese languages. The most frequently used language is Bemba (30 species; RCF: 0.99), followed by Luba-Katanga (15 species; RCF: 0.97), and Shi (14 species; RCF: 0.97) (Fig 3).

### 3.2. Profile of herbal recipes used as uterotonics

The data collected on 98 plant species during ethnobotanical surveys, documented in this study, that are traditionally used in Lubumbashi to facilitate childbirth led to the identification of 122 uterotonics formulations. Among these, 107 recipes use a single plant (Table 1), whereas 15 formulations incorporate two plant species (Table 2).

**Table 2:** Traditional polyherbal formulations used as uterotonics.

N°	Specie 1	Specie 2	N° Recipe	UP (Ratio)	Preparation	Size (RCF) (n = 207)
1	*Abrus precatorius*	*Aloe vera*	**R108**	Sd-Lv (2 ÷ 1)	Cataplasm	10(0.05)
2	*Annona senegalensis*	*Ageratum conyzoides*	**R109**	Sb-Wp (1 ÷ 2)	Cataplasm	12(0.06)
3	*Aframomum alboviolaceum*	*Alchornea floribunda*	**R110**	Fr-Lv (2 ÷ 3)	Decoction	15(0.07)
4	*Antidesma venosum*	*Azadirachta indica*	**R111**	Rb-Fr (1 ÷ 1)	Ointment	18(0.09)
5	*Boerhavia diffusa*	*Cajanus cajan*	**R112**	Lv- Fr (3 ÷ 1)	Cataplasm	22(0.11)
6	*Cissus schmitzii*	*Clausena anisata*	**R113**	R-Lv (2 ÷ 1)	Infusion	27(0.13)
7	*Combretum molle*	*Commelina africana*	**R114**	Lv-Sb (2 ÷ 1)	Maceration	30(0.15)
8	*Cucurbita pepo*	*Cynodon dactylon*	**R115**	Fr-Wp (2 ÷ 1)	Decoction	18(0.09)
9	*Ekebergia capensis*	*Erythrina abyssinica*	**R116**	R-Sb (2 ÷ 1)	Decoction	35(0.17)
10	*Ficus capensis*	*Flueggea virosa*	**R117**	Sb-Lv (2 ÷ 1)	Cataplasm	31(0.15)
11	*Grona adscendens*	*Asparagus africanus*	**R118**	Lv-Lv (2 ÷ 3)	Cataplasm	18(0.09)
12	*Hymenocardia acida*	*Juglans nigra*	**R119**	Rb-Fr (3 ÷ 2)	Cataplasm	68(0.33)
13	*Khaya nyasica*	*Kigelia aethiopum*	**R120**	Sb-Sb (3 ÷ 2)	Cataplasm	18(0.09)
14	*Malus domestica*	*Millettia griffoniana*	**R121**	Fr-Rb (3 ÷ 2)	Cataplasm	22(0.11)
15	*Ocimum americanum*	*Piliostigma thonningii*	**R122**	Lv-Lv (3 ÷ 2)	Cataplasm	27(0.13)

***Legend*** – PU-Ratio: Part used and proportion of the mixture; Lv: leaf; R: root; Rb: Root bark; Sb: Stem bark; Fr: fruit; Sd: Seed; N° Recipe. = uterotonic recipe.

**Fig 4:**
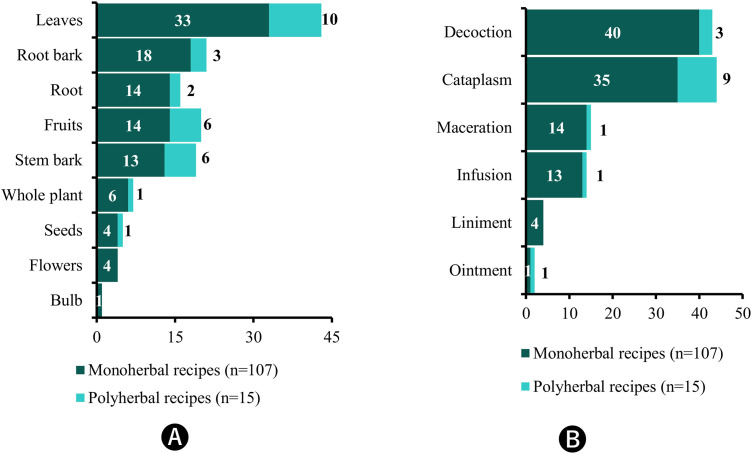
Parts used (A) and mode of preparation of traditional uterotonics formulations (B).

These preparations utilize nine different plant organs, with leaves being the most frequently used (33 monoherbal recipes and 10 polyherbal recipes), followed by root bark (18 monoherbal recipes and 3 polyherbal recipes) (Fig 4).

There are six different preparation methods for plant organs, with decoction (40 recipes using a single plant, and 3 using two plants) and cataplasm (35 and 9 recipes) being the most used methods (Fig 4).

In polyherbal formulations, no plant species out of 30 is recurrent across multiple distinct preparations. The most frequently utilized formulation, designated as R199, consists of a combination of *Hymenocardia acida* root bark, and *Juglans nigra* fruits, exhibiting a relative citation frequency (RCF) of 0.33. This formulation is followed by R116, which is derived from the mixture of *Ekebergia capensis* roots, and *Erythrina abyssinica* stem bark (Table 2).

### 3.3. Other human ailments treated by inventoried species

Beyond their role in facilitating childbirth by producing a uterotonic effect, the 98 documented plant species are also utilized in Lubumbashi folk medicine for the treatment of 60 additional ailments. Among these, gastrointestinal diseases, diabetes, wounds, and malaria represent the three primary indications, both in terms of citation frequency (over 100 citations), and botanical diversity, with each condition involving more than 40 plant species from the recorded cohort (Table 3).

**Table 3:** Other ailments treated by the surveyed plants.

Ailments (n = 61)	Ns	Ailments (n = 61)	Ns	Ailments (n = 61)	Ns
Atherosclerosis	1	Dengue fever	3	Dysentery	13
Cataract	1	Constipation	4	Urinary tract infection	14
Cholera	1	Gastritis	4	Hypertension	15
Cold	1	Cardiovascular diseases	5	Drepanocytosis	16
Conjunctivitis	1	Fungal ailments	5	Headache	19
Contraception	1	Intoxications	5	Inflammatory diseases	19
Dental problems	1	Leprosy	5	Hepatic diseases	20
Eye problems	1	Typhoid fever	5	Ulcers	20
HIV/AIDS	1	Hemorrhoids	6	Diarrhea	21
Kidney disorders	1	Parasites diseases	6	Sexually transmitted infections	21
Meningitis	1	sexual dysfunction	7	Coughs	25
Thrombosis	1	Asthma	7	Cancers	27
aphrodisiac	2	Hemorrhoids	7	Respiratory diseases	28
Dysmenorrhea	2	Hyperlipidemia	7	Stomach diseases	28
Epilepsy	2	Pain	7	skin diseases	29
Myoma	2	Rheumatism	8	Fever	37
Neurodegenerative disorders	2	Snake bites	8	gastrointestinal diseases	41
Pruritis	2	Tuberculosis	8	Diabetes	44
bilharziasis	3	Viral diseases	8	Wounds	44
Chickenpox	3	Helminths diseases	9	Malaria	46

***Legend***: Ns: Numbers of species. The intensity of the color ranges from red (low) to green (high), with yellow (medium) in between, corresponding to the number of species identified in the management of the corresponding condition, according to our informants.

### 3.4. UV and RCF of plant surveyed

The RCF of the plant species documented in this study ranges from 0.01 for *Piliostigma thonningii* to 0.25 for *Kigelia africana*, the species with the highest frequency. This is followed by *Crassocephalum vitellinum* (RCF: 0.23), *Hylodesmum repandum* (RCF: 0.20), and *Solanum incanum* (RCF: 0.20). The use value of the recorded species varies from 0.05 for *Alchornea floribunda* to 0.32 for *Securidaca longepedunculata*, the species demonstrating the highest utilization. Next are *Asparagus africanus* (UV = 0.27) and *Hibiscus sabdariffa* (UV = 0.25).

### 3.5. Socio-demographic profile of informants

The informants surveyed in this study fall into three main categories: housewives (49.8%), herbalists (12.6%) and traditional healers (37.6%). The majority are women (64.7%), with an average age of 41 years, ranging from 17 to 73. They predominantly reside in the Katuba (21.7%) and Annexe (19.8%) municipalities, and most have a secondary education (40.0%). The informants are distributed across six distinct occupational groups, with traditional healers the most numerous (37.5%). Furthermore, a significant proportion (45.4%) have over 11 years’ experience applying medicinal plants to facilitate childbirth (Table 4).

**Table 4:** General characteristics of the informants.

Class	Category	Housewives (n = 103)	Herbalists (n = 26)	TH (n = 78)	All Informants (n = 207)
		**n**_**i**_ **(%)**	**n**_**i**_ **(%)**	**n**_**i**_ **(%)**	**%**
Age	15-25	02**(06.3)**	00**(00.0)**	01**(07.1)**	**06.2**
	26-35	14**(32.9)**	01**(12.5)**	03**(21.4)**	**32.1**
	36-45	81**(48.6)**	05**(62.5)**	02**(14.3)**	**47.7**
	46-55	04**(09.5)**	01**(12.5)**	02**(14.3)**	**09.7**
	>55	02**(02.7)**	01**(12.5)**	06**(42.9)**	**04.3**
Experience	1-5	65**(05.4)**	00**(00.0)**	01**(07.1)**	**05.4**
(Year)	6-10	25**(33.7)**	01**(12.5)**	06**(42.9)**	**33.6**
	11-15	05**(46.2)**	04**(50.0)**	03**(21.4)**	**45.4**
	16-20	05**(12.0)**	02**(25.0)**	02**(14.3)**	**12.3**
	21-25	03**(02.7)**	01**(12.5)**	02**(14.3)**	**03.3**
Profession	Trade	20**(19.4)**	00**(00.0)**	00**(00.0)**	**09.7**
	Public sector	21**(20.4)**	00**(00.0)**	00**(00.0)**	**10.1**
	Liberal	59**(57.3)**	00**(00.0)**	00**(00.0)**	**28.5**
	Housework	03**(02.9)**	00**(00.0)**	00**(00.0)**	**01.5**
	TH	00**(00.0)**	00**(00.0)**	78**(100)**	**37.7**
	Herbalist	00**(00.0)**	26**(100)**	00**(00.0)**	**12.5**
Gender	Female	77**(74.8)**	16**(61.5)**	41**(52.6)**	**64.7**
	Male	26**(25.2)**	10**(38.5)**	37**(47.4)**	**35.3**
Municipality of residence	Annexe	23**(22.3)**	02**(07.7)**	16**(20.5)**	**19.8**
	Kamalondo	13**(12.6)**	00**(00.0)**	02**(02.5)**	**07.2**
	Kampemba	15**(14.6)**	08**(30.8)**	12**(15.4)**	**16.9**
	Katuba	12**(11.7)**	10**(38.5)**	23**(29.5)**	**21.7**
	Kenya	16**(15.5)**	02**(07.7)**	12**(02.5)**	**14.5**
	Lubumbashi	18**(17.5)**	02**(07.7)**	03**(03.8)**	**11.2**
	Ruashi	06**(05.8)**	02**(07.7)**	10**(12.8)**	**08.7**
Level of studies	None	24**(23.3)**	02**(07.7)**	05**(06.4)**	**15.0**
	Primary	21**(20.4)**	08**(30.8)**	23**(29.5)**	**25.1**
	Professional	25**(24.3)**	03**(11.5)**	01**(01.3)**	**14.1**
	Secondary	26**(25.2)**	10**(38.5)**	47**(60.2)**	**40.0**
	University	07**(06.8)**	03**(11.5)**	02**(02.6)**	**05.8**

***Legend –*** TH: Traditional healer. Ni: number of Informants. The percentages for each category are shown in bold, as is the overall percentage. Each time, refer to n to determine it. ***Traditional healers and Herbalist***: Unlike herbalists, who are primarily involved in the commercial collection and distribution of plants, traditional practitioners draw on ancestral knowledge to treat illnesses. In addition to botanical resources, they use a wide range of natural therapeutic agents. They may also turn to herbalists to obtain specialized raw materials.

## 4. Discussion

### 4.1. Diversity of medicinal plants used as uterotonic in Lubumbashi

For millennia, the inhabitants of Lubumbashi have used the flora of the Miombo woodlands to treat various health issues, particularly to facilitate childbirth as uterotonic. In the current investigation, 98 plant varieties were documented according to their application by housewives, herbalists, and traditional healers to support childbirth.

A systematic review of the available literature (Table 1) enabled these taxa to be divided into two main categories, which can be subdivided into five subclasses. The first category comprises 34 species newly documented to have oxytocic uses, while the second category includes 64 species for which oxytocic applications have already been reported in previous studies (Table 1).

The 34 species in the first category were organized into three subclasses:

The first subclasses with nineteen species that are already recognized as medicinal plant in Democratic Republic of Congo, particularly around Lubumbashi, namely: *Aframomum alboviolaceum, Albizia adianthifolia, Antidesma venosum, Bobgunnia madagascariensis, Cissus schmitzii, Combretum molle, Crassocephalum montuosum, Cyanthillium cinereum, Ficus stuhlmannii, Flueggea virosa, Harungana madagascariensis, Hexalobus monopetalus, Hymenocardia acida, Juglans nigra, Khaya nyasica, Kigelia aethiopica, Parinari curatellifolia, Phyllanthus microdendron,* and *Vitex congolensis var. thomasii*;

The second subclass with Eight species documented as medicinal elsewhere in DRC, but not previously reported from Lubumbashi: *Adenia gummifera, Albizia lebbeck, Alchornea floribunda, Baphiopsis parviflora, Boerhavia diffusa, Cassia sieberiana, Hylodesmum repandum*, and *Leonotis nepetifolia*.

The third subclass with seven species whose medicinal use has been established outside the DRC: *Afrothismia mhoroana*, *Aloe chabaudii*, *Anchusa ovata, Bombax ceiba, Cochlospermum planchonii, Grona adscendens*, and *Paramollugo nudicaulis*. Together, these 34 taxa are key to understanding the specific role of Lubumbashi’s traditional medicine in managing difficult deliveries. Their documentation paves the way for targeted pharmacological research to validate the reported oxytocic effects and enrich the local pharmacopoeia with robust scientific evidence.

Although none of the 34 species catalogued in Lubumbashi have, to date, been explicitly reported or validated for oxytocic activity in the accessible literature, several taxa within the same genera exhibit well-documented uterotonic uses. For example, within the genus Aframomum, *A. melegueta* K. Schum. (also known as Ataare) is traditionally chewed by the Akoko people of Nigeria to facilitate childbirth [123]. Four species in the genus Albizia have been the subject of ethnobotanical investigations: *Albizia zygia* (DC.) J.F.Macbr: Stem bark is used as a purgative in the Ivory Coast to facilitate childbirth [124]; *Albizia chinensis* Lam.: triterpenoid saponins isolated from the roots significantly modulate uterine contractions [125]; *Albizia ferruginea* (Guill. & Perr.) Benth.: a stem bark decoction is used in Nigeria to aid childbirth [126]; *Albizia gummifera* (J. F. Gmel.) C. A. Meyer: The glycoside ‘albitocin’, isolated from the bark, increases the frequency and amplitude of spontaneous uterine contractions in various animal models [127]. Additional genera have likewise yielded extracts with demonstrated oxytocic activity: aqueous stem-bark extract of *Adenia globosa* Engl. [128]; methanolic leaf extract of *Alchornea laxiflora* (Benth.) Pax: [73]; aqueous leaf extract of *Cassia fistula* L. [129]; aqueous leaf extract of *Cassia italica* Mill [130]; and aqueous stem bark extract of *Vitex doniana* Sweet [131]. These pharmacological evidence of oxytocic activity in plants of the same genus reported in our current investigations suggest that it is possible to identify plants among those in the inventory that also have proven oxytocic potential. This highlights the importance of conducting targeted pharmacological investigations on Lubumbashi inventory species to confirm and characterize their oxytocic potential.

Within the second category, two subclasses were delineated: The fourth subclass (n = 34) comprises taxa that have traditionally been utilized as medicinal plants, yet for which no pharmacological evidence has been documented thus far. The fifth subclass (n = 30) comprises taxa for which uterotonic activity has been documented in the scientific literature. The confirmed uterotonic effects observed in the fifth subclass serve as empirical validation of the ethnobotanical survey data and strongly suggest that a similar proportion of the unstudied taxa may also possess active uterotonic principles.

The fourth subclass comprises 38 species: *Aerva javanica*, *Ageratum conyzoides*, *Annona senegalensis*, *Asparagus africanus*, *Capsicum annuum*, *Clausena anisata*, *Clematis scabiosifolia*, *Crassocephalum vitellinum*, *Cucurbita pepo*, *Cynodon dactylon*, *Dalbergia boehmii*, *Diplorhynchus condylocarpon*, *Elaeis guineensis*, *Eleusine indica*, *Erythrina abyssinica*, *Euphorbia hirta*, *Ficus capensis*, *Leucas martinicensis*, *Malus domestica*, *Ochna schweinfurthiana*, *Ocimum americanum*, *Phyllanthus amarus*, *Phyllanthus ovalifolius*, *Physalis peruviana*, *Piliostigma thonningii*, *Securidaca longepedunculata*, *Senegalia polyacantha*, *Solanum incanum*, *Stereospermum kunthianum*, *Strychnos spinosa*, *Syzygium cordatum*, *Tagetes minuta*, *Thespesia garckeana*, *Uapaca kirkiana*, *Zanthoxylum chalybeum*, *Zanthoxylum zanthoxyloides*, *Ziziphus abyssinica,* and *Ziziphus mucronata*.

Many of these species are already recorded in diverse African pharmacopoeias for their uterotonic properties, substantiating an ethnomedicinal consensus on their use to facilitate parturition. This interregional convergence implies the conservation of bioactive uterotonic constituents within these species, and justifies further studies, including *ex vivo* assays on isolated uterine muscle, *in vivo* evaluation in gravid rodent models, and the isolation and structural elucidation of the responsible metabolites. Notably, only five of these species (approximately 13%), those indicated above, have not previously been documented in the traditional lushoise pharmacopoeia. This relatively low proportion highlights the depth and coherence of local botanical knowledge while pinpointing novel candidate species for prioritized pharmacological screening.

### 4.2. Botanical families, morphology, and endemicity of inventoried plants

Of the 98 taxa documented in our ethnobotanical surveys, three species predominate (39%), nearly half are endemic to tropical Africa (49%), and 16% belong to the Fabaceae family. The overrepresentation of Fabaceae trees endemic to tropical Africa corroborates earlier ethnobotanical investigations in the katangense region [20,33,63,86,132,133]. Fabaceae trees exhibit a suite of physiological and ecological traits conferring resilience in hot intertropical environments. Symbiotic associations with rhizobia enable efficient biological nitrogen fixation, permitting establishment on leached, nutrient‐poor tropical soils [134]. A deep, laterally branching taproot system enhances water uptake during prolonged dry seasons. Foliar adaptations, compound leaf architecture, and a thick cuticle, minimize transpirational water loss while preserving high photosynthetic capacity under intense insolation [135,136].

Moreover, pronounced phenological plasticity, characterized by leaf abscission in the dry season followed by rapid foliar flush at the onset of rain, synchronizes growth with favorable moisture regimes, and underpins the ubiquity of Fabaceae in intertropical woody communities. Moreover, extant literature validates the existence of a probable correlation between the characteristic phytochemical composition of Fabaceae – notably their richness in triterpenoid, and steroidal saponins, and estrogenic flavonoids (phytoestrogens) – and their documented uterotonic activity. Indeed, preclinical trials have demonstrated that saponin-rich plant fractions induce, in a dose-dependent manner, an increase in the frequency and amplitude of contractions of isolated uterine smooth muscle, thus replicating the traditional use of these plants to facilitate labor [137]. Furthermore, phylogenetic analyses reveal that the Fabaceae lineages most enriched in estrogenic flavonoids, dedicated to fertility and parturition drugs, coincide with the “warm environments” [138]establishing a chemical basis likely to underpin uterotonic efficacy across this family.

Among the documented plant species, spontaneous, uncultivated varieties account for 91 species (92.8%). These wild plants are predominantly used in traditional medicine, offering substantial advantages in terms of accessibility and affordability. This makes them invaluable for healthcare systems with limited resources, such as those of the Democratic Republic of Congo [139,140]. However, utilizing them presents significant challenges. The chemical composition of these plants varies greatly due to environmental influences, which can affect their therapeutic efficacy and safety. Furthermore, the lack of standardization in most cases increases the risk of toxicity and complicates quality assessment. Furthermore, the excessive harvesting of certain species threatens biodiversity, necessitating the implementation of conservation strategies [141,142]. Therefore, although these plants have significant medicinal potential, their integration into healthcare practices necessitates rigorous scientific research and robust regulatory mechanisms to guarantee their safety and efficacy within a structured framework.

### 4.3. Uterotonic recipes of medicinal plant survey

In the 122 uterotonic recipes inventoried during our surveys, most of which used a single plant, the leaf (35%) was the most used organ, and decoction (35%) the most prevalent method of preparation in mono-herbal formulation. However, among all the recipes, poultices are the most common method, which is justified by the fact that most recipes are for local application. While decoction is the most common method of preparation in several ethnobotanical studies carried out in the region [63,20, 132-133–,^143^–^149^]Various organs seem to be the most popular according to the studies. Both the leaves [33,63,86,132,144,150,151], sometimes roots [86,118,145,146,149] or stem bark [20,152,153].

The use of leaves in traditional medicine offers several significant advantages over other medicinal plant organs, both pharmacologically and ecologically. From a therapeutic point of view, leaves are often rich in the synthesis of most bioactive metabolites [43,154,155]. In addition, their anatomical structure facilitates the extraction of active ingredients, improving the efficacy of traditional preparations [43]. Ecologically, leaf harvesting is generally less destructive than root or stem bark harvesting, contributing to the preservation of plant species and the sustainability of medicinal practices [156]. Moreover, leaves are often available in greater quantities and over a longer period of the year, making them more accessible to local communities. However, their active ingredient content can vary according to season and environmental conditions, requiring rigorous standardization to guarantee their therapeutic efficacy.

During this study, fifteen polyherbal formulations (12%) each combine two plant species, an approach with ambivalent outcomes. On one hand, phytotherapy exploits the synergy of bioactive constituents and mutual masking of adverse effects to achieve a broader therapeutic spectrum with reduced toxicity. On the other hand, the complexity of these mixtures can prove counterproductive: unforeseen interactions, excessive dilution of key active compounds, and botanical variability related to geographic origin or harvest season all impede the standardization and reproducibility of treatments [33,153]. Added to these challenges are the risks of cross-allergies among multiple species and the uncertain safety of compositions that lack robust clinical validation, which may reveal toxic or mutagenic properties. Nevertheless, the enduring use of most of these formulations for over twenty-five years attests to their acceptable safety profile and adequate efficacy: without a favorable benefit–risk balance, they would have been discontinued long ago.

### 4.4. Plants highlighted by this survey

**Fig 5:**
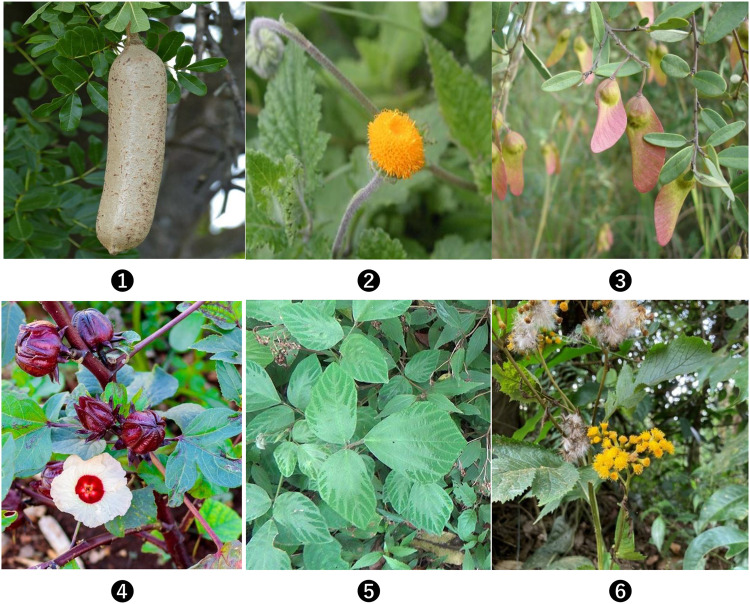
Plants highlighted in this study in order of appearance: Kigelia africana, Crassocephalum vitellinum, Securidaca longepedunculata, Hibiscus sabdariffa, Hylodesmum repandum, Crassocephalum montuosum.

In this study, we consider that the plants highlighted are notably the two most frequently cited plants: *Kigelia africana* (RCF: 0.25) & *Crassocephalum vitellinum* (RCF: 0.23); the two most widely used plants: *Securidaca longepedunculata* (UV: 0.27) & *Hibiscus sabdariffa* (UV = 0.25) as well as the two plants most frequently cited among those reported for the first time as oxytocic plants thanks to our present study: *Hylodesmum repandum* (RCF: 0.20) & *Crassocephalum montuosum* (RCF: 0.19) (Fig 5).

Indeed, a high degree of citation convergence around a species reflects a high Informant Consensus Index. This homogeneity of knowledge suggests empirical pharmacological validation: the more a plant is cited for its oxytocic properties, the more likely it is to contain bioactive compounds that could account for these properties. Furthermore, the most frequently used plants define the actual Use Value (UV) within the community. In contexts where access to modern healthcare is limited, these species are the pillars of health in this environment. Studying them is a public health emergency, both to validate their safety and to prevent the risks of toxicity associated with non-standardized dosages. However, it should be noted that poorly documented plants are not insignificant; they reflect the endemic nature of knowledge and the bio-cultural specificity of the environment, which can very easily lead to original research that may result in new knowledge.

If a case had to be made for a single plant per category, such as most cited, most used or new discovery, then it would be the bark of *Kigelia africana*, the leaves of *Securidaca longepedunculata* and the leaves of *Hylodesmum repandum*, which are the plants that stand out most in this study (Fig 5).

The stem bark of *Kigelia africana* (Lam.) Benth. is a key part of African traditional medicine, where it has been used to treat a wide range of illnesses for a long time. As we all know, traditional applications cover a range of dermatological disorders, like eczema and wound healing, as well as gastrointestinal disturbances, including dysentery [30,153]. They also cover critical gynaecological indications, like labour induction and control of postpartum haemorrhage [157,158]. Recent drug studies have shown that these traditional uses are legit and have reported strong antimicrobial, anti-inflammatory, and antioxidant effects, as well as the ability to kill melanoma and renal carcinoma cell lines [159,160]. These bioactivities arise from a range of phytochemicals, such as naphthoquinones (e.g., lapachol and kigelinone), iridoid glycosides (e.g., verminoside and specioside), and polyphenolic compounds (e.g., gallotannins and flavonoids) [161,162]. All this evidence shows that *K. africana* stem bark is important in terms of pharmacology, and that it should be further explored as a source of therapeutic agents.

Although the roots of *Securidaca longepedunculata* are most frequently employed in traditional medicine, the leaves are also used for a wide range of ethnomedical indications, including epilepsy, headache, abdominal pain, infertility, snakebite, toothache, cancer, dermal infections, dislocated jaw, contraceptive purposes, and placental expulsion [153,163]. Pharmacological investigations have provided evidence supporting several bioactivities of leaf extracts, notably antimicrobial activity [164,165], immunomodulatory and anti‑inflammatory effects [166], antiplasmodial activity [167], antioxidant properties [168], and anticonvulsant activity [169]. These activities have been correlated with the presence of diverse phytochemical classes, principally xanthones, saponins, and flavonoids [164,170], some of which have been reported to possess oxytocic potential [171–175].

The leaves of *Hylodesmum repandum* (syn. *Desmodium repandum*) are a cornerstone of traditional primary healthcare, widely utilized as hemostatic poultices for treating fresh wounds, ulcers, and skin infections, and in decoctions to manage fever, dysentery, and pain. These traditional applications are supported by ethnopharmacological studies demonstrating significant antimicrobial, antioxidant, and anti-inflammatory activities, particularly against pathogens such as Staphylococcus aureus, as well as antimalarial activity. This biological efficacy is attributed to a distinct phytochemical profile rich in flavonoids (specifically isoflavonoids), tannins, and alkaloids, groups also reported among those with uterotonic potential [172–174].

## 5. Limits of the study

The findings of this study should be interpreted in light of several limitations: (*i*) the absence of quantitative data on harvesting pressure and its potential impact on the population dynamics of highly sought‑after taxa; (*ii*) the lack of clinical validation for interview‑derived claims concerning therapeutic indications and mechanisms of action; (*iii*) the study’s emphasis on ethnomedicinal knowledge related to uterotonic uses combined with a limited geographic scope, which may have resulted in incomplete documentation of other local uses of the recorded taxa; (*iv*) the omission during field surveys of critical usage details, such as administration conditions, contraindications, dosage regimens, and adverse effects, underscoring the need for targeted pharmacological, toxicological, and dosage‑finding studies for each reported taxon; and (*v*) insufficient documentation of herbal formulations and ancillary traditional applications beyond obstetric use, which warrants systematic ethnobotanical, phytochemical, and pharmacological follow‑up investigations.

## 6. Conclusion

The findings of this study support that in Lubumbashi, both traditional healers and women community leaders routinely employ medicinal plants as uterotonic agents in the management of childbirth complications. The taxa identified for uterotonic use frequently possess additional ethnomedicinal applications, and several of the uses recorded locally correspond with reports from other African pharmacopoeias, indicating regional convergence in traditional obstetric practice.

These ethnobotanical findings provide a rationale for prioritized pharmacological and clinical investigation. Systematic phytochemical profiling, bioassay‑guided fractionation, and *in vitro* and *in vivo* evaluation of uterotonic activity are required to validate efficacy and to identify the bioactive constituents responsible for the observed effects. Concurrent toxicological and finding studies, together with documentation of traditional formulations and administration regimens, are essential to assess safety and translational potential.

Finally, given the dual importance of these species for maternal health and local livelihoods, future research should integrate conservation assessments and sustainable use strategies alongside biomedical validation to ensure that any development of plant‑derived uterotonics is both scientifically robust and culturally and ecologically responsible.

## Supporting information

S1 Table Survey questionnaire. (DOCX)

S1 Fig Graphical Abstract. (DOCX)
