# Supplementary material for: Ethnomedicinal knowledge of plants used in alternative medicine as uterotonics in Lubumbashi, Haut-Katanga, Democratic Republic of Congo
Source: PLoS One. 2026 Aug 14;21(8):e0355373. doi: 10.1371/journal.pone.0355373 (PMC13475857; doi:10.1371/journal.pone.0355373)
Supplement: Supplementary file 1 [file pone.0355373.s003.pdf]

PLOS One

Ethnomedicinal Knowledge of plants used in Alternative Medicine as uterotonic in Lubumbashi, Haut-Katanga (DR Congo).

--Manuscript Draft--

Manuscript Number:	PONE-D-26-11446
Article Type:	Research Article
Full Title:	Ethnomedicinal Knowledge of plants used in Alternative Medicine as uterotonic in Lubumbashi, Haut-Katanga (DR Congo).
Short Title:	Uterotonic medicinal plants from Lubumbashi
Corresponding Author:	Bashige Valentin Chiribagula, Ph.D<br>Université de Lubumbashi, UNILU<br>Lubumbashi, Haut-Katanga CONGO, THE DEMOCRATIC REPUBLIC OF THE
Keywords:	Kigelia africana,<br>Hylodesmum repandum,<br>Securidaca longepedunculata,<br>uterotonic plants,<br>Katangese traditional medicine (KTM).
Abstract:	Background<br>Traditional medicine constitutes a principal component of primary healthcare in Democratic Republic of Congo, particularly in Lubumbashi. However, ethnobotanical knowledge related to its use remains incompletely documented and underutilised. The present study aimed to systematically document medicinal plants used as uterotonic among residents of Lubumbashi.<br>Methods<br>Ethnobotanical data were collected through structured interviews with 207 informants (housewives, =103; herbalists, =26; traditional healers, =78; sex ratio M/F = 0.5; mean age 35.6 ± 4.3 years; mean professional experience 13 ± 4.2 years).<br>Results<br>Informants cited 98 medicinal plant species, predominantly trees (38 species), distributed across 43 botanical families, with Fabaceae being the most represented family (16 species). Malaria was the most frequently reported non-uterotonic indication (46 species). A total of 122 traditional oxytocic formulations were recorded; most involved leaves (43 recipes) and were principally administered as cataplasm (44 recipes). To our knowledge, 34 species were reported for the first time with oxytocic uses, of which eight are documented for the first time as medicinal plants in DRC. Among taxa, Hylodesmum repandum (relative citation frequency, RCF = 0.20) and Crassocephalum montuosum (RCF = 0.19) exhibited the highest citation frequencies, while Securidaca longepedunculata (use value, UV = 0.27) and Hibiscus sabdariffa (UV = 0.25) showed the greatest overall use values.<br>Conclusion<br>These findings provide a baseline inventory for pharmacological, phytochemical, and toxicological studies, and support prioritization of bioassay-guided validation and conservation strategies for culturally important species.
Order of Authors:	Bashige Valentin Chiribagula, Ph.D
	Okusa Ndjolo Philippe, Ph.D
	Cedrick Shakalenga Mutombo, Ph.D
	Manya Mboni Henry, Ph.D
	Numbi Wa Ilunga Evodie, Ph.D
	Bakari Amuri Salvius, Ph.D
	Jean Claude Nkwanga, M.D
	Muyumba Singoy Sarah, Ph.D Student
	Mwamba Maseho Faustin, M.D PhD Student

Powered by Editorial Manager® and ProduXion Manager® from Aries Systems Corporation

	Biayi Benaja Martin, M.D PhD Student
	Sangwa Kamulete Gregoire, M.D PhD Student
	Mbayo Kalubandika Glaubert, M.D PhD Student
	Muhona Melman, M.D PhD Student
	Lumbu Simbi Jean Baptiste, Ph.D
**Additional Information:**	
**Question**	**Response**
**Financial Disclosure**<br><br>Enter a financial disclosure statement that describes the sources of funding for the work included in this submission. Review the submission guidelines for detailed requirements. View published research articles from *PLOS ONE* for specific examples.<br><br>This statement is required for submission and **will appear in the published article** if the submission is accepted. Please make sure it is accurate.<br><br>**Funded studies**<br>Enter a statement with the following details:<br>• Initials of the authors who received each award<br>• Grant numbers awarded to each author<br>• The full name of each funder<br>• URL of each funder website<br>• Did the sponsors or funders play any role in the study design, data collection and analysis, decision to publish, or preparation of the manuscript?<br><br>Did you receive funding for this work?	The author(s) received no specific funding for this work.
**Competing Interests**<br><br>Use the instructions below to enter a competing interest statement for this submission. On behalf of all authors, disclose any competing interests that could be perceived to bias this work—acknowledging all financial support and any other relevant financial or non-financial competing interests.	The authors have declared that no competing interests exist.

This statement is required for submission and **will appear in the published article** if the submission is accepted. Please make sure it is accurate and that any funding sources listed in your Funding Information later in the submission form are also declared in your Financial Disclosure statement.<br><br>View published research articles from *PLOS ONE* for specific examples.<br><br>**NO authors have competing interests**<br><br>Enter: *The authors have declared that no competing interests exist.*<br><br>**Authors with competing interests**<br><br>Enter competing interest details beginning with this statement:<br><br>*I have read the journal's policy and the authors of this manuscript have the following competing interests: [insert competing interests here]*<br><br>* typeset	
**Ethics Statement**<br><br>Enter an ethics statement for this submission. This statement is required if the study involved:<br><br>• Human participants<br>• Human specimens or tissue<br>• Vertebrate animals or cephalopods<br>• Vertebrate embryos or tissues<br>• Field research<br><br>Write "N/A" if the submission does not require an ethics statement.<br><br>General guidance is provided below. Consult the submission guidelines for detailed instructions. **Make sure that all information entered here is included in the**	Ethical approval for this study was granted by the Research Medical Ethics Commission of the University of Lubumbashi (UNILU/CEM/035/2024)

**Methods section of the manuscript.**<br><br>**Format for specific study types**<br><br>**Human Subject Research (involving human participants and/or tissue)**<br>• Give the name of the institutional review board or ethics committee that approved the study<br>• Include the approval number and/or a statement indicating approval of this research<br>• Indicate the form of consent obtained (written/oral) or the reason that consent was not obtained (e.g. the data were analyzed anonymously)<br><br>**Animal Research (involving vertebrate animals, embryos or tissues)**<br>• Provide the name of the Institutional Animal Care and Use Committee (IACUC) or other relevant ethics board that reviewed the study protocol, and indicate whether they approved this research or granted a formal waiver of ethical approval<br>• Include an approval number if one was obtained<br>• If the study involved *non-human primates*, add *additional details* about animal welfare and steps taken to ameliorate suffering<br>• If anesthesia, euthanasia, or any kind of animal sacrifice is part of the study, include briefly which substances and/or methods were applied<br><br>**Field Research**<br><br>Include the following details if this study involves the collection of plant, animal, or other materials from a natural setting:<br>• Field permit number<br>• Name of the institution or relevant body that granted permission	
**Data Availability**<br><br>Authors are required to make all data underlying the findings described fully available, without restriction, and from the time of publication. PLOS allows rare exceptions to address legal and ethical concerns. See the PLOS Data Policy and FAQ for detailed information.	Yes - all data are fully available without restriction

A Data Availability Statement describing where the data can be found is required at submission. Your answers to this question constitute the Data Availability Statement and **will be published in the article**, if accepted.<br><br>**Important:** Stating 'data available on request from the author' is not sufficient. If your data are only available upon request, select 'No' for the first question and explain your exceptional situation in the text box.<br><br>Do the authors confirm that all data underlying the findings described in their manuscript are fully available without restriction?	
**Describe where the data may be found in full sentences. If you are copying our sample text, replace any instances of XXX with the appropriate details.**<br><br>• If the data are **held or will be held in a public repository**, include URLs, accession numbers or DOIs. If this information will only be available after acceptance, indicate this by ticking the box below. For example: *All XXX files are available from the XXX database (accession number(s) XXX, XXX.*).<br>• If the data are all contained **within the manuscript and/or Supporting Information files**, enter the following: *All relevant data are within the manuscript and its Supporting Information files.*<br>• If neither of these applies but you are able to provide **details of access elsewhere**, with or without limitations, please do so. For example:<br><br>*Data cannot be shared publicly because of [XXX]. Data are available from the XXX Institutional Data Access / Ethics Committee (contact via XXX) for researchers who meet the criteria for access to confidential data.*<br><br>*The data underlying the results*	All relevant data are within the manuscript and its Supporting Information files.

presented in the study are available from (include the name of the third party and contact information or URL).<br>• This text is appropriate if the data are owned by a third party and authors do not have permission to share the data.<br><br>* typeset	
Additional data availability information:	

Powered by Editorial Manager® and ProduXion Manager® from Aries Systems Corporation

Ethnomedicinal Knowledge of plants used in Alternative Medicine as uterotonic in Lubumbashi, Haut-Katanga (DR Congo).

Bashige Chiribagula Valentin [1]*, Okusa Ndjolo Philippe[1], Cedrick Shakalenga Mutombo [2], Manya Mboni Henry[2], Numbi wa Ilunga Evodie [2], Bakari Amuri Salvius[2], Jean Claude Nkwanga[2], Muyumba Singoy Sarah[1], Mwamba Maseho Faustin[2], Biayi Benaja Martin[2], Sangwa Kamulete Gregoire[2], Mbayo Kalubandika Glaubert[2], Muhona Melman[1], Lumbu Simbi Jean Baptiste [3]

[1] Department of Pharmacology, Laboratory of Therapeutic Chemistry and Analysis of Natural Substances, Faculty of Pharmaceutical Sciences (UNILU), 27, avenue Kato, Commune Kampemba, Lubumbashi-DR Congo

[2] Department of Pharmacology, Laboratory of Pharmacognosy, Faculty of Pharmaceutical Sciences, University of Lubumbashi (UNILU) 27, av Kato, Commune Kampemba, Lubumbashi, DR Congo

[3] Department of Chemistry, Faculty of Sciences, University of Lubumbashi (UNILU) N°1 Maternity Avenue, Commune of Lubumbashi, DR Congo.

Corresponding author :

Bashige C. Valentin; bashige.chiribagula@unilu.ac.cd ; Tel.: +243856115389

Author's ORCID

Bashige Chiribagula Valentin (0000-0002-1371-0517), Okusa Ndjolo Philippe (0000-0002-5082-4097), Cedrick Shakalenga Mutombo (0000-0002-3992-5233), Numbi wa Ilunga Evodie (0009-0007-8176-2662), Jean Claude Nkwanga (0009-0003-0828-364X), Mbayo Kalubandika Glaubert (0009-0003-8219-9294), Muhona melman (0009-0006-0938-8373), Biayi Benaja Martin (0009-0003-0760-267X), Muyumba Singoy Sarah (0009-0006-4829-1339)

E-mail address of co-authors.

Bakari Amuri Salvius: bakari.amuri@unilu.ac.cd
Biayi Benaja Martin : Benajab@unilu.ac.cd
Cedrick Shakalenga Mutombo: ShakalengaM@Unilu.ac.cd
Jean Claude Nkwanga : nkwangaj@unilu.ac.cd
Lumbu Simbi Jean Baptiste: lumbujeanbaptiste@gmail.com
Manya Mboni Henry : mbonim@unilu.ac.cd
Mbayo Kalubandika Glaubert : kalubandikam@unilu.ac.cd
Muhona Melman: muhonamelman@gmail.com
Muyumba Singoy Sarah: sarahmuyumba41@gmail.com

Numbi wa Ilunga Evodie : ilungae@unilu.ac.cd
Okusa Ndjolo Philippe: Okusa.Ndjolo@Unilu.ac.cd
Sangwa Kamulete Gregoire : kamulete@unilu.ac.cd

Graphical Abstract

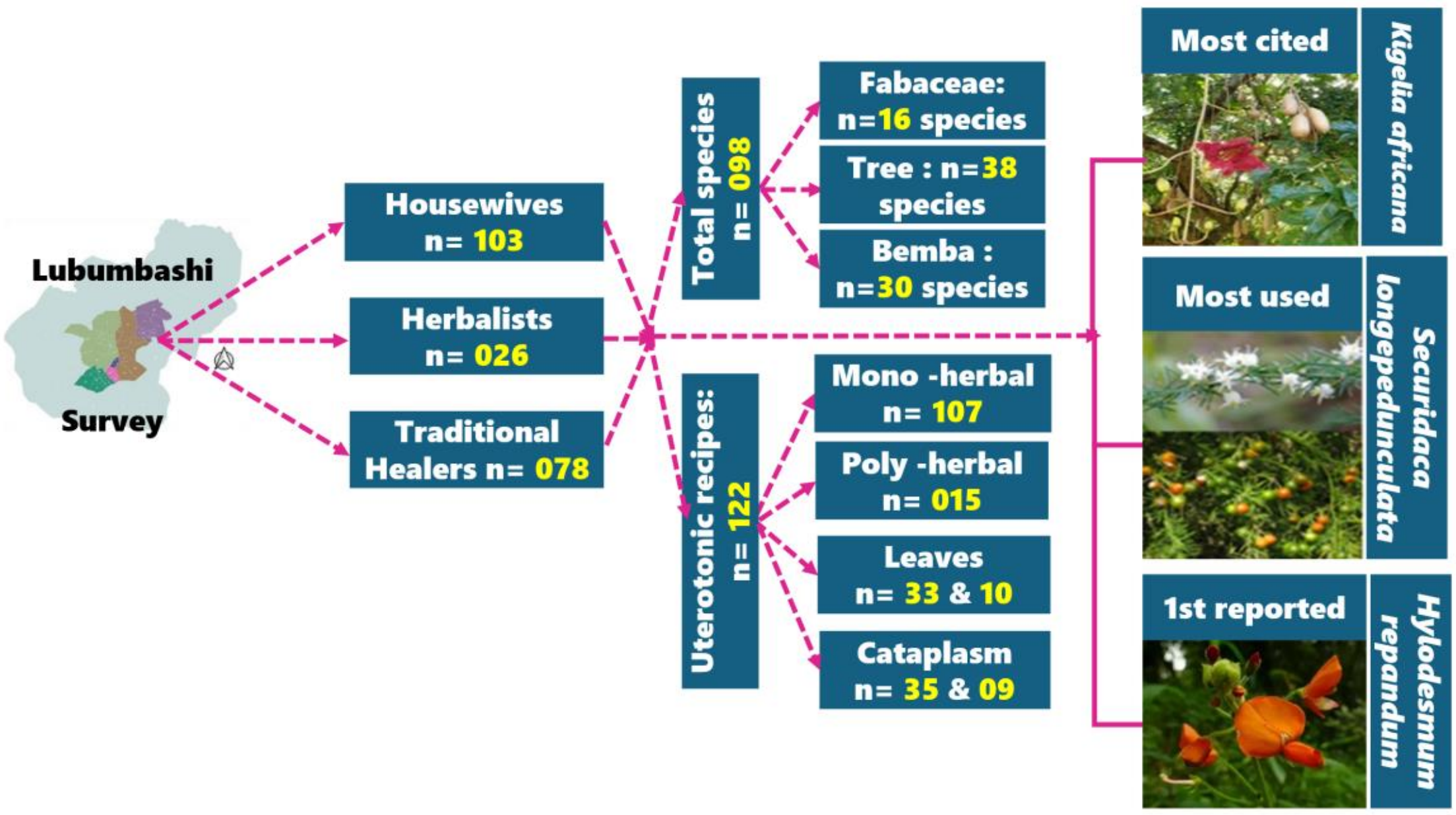

Abstract

Background

Traditional medicine constitutes a principal component of primary healthcare in Democratic Republic of Congo, particularly in Lubumbashi. However, ethnobotanical knowledge related to its use remains incompletely documented and underutilized. The present study aimed to systematically document medicinal plants used as uterotonic among residents of Lubumbashi.

Methods

Ethnobotanical data were collected through structured interviews with 207 informants (housewives, n=103; herbalists, n=26; traditional healers, n=78; sex ratio M/F = 0.5; mean age 35.6 ± 4.3 years; mean professional experience 13 ± 4.2 years).

Results

Informants cited 98 medicinal plant species, predominantly trees (38 species), distributed across 43 botanical families, with Fabaceae being the most represented family (16 species). Malaria was the most frequently reported non-uterotonic indication (46 species). A total of 122 traditional oxytocic formulations were recorded; most involved leaves (43 recipes) and were principally administered as cataplasm (44 recipes). To our knowledge, 34 species were reported for the first time with oxytocic uses, of which eight are documented for the first time as medicinal plants in DRC. Among taxa, *Hylodesmum repandum* (relative citation frequency, RCF = 0.20) and *Crassocephalum montuosum* (RCF = 0.19) exhibited the highest citation frequencies, while *Securidaca longepedunculata* (use value, UV = 0.27) and *Hibiscus sabdariffa* (UV = 0.25) showed the greatest overall use values.

Conclusion

These findings provide a baseline inventory for pharmacological, phytochemical, and toxicological studies, and support prioritization of bioassay-guided validation and conservation strategies for culturally important species.

Keywords: *Kigelia africana, Hylodesmum repandum, Securidaca longepedunculata,* uterotonic plants, Katangese traditional medicine (KTM).

1. Introduction

Childbirth remains a major public health challenge in sub-Saharan Africa and other low- and middle-income countries, including the Democratic Republic of Congo (DRC). Despite advances in modern medicine, maternal and neonatal mortality rates remain high, with a reported rate of 444 maternal deaths per 100,000 births over the past decade [1–3]. The main causes include haemorrhage, hypertensive disorders, infections, unsafe abortions and obstructed labour. While these complications are common due to socioeconomic inequalities, poverty, limited access to healthcare facilities and a shortage of qualified staff, particularly in rural areas [4,5], but they can be prevented with rapid access to quality care [5].

Uterotonics are substances that stimulate myometrial receptors, thereby increasing uterine tone and contractions [6]. These include oxytocin, Carbetocin, misoprostol, ergot and prostaglandin derivatives [7]. Concurrently, pregnant women also utilize natural substances to facilitate childbirth [8,9]reflecting their reliance on traditional medicine due to the constraints imposed by modern medical options.

In addition, conventional medicine has advanced obstetric care through technologies such as prenatal imaging, electronic fetal monitoring, and artificial intelligence algorithms that support conventional uterotonics [10,11]. These advances are inaccessible to a significant proportion of the African population, particularly in DRC, where human and material resources are severely limited [12].

In DRC, traditional medicine plays a central role in childbirth management using plants with uterotonic properties. A review of the literature from 1980 to 2010 identified 280 African oxytocic plants [13], while another reported 16 plants for which experimental uterotonic activity [14]; *i.e.*, *Heimia salicifolia* Link, Lythraceae [15], the roots of *Azanza garckeana* (F. Hoffm.) Exell & Hillc., a synonym for *Thespesia garckeana* F. Hoffm, Malvaceae [16], as welle as the stem bark of *Viburnum opulus* L.,Viburnaceae [17].

In the city of Lubumbashi, to the best of our knowledge, there has been no recorded study systematically documenting uterotonic plants. However, an ethnobotanical investigation conducted by Nama Mwengu (2021) within the Rwashi commune of Lubumbashi documented six plant species traditionally employed for their uterine effects: *Phyllanthus muellerianus* (Kuntze) Exell, *Hibiscus hiernianus* Exell & Mendonça ~~(syn. *Hibiscus gilletii* subsp. hiernianus)~~ , *Acacia macrocentra* Walp ~~(Syn. *Mimosa arenosa var. arenosa*)~~, *Pseudolachnostylis maprouneifolia* Pax, *Strychnos innocua* Delile, and *Annona senegalensis* Pers [18]. Complementarily, a subsequent survey undertaken in Kipushi in 2017, a locality proximal to Lubumbashi and constituting the present experimental context, identified eleven taxa reputed to facilitate parturition. Of these, six species: *Strychnos cocculoides* Baker, *Uapaca kirkiana* Müll.Arg, *Phyllanthus muellerianus*, *Ficus capensis* Thunb ~~(Syn. *Ficus sur* Forssk)~~, *Byrsocarpus orientalis* (Baill.) Baill ~~(Syn. *Rourea orientalis* Baill)~~, and *Aframomum alboviolaceum* (Ridl.) K.Schum , were specifically cited for their putative uterotonic properties [19]. This study aims to invent medicinal plants and recipes with uterotonic effects, used in Lubumbashi's traditional medicine to facilitate childbirth.

2. Material and Methods

2.1. Study area.

The study was conducted in the seven communes of Lubumbashi: Annexe, Kampemba, Katuba, Kamalondo, Kenya, Lubumbashi, and Rwashi, in the Haut-Katanga province in the Democratic Republic of Congo (Figure 1).

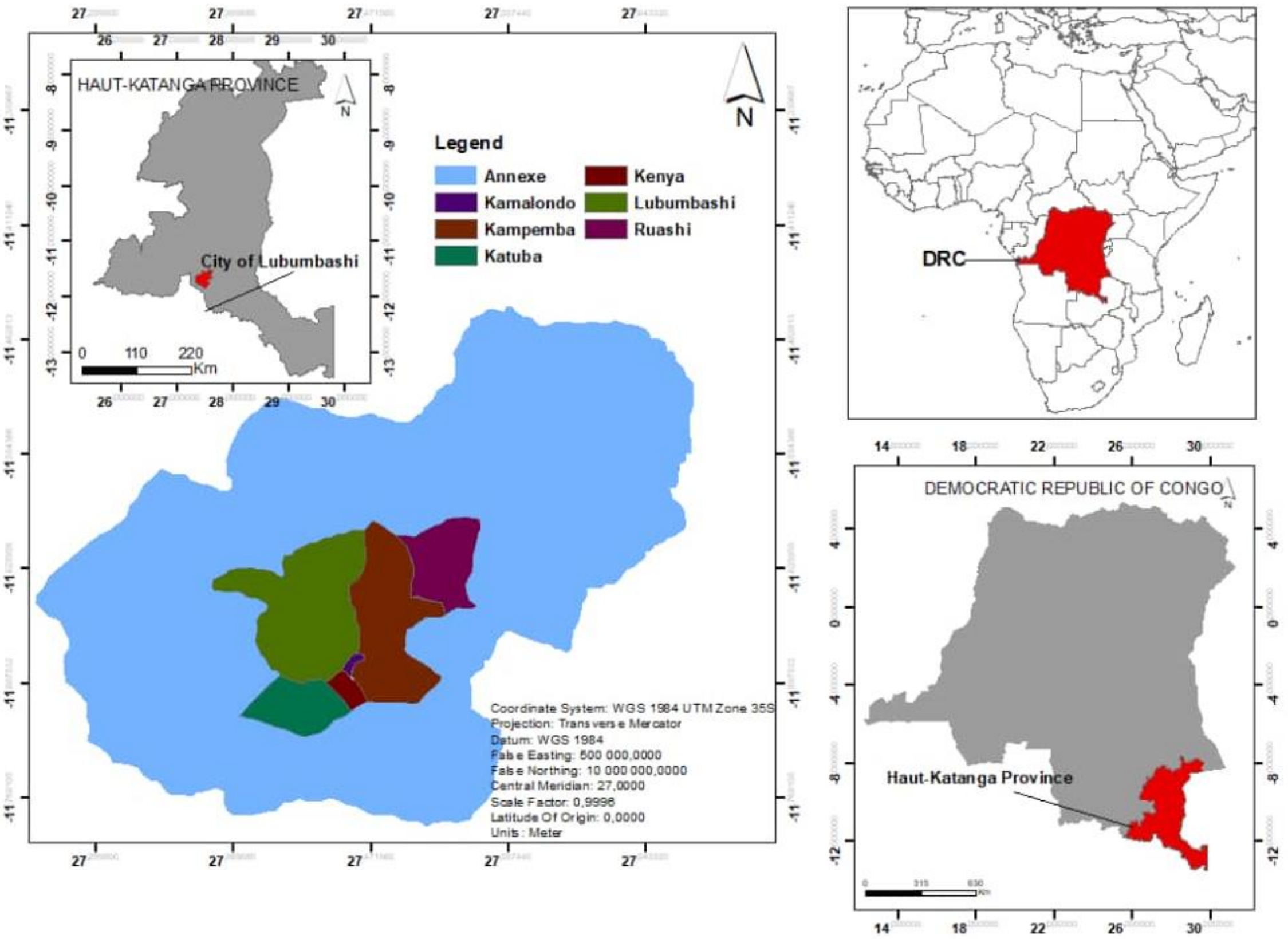

Figure 1 : Lubumbashi, Haut-Katanga province in Democratic Republic of Congo [20].

Lubumbashi is located between 11°26′ and 11°55′ South latitude and 27°15′ and 27°40′ East longitude, at an altitude of approximately 1,230 m above sea level. The region has a tropical climate characterized by a rainy season (November–April) and a dry season (May–October), with a mean annual temperature of 22.4 °C and average annual rainfall of approximately 512 mm. The dominant vegetation type is Miombo woodland, a drought-adapted ecosystem that constitutes an important source of medicinal plant diversity for local communities [21].

2.2 Ethnobotanical data collection.

Between November 2024 and October 2025, a cross-sectional descriptive ethnobotanical survey was conducted among 207 informants, 103 women who had experienced difficult labour and used medicinal plants to facilitate delivery, 26 herbalists, and 78 traditional healers, through direct, open-ended interviews guided by a structured questionnaire (Supplementary Material 1). This survey was pre-tested with 10 women who had experienced difficult childbirth and 5 traditional healers, which helped improve the informants' understanding and compliance.

To determine the minimum sample size to interview among women who have previously experienced difficulties in childbirth, we used probabilistic sampling, given that their number is unknown but estimated to be substantial. This sample size was calculated using the following Cochran formula:

$$n = \frac{z^2 * p * (1-p)}{e^2} \quad (1)$$

(z = 1.96 for 95 % confidence, p = 0.50, e = 0.05), yielding 196 informants. These women were randomly selected from different households in Lubumbashi. Of the 196 women consulted, only 103 (52.5%) reported using plants to accelerate uterine contractions.

The herbalists participating in this study were recruited from six of Lubumbashi's main traditional medicine markets (Mzee, Kenya, Zambia, Kamalondo, Kalala Banza, and Moise), which are dynamic centres of ethnobotanical expertise and living plant traditions. Thirty-six practitioners were interviewed in the various markets. Through direct interviews, they shared the recipes they use, revealing that twenty-six of them (72%) had at least one specific herbal uterotonic formulation.

Traditional healers included in this study were initially selected from a pre-established list of 50 practitioners compiled during a previous survey [21]. This process resulted in the identification of 47 traditional healers, of whom only 45 reported using uterotonic medicinal plants. To increase the sample size, snowball sampling was subsequently employed, resulting in the inclusion of 33 additional healers, one of whom had already appeared on the initial list.

1.3. Data processing and analysis.

Plant specimens were collected in the presence of informants, photographed, pressed, and dried following standard herbarium procedures. Taxonomic identification was performed by qualified botanists at the Kipopo Herbarium (Lubumbashi, DRC). Voucher specimens were deposited under assigned reference numbers. Scientific names were verified and updated using Plants of the World Online (POWO: https://powo.science.kew.org/) and World Flora Online databases (WFO: http://www.worldfloraonline).

To determine the status of each inventoried taxon in relation to the contemporary scientific record, the accepted botanical name of each species was systematically queried in principle bibliographic databases (Google, Google Scholar, PubMed, Scopus). For every species, we documented the presence or absence of: previous ethnobotanical reports of use as a uterotonic; experimentally verified uterotonic activity; documentation of medicinal use within the Democratic Republic of Congo; documentation of medicinal use specifically from Lubumbashi; and documentation of medicinal use from regions outside the DRC. These categorical assessments were employed to characterize the current state of knowledge for each plant.

Given the current controversy surrounding ethnobotanical indexes [22]We have used only two ethnobotanical indices to assess the significant species: the Use Value (UV) and the Relative Citation Frequency (RCF). However, apart from these two ethnobotanical indices, we have expressed the results as percentages (%).

The formula determines the use value (UV):

$$UV = \frac{\text{Number of uses mentioned by an informant for a plant}}{\text{Number of informants who cited the plant}} \quad (2)$$

The Relative citation frequency on the plant (organ) (RCF) was calculated by the formula:

$$RCF = \frac{\text{Number of informant who cited the plant}}{\text{Number of informants who took part in the survey}} \quad (3)$$

In this study, UV: makes it possible to evaluate the medicinal importance of a plant in the study area; RCF is a measure of the popularity of a plant with the people who use it to facilitate childbirth. This also implies consensus among the informants on the use of a plant to facilitate childbirth. This index was also determined by parallelism of form for other reasons for consultation than the facilitation of childbirth and the various recipes listed.

1.4. Ethics considerations

Ethical approval for this study was granted by the Research Medical Ethics Commission of the University of Lubumbashi (UNILU/CEM/035/2024), and we obtained informed verbal consent from all 207 participants after clearly explaining the study's purpose and procedures. Any individual who declined to consent or had no prior experience using medicinal plants to aid childbirth was respectfully excluded. We reassured each informant that their insights would be used solely for academic research, that personal identifiers would be removed, and that no personal information would be shared with third parties.

3. Results

3.1. Profile of inventoried medicinal plants.

A total of 98 plant species were identified as being used as uterotonic agents in traditional medicine in Lubumbashi, following ethnobotanical surveys of traditional practitioners, herbalists, and housewives. Of these species, 61 were reported by traditional practitioners (35 of which were also mentioned by the other categories of informants), 35 by herbalists (24 of which were mentioned by the other categories of informants), and 28 by housewives (19 of which were mentioned by the other categories of informants). The general characteristics of these plants and their status as prior knowledge (bibliographic) in relation to uterotonic use are shown in Table 1.

Table 1: **General information of plants surveyed**

**Species (Family, HC)**	**Appellation (Ethnicity)**[GT, MT]	**N° Recipe (n=107)**[UP,P]	N_C	**RCF (n=207)**	N_u	**UV (n=60)**	**Non uterotonic indication**	**Ref.**
Abrus precatorius L (Fabaceae,)	Buhiti (Lamba) [h,p]	**R1** [w,ε]	9	0.04	9	0.15	Bronchitis, jaundice, hepatic diseases, contraception, tumours, sexual dysfunction, malaria, and snake bites	† : [23,24] ‡ : [24,25] ¥ : [26]
Adenia gummifera (Harv.) Harms (Passifloraceae)	Kimboyi (Lala) [k,l]	**R2** [α,μ]	9	0.04	8	0.13	Infertility, sexually transmitted infections, gastrointestinal diseases, leprosy, respiratory infections, malaria and menstrual problems	† : NR ‡ : NR ¥ : [26]

Species (Family, HC)	Appellation (Ethnicity)[GT, MT]	Nº Recipe (n=107)[UP,P]	N_C	RCF (n=207)	N_u	UV (n=60)	Non uterotonic indication	Ref.
Aerva javanica (Burm.f.) Juss. Ex Schult. (Amaranthaceae, KIP000000601)	Kapoka (Bemba) [c,q]	**R3** [x,δ]	8	0.04	9	0.15	Cancer, wound,helminth, atherosclerosis , diabetes, hyperlipidemia, fungal infection, and hepatic diseases	† : NR ‡ : [27] ¥ : [28] Ŧ : [28,29]
Aframomum alboviolaceum (Ridl.) K.Schum. (Zingiberaceae KIP000001433)	Ntundulu (Kikongo) [f,o]	**R4** [α,δ]	9	0.04	14	0.23	Malaria, helminth, fever, Dysmenorrhea, snake bites, headache, cough, gastritis, amoebic dysentery, haemorrhoids, myoma, and pruritis.	† : NR ‡ : NR Ŧ: [30]
		**R5** [z,ε]						
Afrothismia mhoroana Cheek [Syn *Rauvolfia caffra* L] (Burmanniaceae, KIP000000602)	Mutalala (Bemba)[b,o]	**R6** [α,δ]	12	0.06	4	0.07	Fever, headaches, and gastrointestinal diseases	† : NR ‡ : NR Ŧ : [31]
Ageratum conyzoides L. (Asteraceae, KIP000111014)	Uratulu (Kikongo) [d,l]	**R7** [x,δ]	28	0.14	4	0.07	Wounds, skin infections, and respiratory ailments	† : NR ‡ : [32] Ŧ : [20]
Albizia adianthifolia (Schumach.) W.Wight (Fabaceae, KIP000001031)	Kapeta nzovu (Bemba) [f,r]	**R8** [β,ψ]	29	0.14	11	0.18	Diabetes, eye problems, gastrointestinal diseases, haemorrhoids, headache, neurodegenerative disorders, respiratory ailments, wounds, skin diseases, sexually transmitted infections	† : NR ‡ : NR Ŧ: [20]
Albizia lebbeck (L.) Benth (Fabaceae, KIP000111016)	Siriza (Lamba) [d,r]	**R9** [w, ε]	11	0.05	11	0.18	Fever, diarrhoea, dysentery, asthma, cold, cough, conjunctivitis, arthritis, dental problems and skin disease	† : NR ‡ : NR ¥ : [33]
		**R10** [w, λ]						
Alchornea floribunda Müll.Arg (Euphorbiaceae, KIP000000603)	Lubumbidi (Kiyombe) [f,p]	**R11** [γ,μ]	17	0.08	3	0.05	Inflammatory diseases, fungal ailments, and diarrhoea	† : NR ‡ : NR ¥: [34]
Allium sativum L (Amaryllidaceae)	Sabolo (Sanga) [d,m]	**R12** [u,δ]	12	0.06	9	0.15	Fungal ailments, cancer, thrombosis, cardiovascular diseases, diabetes, viral diseases and parasitic diseases	† : [35] ‡ : [36] Ŧ : [37]
Aloe chabaudii Schönland (Asphodelaceae, KIP000000604)	Kilambwe ne Byana (Bemba) [f,n]	**R13** [γ, δ]	17	0.08	8	0.13	Skin diseases, Inflammatory diseases, respiratory ailments, urinary diseases,	† : NR ‡ : NR Ŧ : [38,39]

Species (Family, HC)	Appellation (Ethnicity)[GT, MT]	Nº Recipe (n=107)[UP,P]	N_C	RCF (n=207)	N_u	UV (n=60)	Non uterotonic indication	Ref.
							Poisoning, snake bites, cardiovascular diseases	
Aloe vera (L.) Burm.f (Asphodelaceae, KIP000000605)	Cizimyamuliro (Shi) [d,n]	**R14** [γ,δ]	15	0.07	11	0.18	Skin diseases, Inflammatory diseases, respiratory ailments, urinary diseases, Poisoning, snake bites, cardiovascular diseases, wounds, diabetes, sexually transmitted infections	† : [40]<br>‡ : [41]<br>₸ : [42]
Anchusa ovata Lehm (Boraginaceae, KIP000000606)	Kandasole (Sanga) [j,l]	**R15** [α,μ]	12	0.06	6	0.10	Ulcer, cough, fever, wounds, skin diseases	† : NR<br>‡ : NR<br>₮: [43]
		**R16** [α, δ]						
Annona senegalensis Pers (Annonaceae, KIP452120003)	Mulolo (Bemba) [i,r]	**R17** [y, ε]	7	0.03	7	0.12	Prostate cancers, respiratory diseases, poisoning, rheumatic pains, infertility care and syphilis	† : NR<br>‡ : [32]<br>₸ : [42]
Antidesma venosum E.Mey. ex Tul (Phyllanthaceae, KIP293612638)	Kifubia (Luba-Kat) [h,p]	**R18** [β, ε]	35	0.17	7	0.12	Typhoid, gastrointestinal diseases, diabetes, pneumonia, wounds, and sexually transmitted infections	† : NR<br>‡ : NR<br>₸ : [20]
Asparagus africanus Lam. (Asparagaceae, KIP000111026)	Atangwali (Hemba) [h,p]	**R19** [γ, ε]	5	0.02	17	0.28	Malaria, helminth, fever, Dysmenorrhea, headache, cough, gastritis, amoebic dysentery, stomach pain, ulcers, haemorrhoids, myoma, syphilis, gonorrhoea, and pruritis.	† : [44]<br>‡ : [45,46]<br>₮ : [47]
Azadirachta indica A. Juss. (Meliaceae, KIP000000607)	Nfwama (Sabwa) [d,r]	**R20** [γ, ε]	4	0.02	13	0.22	Fever, malaria, wounds, headache, ulcers, respiratory diseases, cancer, diabetes, leprosy, dengue fever, chickenpox, and skin diseases	† : [40]<br>‡ : [27]<br>₸ : [42]
		**R21** [z, λ]						
Baphiopsis parviflora Benth. ex Baker (Fabaceae, KIP000000608)	Waase (Tabwa) [a,r]	**R22** [β, ε]	5	0.02	9	0.15	Wounds, coughs, hypertension, erectile dysfunction, skin infections, urinary tract infections, respiratory ailments, and gastro-intestinal diseases.	† : NR<br>‡ : NR<br>¥ : [48,49]

Species (Family, HC)	Appellation (Ethnicity)[GT, MT]	Nº Recipe (n=107)[UP,P]	N_C	RCF (n=207)	N_u	UV (n=60)	Non uterotonic indication	Ref.
Bidens pilosa L (Asteraceae, KIP000000609)	Mbamba ngoma (Luba-Kat) [d,l]	**R23** [γ, θ]	5	0.02	6	0.10	Intoxication, hypertension, dysentery, hepatic diseases, ulcers	† : [40,50] ‡ : [51] Ŧ : [30]
Bobgunnia madagascariensis (Desv.) J.H.Kirkbr. & Wiersema (Fabaceae, KIP000000610)	Munienze (Luba-Kat) [f,r]	**R24** [β, ε]	6	0.03	9	0.15	Bilharzia, leprosy, cataract, dysentery, headache, cough, diarrhea, and sexually transmitted infections	† : NR ‡ : NR Ŧ : [30]
Boerhavia diffusa L (Nyctaginaceae, KIP000000611)	Alini (Bemba) [h,l]	**R25** [γ, δ]	5	0.02	8	0.13	Cough, hypertension, malaria, fever, diabetes, wound, gastro-intestinal diseases	† : NR ‡ : NR ¥ : [52]
Bombax ceiba L. (Malvaceae, KIP000000612)	Mulul ilav (Rund) [d,r]	**R26** [γ, ε]	5	0.02	6	0.10	Cholera, coughs, urinary tract infections, dysentery, and gastro-intestinal diseases	† : NR ‡ : NR Ŧ : [53]
Bothriocline longipes (Oliv. & Hiern) N.E.Br. (Asteraceae, KIP000000613)	Lwibaye (Shi) [f,q]	**R27** [γ,θ]	6	0.03	14	0.23	Fever, malaria, wound, headache, ulcers, respiratory diseases, cancer, diabetes, leprosy, dengue fever, chickenpox, and skin diseases, stomach	† : [54] ‡ : [25] Ŧ : [55]
		**R28** [α,δ]						
Cajanus cajan (L.) Huth (Fabaceae, KIP000000614)	Golyolyo (Luba-Kat) [d,n]	**R29** [z, ε]	7	0.03	11	0.18	Diabetes, malaria, ulcers, gastro-intestinal diseases, haemorrhoids, headache, respiratory ailments, wounds, skin diseases, sexually transmitted infections	† : [56] ‡ : [57] Ŧ : [37]
Capsicum annuum L. (Solanaceae, KIP000000615)	Pilipili (Swahili) [d,l]	**R30** [z, δ]	12	0.06	6	0.10	Cancers, wounds, fever, malaria, stomach	† : NR ‡ : [32] Ŧ : [58]
Cassia sieberiana DC (Fabaceae, KIP000000616)	Kafunga nanshi (Bemba) [f,r]	**R31** y, ε	9	0.04	8	0.13	Fever, indigestion, constipation, Drepanocytosis, diabetes, malaria, and skin diseases	† : NR ‡ : NR ¥ : [26]
Cissampelos mucronata A.Rich. (Menispermaceae, KIP000000617)	Mukoko (Luba-Kat) [h,n]	**R32** [γ, δ]	7	0.03	6	0.10	Diarrhea, dysentery, sexually transmitted infections, infertility, fever	† : [59] ‡ : [57] Ŧ : [60,61]
Cissus schmitzii Dewit (Vitaceae, KIP000000618)	Lenda (Luba-Kat) [a,p]	**R33** [α, ε]	9	0.04	7	0.12	Wounds, ulcers, fever, respiratory ailments, sexual dysfunction, diabetes	† : NR ‡ : NR T̄ : [62]

Species (Family, HC)	Appellation (Ethnicity)[GT, MT]	Nº Recipe (n=107)[UP,P]	N_C	RCF (n=207)	N_u	UV (n=60)	Non uterotonic indication	Ref.
Clausena anisata (Willd.) Hook.f. ex Benth (Rutaceae, KIP000000619)	Umusanga (Bemba) [f,r]	**R34** [γ, ε]	10	0.05	5	0.08	Hypertension, respiratory ailments, fever, stomach	† : NR ‡ : [63] Ŧ : [63]
Clematis scabiosifolia DC [Syn *Clematis villosa* subsp. Villosa] (Ranunculaceae, KIP000000620)	Karhula (Shi) [a,n]	**R35** [γ, θ]	11	0.05	7	0.12	Cancer, Pain, Inflammatory diseases, headaches, skin diseases, sexually transmitted infections	† : NR ‡ : [64] T̄ : [64]
Cochlospermum planchonii Hook.f. ex Planch (Bixaceae, KIP000000621)	Sowaga (Tabwa) [f,q]	**R36** [γ, θ]	15	0.07	6	0.10	Malaria, diarrhoea, hepatic diseases, Inflammatory diseases, stomach	† : NR ‡ : NR Ŧ : [65,66]
Combretum molle R.Br. ex G.Don (Combretaceae, KIP000000622)	Mulema (Bemba) [f,p]	**R37** [γ, μ]	14	0.07	8	0.13	Drepanocytosis, diarrhoea, helminth, wound, ulcers Poisoning, headaches	† : NR ‡ : NR T̄ : [37]
Commelina africana L (Commelinaceae, KIP000000623)	Idangabani (Bemba) [i,n]	**R38** [γ, ε]	13	0.06	6	0.10	Drepanocytosis, urinary tract infections, diarrhoea, haemorrhoids, stomach	† : [40] ‡ : [67] ¥ : [68]
Crassocephalum montuosum (S.Moore) Milne-Redh. (Asteraceae, KIP437629969)	Cifula (Shi) [i,l]	**R39** [γ, δ]	39	0.19	14	0.23	Fever, malaria, wound, headache, ulcers, respiratory diseases, cancer, diabetes, leprosy, rheumatism, dengue fever, chickenpox, and skin diseases	† : NR ‡ : NR T̄ : [69]
		**R40** [γ,δ]						
Crassocephalum vitellinum (Benth.) S.Moore (Asteraceae, KIP000000624)	Shungululu (Shi) [f,l]	**R41** [γ, δ]	47	0.23	7	0.12	Fever, malaria, sexually transmitted infections, wounds, gastro-intestinal diseases, stomach	† : NR ‡ : [25] ¥: [64]
Cucurbita pepo L (Cucurbitaceae, KIP000000625)	Liboga (Swahili) [d,l]	**R42** [z, δ]	11	0.05	7	0.12	hepatic diseases, diabetes, cancer, ulcer, wound, stomach	† : NR ‡ : [70] ¥ : [71]
Cyanthillium cinereum (L.) H.Rob. [syn *Vernonia cinerea* (L.) Less] (Asteraceae, KIP000000626)	Musubgu bululu (Luba-kas) [g,n]	**R43** [γ, δ]	13	0.06	5	0.08	Urinary tract infections, diarrhoea, cough, respiratory diseases, stomach	† : NR ‡ : NR T̄ : [37]
Cymbopogon citratus (DC.) Stapf (Poaceae, KIP000000627)	Masela mputu (Luba-Kas) [d,n]	**R44** [α, ε]	11	0.05	8	0.13	Fever, stomach, headache, malaria, inflammatory diseases, cough, Amoebae	† : [72] ‡ : [32] T̄ : [20,21]

Species (Family, HC)	Appellation (Ethnicity)[GT, MT]	Nº Recipe (n=107)[UP,P]	N_C	RCF (n=207)	N_u	UV (n=60)	Non uterotonic indication	Ref.
Cynodon dactylon (L.) Pers. (Poaceae, KIP000000628)	Lukaka (Sanga) [e,l]	**R45** [x, δ]	12	0.06	6	0.10	Inflammatory diseases, fungal ailments, hypertension and diarrhoea, wound	† : NR ‡ : [73] ₸ : [74]
Dalbergia boehmii Taub (Fabaceae, KIP000000629)	Katambo (Zela) [f,q]	**R46** [β, δ]	12	0.06	7	0.12	Diabetes, urinary tract infections, skin diseases, pain, Diarrhea, fever	† : NR ‡ : [73] ₸ : [37]
Diplorhynchus condylocarpon (Müll.Arg.) Pichon [Syn *Diplorhynchus mossambicensis* Benth] (Apocynaceae, KIP361753009)	Mwenge (Sanga) [f,p]	**R47** [y, δ]	14	0.07	6	0.10	Headache, snake bites, hepatic diseases, respiratory diseases, gastrointestinal diseases	† : NR ‡ : [75] ₸ : [37,62]
Ekebergia capensis Sparrm (Meliaceae, KIP000000630)	Nyamaru (Bemba) [h,r]	**R48** [z, δ]	13	0.06	9	0.15	Fever, malaria, stomach, skin diseases, wounds, cough, headache	† : [76] ‡ : [14] ₸: [77]
Elaeis guineensis Jacq (Arecaceae, KIP000000631)	Ekaci (Bembe) [f,r]	**R49** [β, δ]	9	0.04	6	0.10	headaches, pains, rheumatism, cardiovascular diseases, wounds	† : NR ‡ : [78] ₸ : [37]
Eleusine indica (L.) Gaertn (Poaceae, KIP000000632)	Palasi (Zela) [i,l]	**R50** [y, θ]	4	0.02	6	0.10	asthma, diarrhoea, stomach, dysentery, respiratory diseases	† : NR ‡ : [51] ₸ : [37]
Erythrina abyssinica Lam (Fabaceae, KIP000000633)	Kisungwa (Bemba) [f,r]	**R51** [y, µ]	5	0.02	12	0.20	Tuberculosis, malaria, HIV/AIDS, diarrhoea, cancer, meningitis, inflammatory diseases, urinary tract infections, wounds, diabetes, and skin diseases.	† : NR ‡ : [57] ₸ : [37]
Euphorbia hirta L. (Euphorbiaceae, KIP000000634)	Butonvitonvi (Bemba) [f,l]	**R52** [x, ε]	13	0.06	5	0.08	Cancer, Asthma, wounds, gastrointestinal diseases	† : NR ‡ : [32,57] ₸ : [20,37]
Ficus capensis Thunb [*Ficus sur* Forssk] (Moraceae, KIP539378804)	Mukiluakilua (Bemba) [h,r]	**R53** [y, δ]	37	0.18	8	0.13	Drepanocytosis, diarrhoea, fever, cough, ulcers, skin diseases, stomach	† : NR ‡ : [32] ₸: [37]
Ficus stuhlmannii Warb (Moraceae, KIP000111046)	Mupulampaka (Bemba) [f,r]	**R54** [y, δ]	39	0.19	6	0.10	Drepanocytosis, wounds, haemorrhoids, diabetes, hepatic diseases	† : NR ‡ : NR ₸ : [20,62]
Flueggea virosa (Roxb. ex Willd.) Royle	Kasansubwanga (Bemba) [f,p]	**R55** [y, ε]	12	0.06	5	0.08	Fever, gastrointestinal diseases, skin diseases, stomach	† : NR ‡ : NR ₸ : [37]

Species (Family, HC)	Appellation (Ethnicity)[GT, MT]	Nº Recipe (n=107)[UP,P]	N_C	RCF (n=207)	N_u	UV (n=60)	Non uterotonic indication	Ref.
(Phyllanthaceae, KIP000000635)								
Grona adscendens (Sw.) H.Ohashi & K.Ohashi (Syn *Desmodium adscendens* (Sw.) DC] (Fabaceae, KIP000000636)	Busundu (Shi) [f,n]	**R56** [γ, δ]	17	0.08	5	0.08	asthma, diarrhoea, stomach, dysentery, respiratory diseases	† : NR ‡ : NR Ŧ: [79,80]
Gymnanthemum amygdalinum (Delile) Sch.Bip. [Syn *Vernonia amygdalina* Delile] (Asteraceae, KIP312286606)	Mubirizi (Shi) [f,p]	**R57** [β, ε]	20	0.10	9	0.15	fever, malaria, diarrhoea, cough, dysentery, headache, stomach pains, and hepatic diseases	† : [40,67,81] ‡ : [57] Ͳ : [37]
Harungana madagascariensis Lam. Ex Poir (Hypericaceae, KIP000000637)	Kafifi (Bemba) [f,r]	**R58** [γ,ε]	8	0.04	6	0.10	Drepanocytosis, malaria, gastrointestinal diseases, diabetes, hepatic diseases	† : NR ‡ : NR Ͳ : [37]
Hexalobus monopetalus (A.Rich.) Engl. & Diels (Annonaceae, KIP000000638)	Nkoyo (Luba-kat) [f,r]	**R59** [α,μ]	19	0.09	5	0.08	Fever, headache, gastrointestinal diseases, stomach	† : NR ‡ : NR Ͳ : [62]
Hibiscus sabdariffa L (Malvaceae, KIP000111148)	Mulenda (Swahili) [f,l]	**R60** [γ,θ]	8	0.04	15	0.25	Cancers, wounds, fever, malaria, stomach pain, Cardiovascular diseases, diabetes, viral diseases, parasitic diseases, gastrointestinal diseases, diabetes, hyperlipidemia, and hypertension.	† : [82] ‡ : [27] Ͳ : [83]
Hylodesmum repandum (Vahl) H.Ohashi & R.R.Mill [*Desmodium repandum* (Vahl) Poir.] (Fabaceae, KIP000000638)	Irhuza (Shi) [f,n]	**R61** [β,ε]	41	0.20	6	0.10	Fever, ulcers, gastrointestinal diseases, malaria, stomach	† : NR ‡ : NR ¥ : [84]
Hymenocardia acida Tul (Phyllanthaceae, KIP464414491)	Kapempe (Shi) [f,r]	**R62** [β,μ]	11	0.05	5	0.08	Malaria, skin diseases, haemorrhoids, Parasites diseases, parasitic diseases, and diabetes	† : NR ‡ : NR Ͳ : [85]
Juglans nigra L (Juglandaceae, KIP000000639)	Nusumbau (Bemba) [d,r]	**R63** [z,μ]	12	0.06	5	0.08	Parasitic diseases, gastrointestinal diseases, diabetes, ulcers	† : NR ‡ : NR Ͳ : [86]

Species (Family, HC)	Appellation (Ethnicity)[GT, MT]	Nº Recipe (n=107)[UP,P]	N_C	RCF (n=207)	N_u	UV (n=60)	Non uterotonic indication	Ref.
Khaya nyasica Stapf ex Baker f (Meliaceae, KIP000001372)	Mbamba (Luba-kat) [b,r]	**R64** [y, ε]	36	0.17	8	0.13	Cancers, diabetes, Drepanocytosis, ulcers, hepatic diseases, gastrointestinal diseases	† : NR ‡ : NR ₸ : [20,37]
Kigelia aethiopum (Fenzl) Dandy [*Kigelia africana* subsp. Africana] (Bignoniaceae, KIP000001376)	Kivungwe (Tabwa) [f,r]	**R65** [y, ε]	27	0.13	7	0,12	Wounds, fever, gastrointestinal diseases, skin diseases, respiratory diseases, cancers	† : NR ‡ : NR ₸ : [86]
Kigelia africana (Lam.) Benth (Bignoniaceae, KIP000000640)	Kivungwila (Luba-kat) [f,r]	**R66** [β,θ]	51	0.25	10	0.17	Malaria, Wounds, fever, gastrointestinal diseases, skin diseases, respiratory diseases, cancers, ulcers, urinary tract infection	† : NR ‡ : [32] ₸ : [86]
Leonotis nepetifolia (L.) R.Br (Lamiaceae, KIP000000641)	Kacumucumu (Shi) [g,l]	**R67** [γ, θ]	18	0.09	10	0.17	Fever, cough, malaria, rheumatism, hypertension, gastro-intestinal diseases, skin diseases, respiratory diseases, pain	† : NR ‡ : NR ¥ : [87]
Leucas martinicensis (Jacq.) R.Br. (Lamiaceae, KIP000000642)	Kanyamafundo (Shi) [f,l]	**R68** [ν, θ]	6	0.03	9	0.15	kidney disorders, rheumatism, cough, diarrhea, fevers, gastro-intestinal diseases, skin diseases, neurodegenerative disorders	† : NR ‡ : [57] ₸ : [88]
Luffa cylindrica (L.) M.Roem [*Cucumis melo* L] (Cucurbitaceae, KIP000111051)	Kikenda mbayo (Luba-kat) [f,l]	**R69** [z, μ]	17	0.08	11	0.18	Respiratory diseases, urinary tract infection, ulcers, Parasites diseases, hypertension, hyperlipidemia, inflammatory diseases, viral diseases, diabetes, wounds	† : [40,89] ‡ : [57] ¥ : [55]
Malus domestica (Suckow) Borkh (Rosaceae, KIP000000643)	Chungwa nuni (Swahili) [d,r]	**R70** [z,ε]	6	0.03	6	0.10	hypertension, diabetes, hepatic diseases, diarrhoea, fevers	† : NR ‡ : [51] ₸ : [42]
Millettia griffoniana Baill. (Fabaceae, KIP000000644)	Umuzimbe (Shi) [f,r]	**R71** [β, ε]	16	0.08	4	0.07	Cancers, viral diseases, Inflammatory diseases	† : [90] ‡ : [91] Ŧ : [92]
Ochna schweinfurthiana F.Hoffm. (Ochnaceae, KIP000000645)	Ikyoni (Bemba) [f,r]	**R72** [β, ε]	7	0.03	8	0.13	Drepanocytosis, helminth, typhoid fever, malaria, skin diseases, respiratory diseases, wounds	† : NR ‡ : [93,94] ₸ : [94]
Ocimum americanum L	Lueni (Bemba) [i,l]	**R73** [x, μ]	15	0.07	5	0.08	Wound, cough, malaria, cough	† : NR ‡ : [32]

Species (Family, HC)	Appellation (Ethnicity)GT, MT	Nº Recipe (n=107)UP,P	N_C	RCF (n=207)	N_u	UV (n=60)	Non uterotonic indication	Ref.
(Lamiaceae, KIP000000646)								₸ : [20]
Paramollugo nudicaulis (Lam.) Thulin (Molluginaceae, KIP000000647)	Eswani (Tabwa) [i,l]	**R74** [x, µ]	9	0.04	5	0.08	Coughs, fever, respiratory diseases, wounds	† : NR ‡ : NR ₸ : [95]
Parinari curatellifolia Planch. ex Benth. (Chrysobalanaceae, KIP000001385)	Mupundu (Luba-kat) [f,r]	**R75** [β, δ]	14	0.07	4	0.07	Diabetes, cancer, inflammatory diseases, malaria	† : NR ‡ : NR ₸ : [62]
*Pentaclethra macrophyll*a Benth. (Fabaceae, KIP000111062)	Bubala (Kirega) [f,r]	**R76** [y, ε]	11	0.05	9	0.15	Drepanocytosis, wounds, haemorrhoids, diabetes, hepatic diseases, helminth, diarrhoea, hyperlipidemia	† : [96] ‡ : [97] [32] ₸ : [98]
Phyllanthus amarus Schum. & Thonn. (Phyllanthaceae, KIP000111065)	Kavudi (Luba-kat) [d,l]	**R77** [γ, ε]	13	0.06	6	0.10	Wounds, stomach diseases, urinary tract infection, hepatic diseases, gastrointestinal diseases	† : NR ‡ : [32] ₸ : [20]
Phyllanthus microdendron Müll.Arg. (Phyllanthaceae, KIP000000648)	Unono (Luba-kat) [f,r]	**R78** [γ, ε]	12	0.06	10	0.17	Wounds, stomach diseases, urinary tract infection, hepatic diseases, gastro-intestinal diseases, diabetes, viral diseases, cancers, malaria	† : NR ‡ : NR ₸ : [99]
Phyllanthus muellerianus (Kuntze) Exell (Phyllanthaceae, KIP277202454)	Modere (Sanga) [f,p]	**R79** [α, ε]	7	0.03	11	0.18	Wounds, stomach diseases, urinary tract infection, hepatic diseases, gastro-intestinal diseases, diabetes, viral diseases, cancers, malaria, hypertension	† : NR ‡ : [100] ₸ : [30,62]
Phyllanthus ovalifolius Forssk [Syn *Phyllanthus guineensis*] (Phyllanthaceae, KIP000000649)	Mupetualupe (Bemba) [f,p]	**R80** [γ, δ]	6	0.03	11	0.18	Wounds, stomach diseases, urinary tract infection, hepatic diseases, gastro-intestinal diseases, diabetes, viral diseases, cancers, malaria, hypertension	† : NR ‡ : [25] ₸ : [37,62]
		**R81** [z,δ]						
Physalis peruviana L (Solanaceae, KIP000000650)	Imbuhu (Shi) [d,n]	**R82** [z,ε]	5	0.02	10	0.17	Cancer, diabetes, ulcers, malaria, asthma, rheumatism, gastro-intestinal diseases, pain, fever.	† : NR ‡ : [57] ¥ : [26,101]

Species (Family, HC)	Appellation (Ethnicity)[GT, MT]	Nº Recipe (n=107)[UP,P]	N_C	RCF (n=207)	N_u	UV (n=60)	Non uterotonic indication	Ref.
Piliostigma thonningii (Schum.) Millne-Redhead (Fabaceae, KIP000000651)	Kifumbe (Bemba) [f,r]	**R83** [γ,δ]	3	0.01	9	0.15	Coughs, fever, respiratory diseases, wounds, sexually transmitted infections, malaria, typhoid fever, haemorrhoids	† : NR ‡ : [32] ₸ : [20,21]
Plumbago zeylanica L (Plumbaginaceae, KIP000111067)	Daunani (Zela) [i,p]	**R84** [γ,δ]	4	0.02	6	0.10	Haemorrhoids, inflammatory diseases, diabetes, hypertension, Drepanocytosis	† : [102][103] ‡ : [25,103] ¥ : [104]
Psidium guajava L. (Myrtaceae, KIP000301861)	Ipera (Shi) [f,r]	**R85** [β, δ]	7	0.03	12	0.20	Ulcer, cough, fever, wounds, skin diseases, malaria, hepatic diseases, helminth, diarrhoea, hyperlipidemia, fungal diseases	† : [105] ‡ : [32] ₸ : [37]
Ricinus communis L (Euphorbiaceae, KIP000000652)	Mubalika (Bemba) [b,p]	**R86** [w, ε]	9	0.04	5	0.08	bilharziasis, gastro-intestinal diseases, hepatic diseases, helminth	† : [106] ‡ : [27] ₸ : [86]
Securidaca longepedunculata Fresen (Polygalaceae, KIP000000653)	Lupapi (Bemba) [f,r]	**R87** [β, ε]	8	0.04	16	0.27	sexually transmitted infections, malaria, typhoid fever, haemorrhoids, coughs, epilepsy, gastro-intestinal diseases, headaches, skin diseases, hyperlipidemia, sexually transmitted infections, rheumatism, aphrodisiac, epilepsy, tuberculosis, ulcers, stomach pain	† : NR ‡ : [107] ₸ : [37,62]
Senegalia polyacantha (Willd.) Seigler & Ebinger [Syn *Acacia polyacantha* Willd] (Fabaceae, KIP000000654)	Kibombolo (Tabwa) [h,r]	**R88** [γ,θ]	4	0.02	8	0.13	snakebites, malaria, diabetes, wounds, skin diseases, bilharziasis, gastrointestinal diseases	† : NR ‡ : [108] ₸ : [42]
Senna occidentalis (L.) Link. (Fabaceae, KIP000000655)	Lukunda bajani (Luba-kas) [f,q]	R89 [γ,ε]	5	0.02	9	0.15	Diarrhoea, dysentery, constipation, fever, sexually transmitted infections, malaria, diabetes, hepatic diseases	† : [109] ‡ : [32] ₸ : [37]
		**R90** [v, λ]						

Species (Family, HC)	Appellation (Ethnicity)[GT, MT]	Nº Recipe (n=107)[UP,P]	N_C	RCF (n=207)	N_u	UV (n=60)	Non uterotonic indication	Ref.
Sida acuta Burm.f (Malvaceae, KIP000000656)	Tumfumfu (Kikongo) [f,l]	**R91** [γ,ε]	21	0.10	7	0.12	Malaria, ulcers, constipation, diabetes, haemorrhoids, coughs	† : [110] ‡ : [111,112] ¥ : [87]
		**R92** [ν, λ]						
Solanum incanum L (Solanaceae, KIP000000657)	Ndulwe (Bemba) [i,q]	**R93** [γ, δ]	42	0.20	6	0.10	Viral Parasitic diseases, gastro-intestinal diseases, skin diseases, Inflammatory diseases	† : NR ‡ : [113] Ŧ : [85]
Stereospermum kunthianum Cham (Bignoniaceae, KIP000000658)	Kasengele (Lamba) [f,p]	**R94** [γ,δ]	12	0.06	6	0.10	ulcers, skin diseases, Inflammatory diseases, respiratory diseases, sexually transmitted infections	† : NR ‡ : [27] Ŧ : [114]
Strychnos spinosa Lam (Loganiaceae, KIP000111072)	Kisongole (Bemba) [i,p]	**R95** [z,δ]	5	0.02	5	0.08	Malaria, diabetes, snakebites, sexually transmitted infections	† : NR ‡ : [115] Ŧ:[20,21, 37]
Syzygium cordatum Hochst. ex C.Krauss (Myrtaceae, KIP000111074)	Cinyambagany i (Tabwa) [h,r]	**R96** [z,ε]	7	0.03	9	0.15	Malaria, tuberculosis, diabetes, snakebites, sexually transmitted infections, wounds, and respiratory diseases	† : NR ‡ : [40,116] Ŧ : [20]
Tagetes minuta L (Asteraceae, KIP000000659)	Mbaje mbuni (Zela) [d,l]	**R97** [γ, ε]	9	0.04	5	0.08	stomach pain, coughs, gastrointestinal diseases, pain	† : NR ‡ : [57] ¥ : [64]
Thespesia garckeana F.Hoffm. (Malvaceae, KIP000000660)	Makole (Sanga) [f,r]	**R98** [α, δ]	11	0.05	6	0.10	Fever, headache, stomach pain, diabetes, and inflammatory diseases	† : NR ‡ : [19] ¥ : [19]
Trema orientale (L.) Blume (Cannabaceae, KIP000000661)	Nalita (Luba-kat) [i,p]	**R99** [γ,θ]	34	0.16	6	0.10	Asthma, fever, cough, Inflammatory diseases, malaria	† : [117] ‡ : [32] Ŧ : [64]
Uapaca kirkiana Müll. Arg (Phyllanthaceae, KIP000000662)	Masuku (Bemba) [f,p]	**R100** [z,μ]	15	0.07	11	0.18	stomach pain, dysentery, sexually transmitted infections, diabetes, wounds, hyperlipidemia, hypertension, Drepanocytosis, helminth, typhoid fever	† : NR ‡ : [19] Ŧ : [37]
Vitex congolensis var. thomasii (De Wild.) Meerts [syn *Vitex thomasii* De Wild.] (Lamiaceae, KIP000000663)	Mufutu mukwa (Luba-kat) [a,r]	**R101** [β,ε]	36	0.17	5	0.08	Cancer, Inflammatory diseases, diabetes, gastrointestinal diseases,	† : NR ‡ : NR Ŧ : [118]

Species (Family, HC)	Appellation (Ethnicity)[GT, MT]	Nº Recipe (n=107)[UP,P]	N_C	RCF (n=207)	N_u	UV (n=60)	Non uterotonic indication	Ref.
Xylopia aethiopica (Dunal) A.Rich (Annonaceae, KIP000000664)	Njilu luba (Luba-kat) [f,r]	**R102** [β,ε]	7	0.03	11	0.18	rheumatism, diarrhoea, dysentery, cough, asthma, hemorrhoids, malaria, diabetes, wounds, sexually transmitted infections	† : [119] ‡ : [32] ₸ : [37]
Zanthoxylum chalybeum Engl. (Rutaceae, KIP000001016)	Pupwe (Bemba) [f,p]	**R103** [α,μ]	10	0.05	7	0.12	Drepanocytosis, cancer, malaria, diabetes, tuberculosis, gastrointestinal diseases	† : NR ‡ : [25] ₸ : [20,62]
Zanthoxylum zanthoxyloides (Lam.) Zepern. & Timler (Rutaceae, KIP000000665)	Pupwe bimpa (Lamba) [f,r]	**R104** [β,ε]	7	0.03	6	0.10	Drepanocytosis, malaria, fever, tuberculosis, constipation	† : NR ‡ : [32] Ŧ : [120]
Zingiber officinale Roscoe (Zingiberaceae, KIP000111077)	Tangawisi (Swahili) [d,o]	**R105** [α,θ]	9	0.04	10	0.17	Drepanocytosis, cancer, malaria, diabetes, tuberculosis, gastro-intestinal diseases, wounds, Inflammatory diseases, aphrodisiac	† : [121] ‡ : [32,36] ₸ : [20]
Ziziphus abyssinica Hochst. ex A.Rich. (Rhamnaceae, KIP000000666)	Sula (Bemba) [f,r]	**R106** [α,μ]	14	0.07	9	0.15	Drepanocytosis, cancer, diabetes, gastrointestinal diseases, tuberculosis, malaria, wounds, and hypertension	† : NR ‡ : [57] ₸ : [37]
Ziziphus mucronata Willd (Rhamnaceae, KIP000000667)	Kankona (Bemba) [f,r]	**R107** [v,θ]	12	0.06	10	0.17	Drepanocytosis, cancer, diabetes, gastrointestinal diseases, tuberculosis, malaria, wounds, hypertension, stomach pain.	† : NR ‡ : [122] ₸ : [37,62]

Legend :

† (uterotonic Activity)

‡ (uterotonic uses)

₸ (medicinal uses in Lubumbashi)

¥ (medicinal uses in DRC outside Lubumbashi)

Ŧ (medicinal uses outside DRC)

UP: used part, P: Preparation

–***Geographical type*** – CA: a, EA: b; NA-MA: c; NAT: d; NA-TA-SA-MA: e; TA: f; TA-MA: g; TA-SA: h; TA-SA-MA: I; WA: j; WA-SA: k- NA: North Africa; CA: Central Africa; TA: tropical Africa; SA: southern Africa; MA: Madagascar. ***Used part*** : Bulb: u; Flowers: v; Seeds: w; Whole plant: x; Stem bark: y; Fruits: z; Root: α; Root bark: β; Leaves: γ ***Morphological type*** -Annual herb: l; Bulbous geophyte: m; Perennial herb: n; Rhizomatous geophyte: o; Shrub: p; Subshrub: q; Tree: r; Tuberous subshrub: s–- ***Preparation method*** – Cataplasm: δ; Decoction: ε; Infusion: θ; Liniment: λ; Maceration: μ; Ointment: ψ. A *cataplasm* is a medicinal preparation that is applied directly to the skin. The suitability of the plant for this purpose is determined by the condition of the skin. Alternatively, they can be heated in water to soften them, and then gently crushed to extract the active ingredients.

Liniment is a composition of oil (palm or olive) and plant powder in the proportions of 5:2. The ointment is applied to the vaginal muscle. *Ointment* is a preparation obtained from pork fat with a paste of the plant (parts 3:1). According to informants, on average, childbirth occurs within 30 minutes to two hours after the topical application of the herbal preparation.

3.1.1 Diversity of families 'species survey

The 98 plant species identified in this survey are distributed across 98 genera and 43 botanical families. The most represented family is Fabaceae, with a relative citation frequency (RCF) of 0.92, encompassing 16 species. This is followed by Asteraceae (RCF: 0.81, 8 species), and Phyllanthaceae (RCF: 0.54; 8 species (Figure 2).

3.1.2 Variation in geographical, morphological types, and nomenclature.

The plant species documented in our ethnobotanical survey are classified into 11 geographical types. The TA type is the most prevalent, with 49 species, and an RCF of 1.0. They exhibit eight distinct morphological types, with the tree form (38 species; RCF: 1.0) as the most prevalent, followed by annual herbs (21 species; RCF: 1.0) and shrubs (18 species; RCF: 1.0). These species have names in 17 local Congolese languages. The most frequently used language is Bemba (30 species; RCF: 0.99), followed by Luba-Katanga (15 species; RCF: 0.97), and Shi (14 species; RCF: 0.97) (Figure 3).

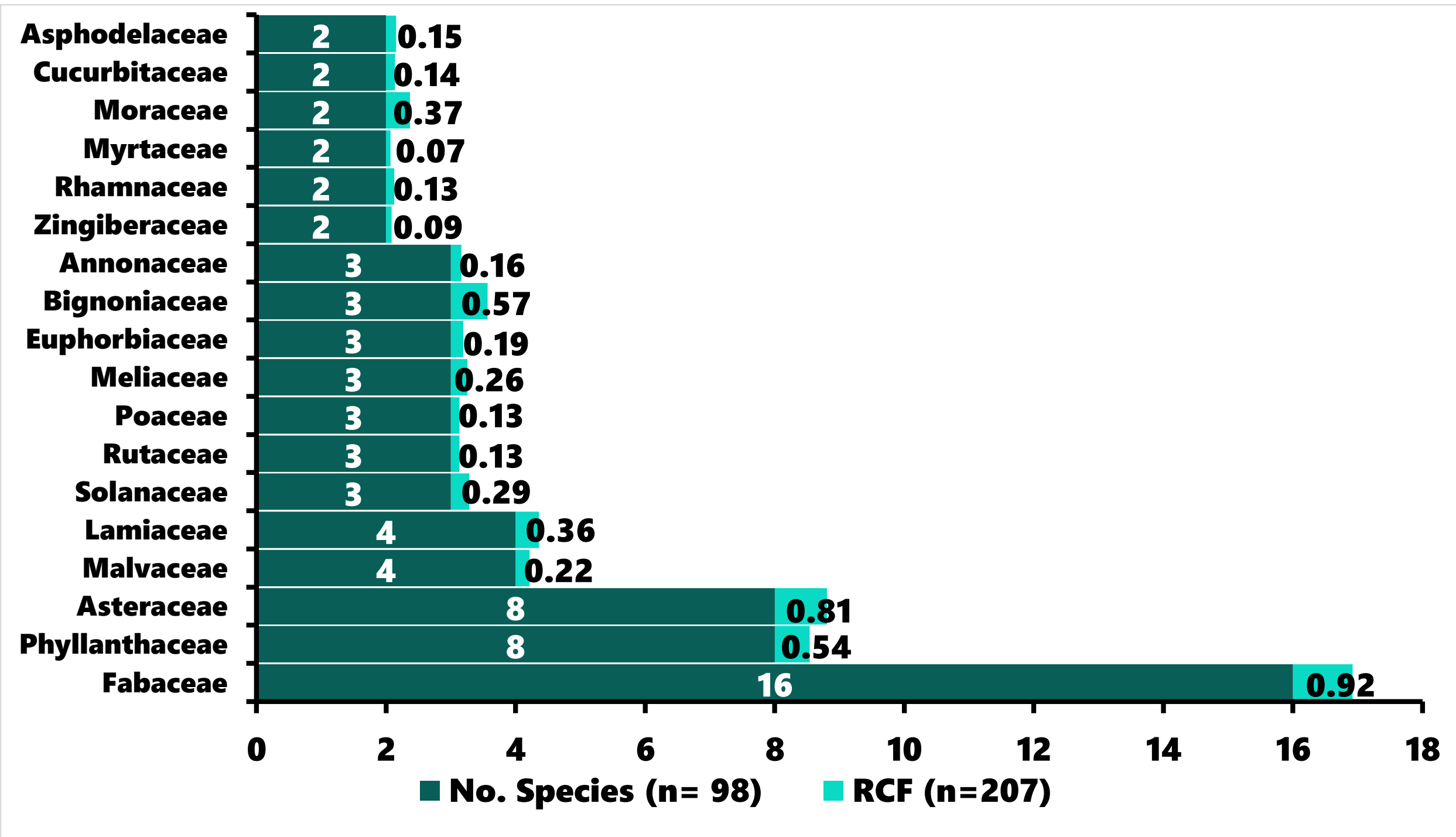

Figure 2: Botanical families with n ≥ 2 plants.

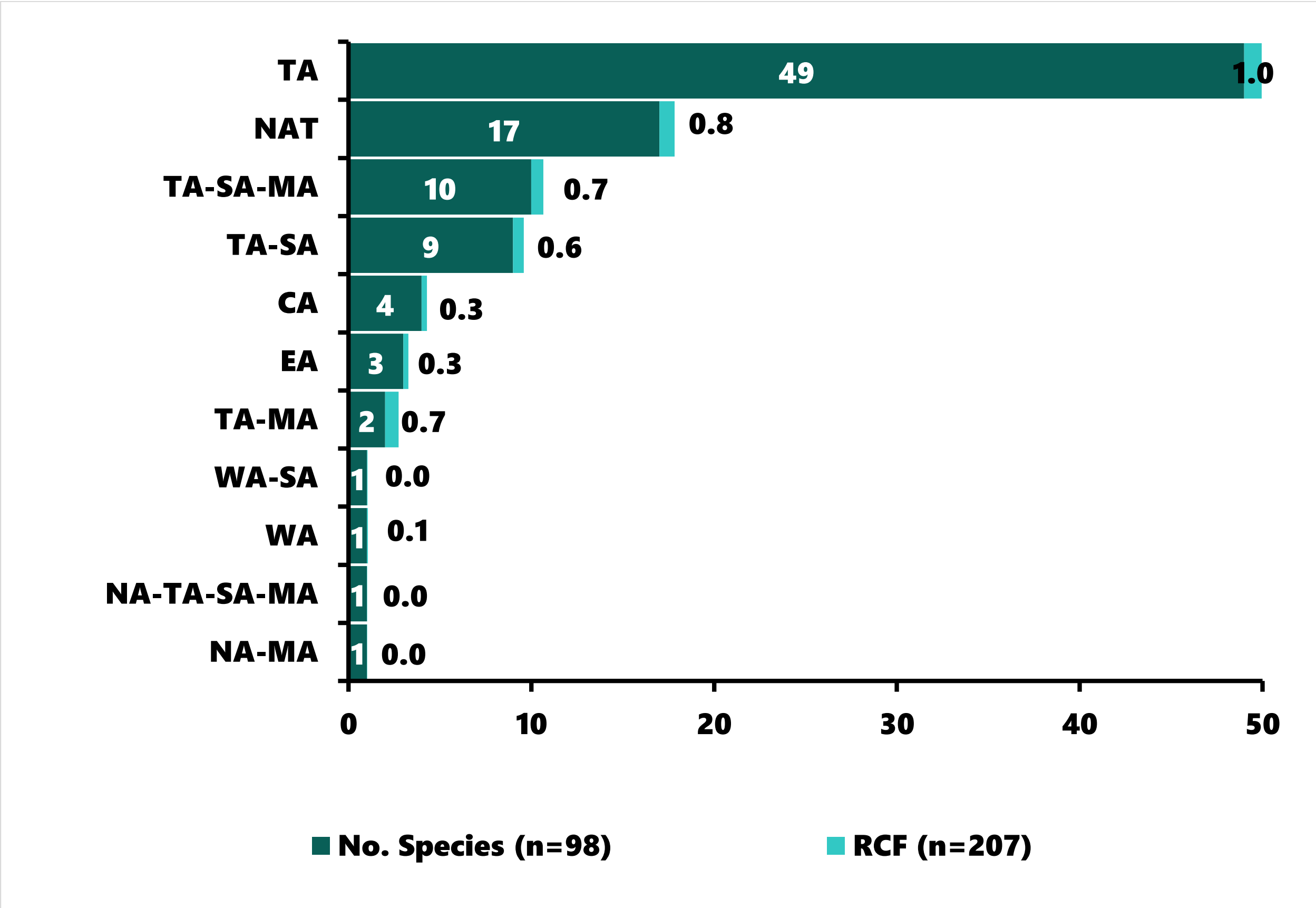

TA
NAT
TA-SA-MA
TA-SA
CA
EA
TA-MA
WA-SA
WA
NA-TA-SA-MA
NA-MA
49
1.0
17
0.8
10
0.7
9
0.6
4
0.3
3
0.3
2
0.7
1
0.0
1
0.1
1
0.0
1
0.0
0
10
20
30
40
50
No. Species (n=98)
RCF (n=207)

A

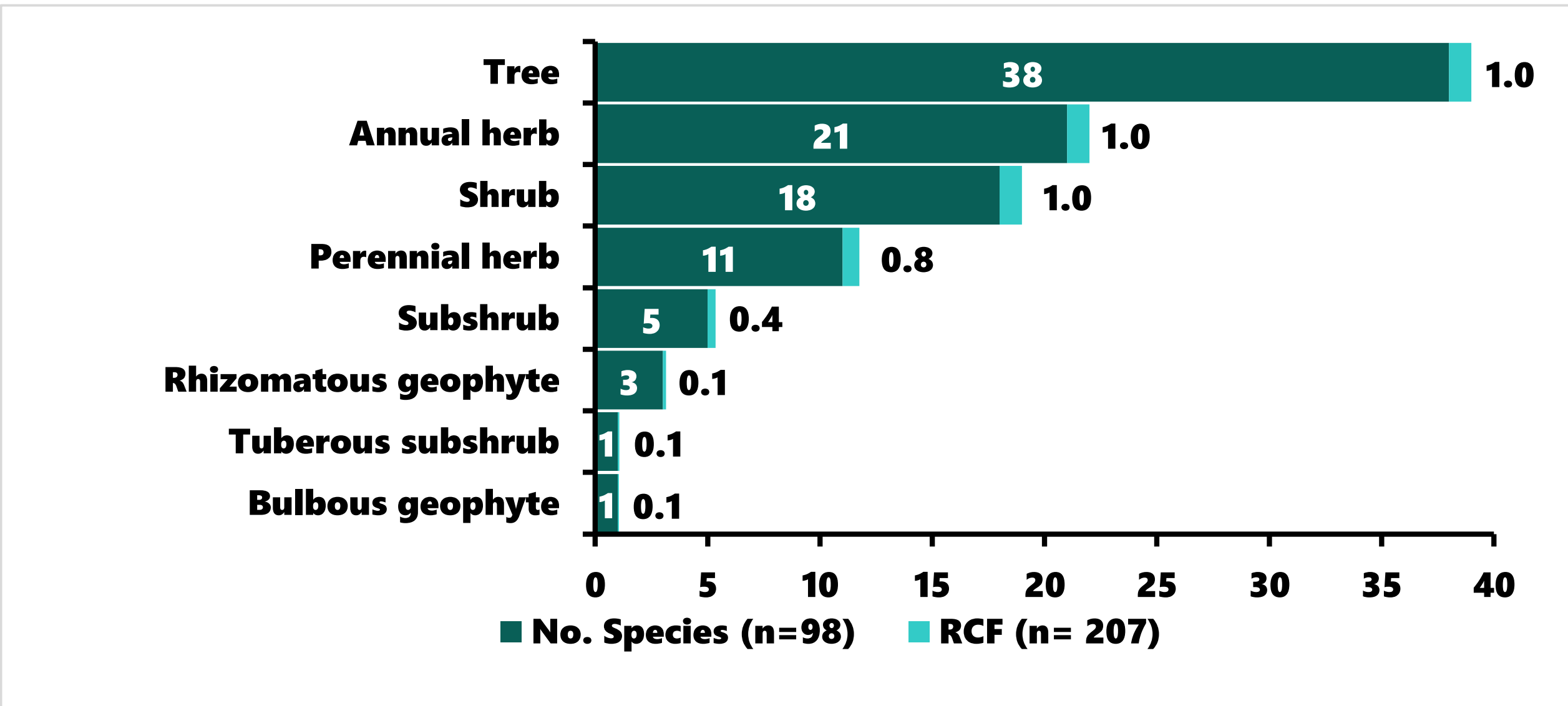

Tree
Annual herb
Shrub
Perennial herb
Subshrub
Rhizomatous geophyte
Tuberous subshrub
Bulbous geophyte
38
1.0
21
1.0
18
1.0
11
0.8
5
0.4
3
0.1
1
0.1
1
0.1
0
5
10
15
20
25
30
35
40
No. Species (n=98)
RCF (n= 207)

B

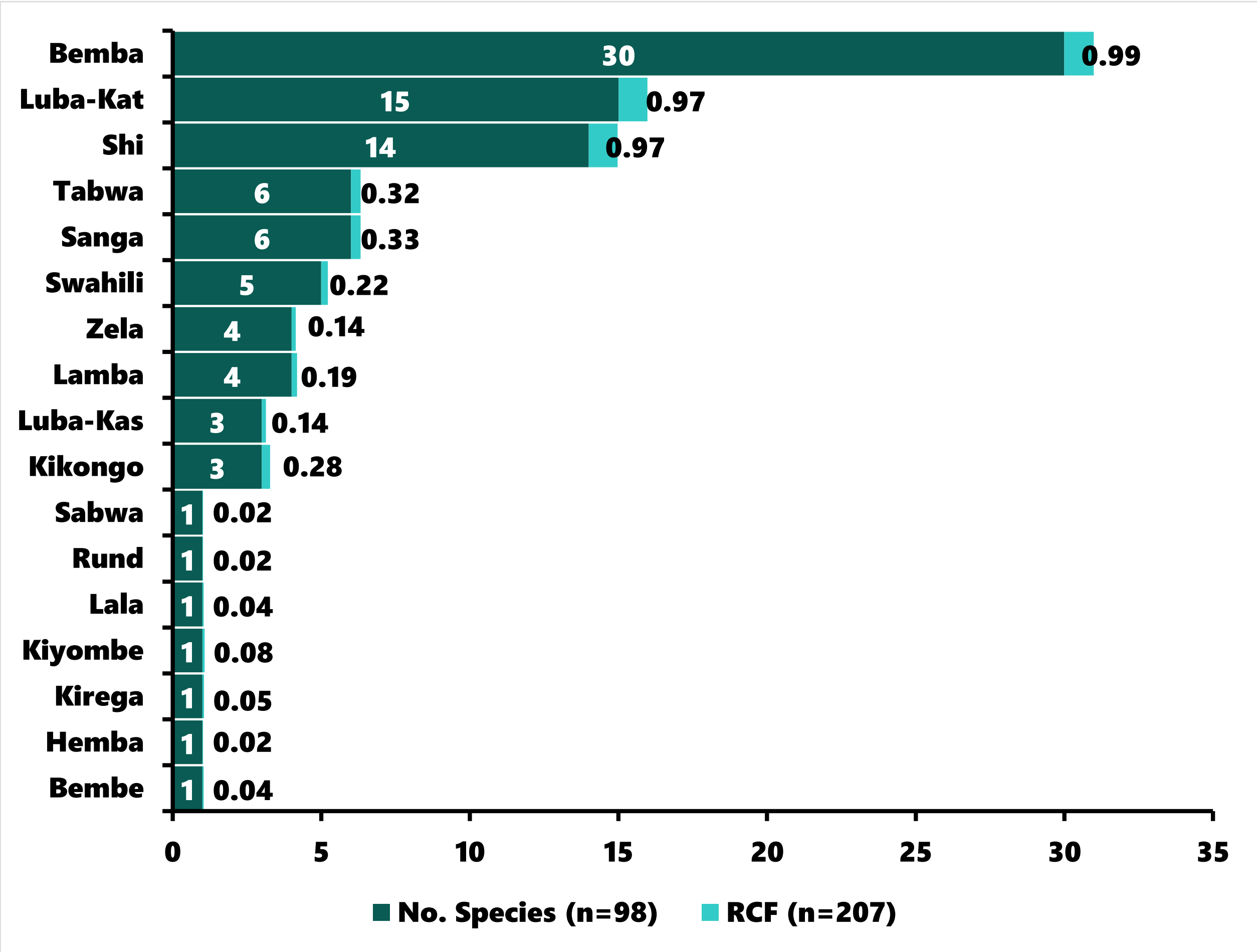

C

Figure 3: Morphological (a) and geographical (b) type of plants, category of informants (c), and situation of the plant with the literature.

Legend: NA: North Africa; CA: Central Africa; TA: tropical Africa; SA: southern Africa; MA: Madagascar.

3.2. Profile of herbal recipes used to facilitate childbirth.

The data collected on 98 plant species during ethnobotanical surveys, documented in this study, that are traditionally used in Lubumbashi to facilitate childbirth led to the identification of 122 oxytocic formulations. Among these, 107 recipes use a single plant (Table 1), whereas 15 formulations incorporate two plant species (Table 2).

These preparations utilize nine different plant organs, with leaves being the most frequently used (33 monoherbal recipes and 10 polyherbal recipes), followed by root bark (18 recipes and 3) (Figure 4).

There are six different preparation methods for plant organs, with decoction (40 recipes using a single plant, and 3 using two plants) and cataplasm (35 and 9 recipes) being the most used methods (Figure 4).

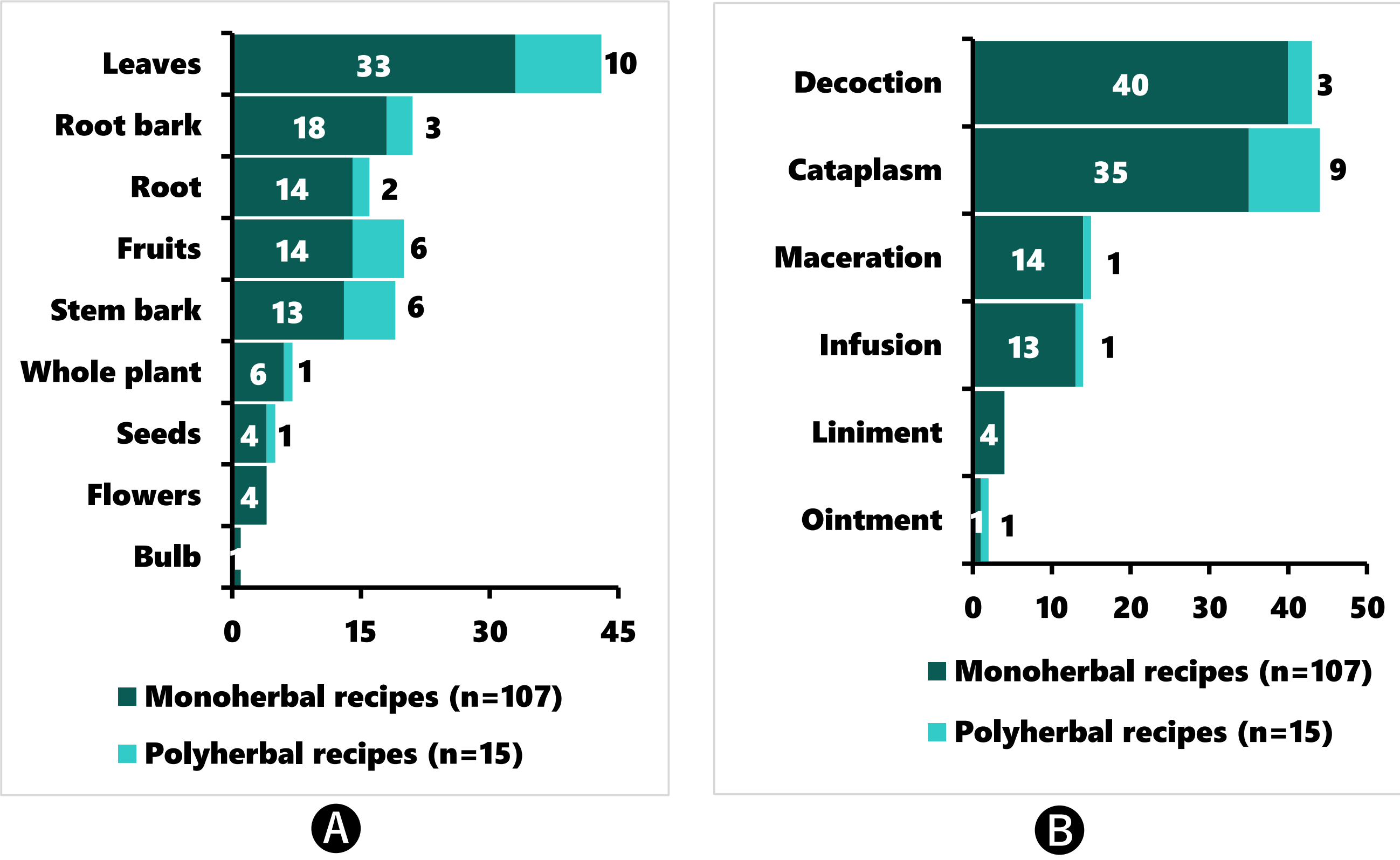

Figure 4: Parts used (A) and mode of preparation of traditional facilitate childbirth formulations (B).

In polyherbal formulations, no plant species out of 30 is recurrent across multiple distinct preparations. The most frequently utilized formulation, designated as R199, consists of a combination of *Hymenocardia acida* root bark, and *Juglans nigra* fruits, exhibiting a relative citation frequency (RCF) of 0.33. This formulation is followed by R116, which is derived from the mixture of *Ekebergia capensis* roots, and *Erythrina abyssinica* stem bark (Table 2).

Table 2 : Traditional polyherbal formulations used to ease childbirth.

N°	Specie 1	Specie 2	N° Recipe	UP (Ratio)	Preparation	Size (RCF) (n=207)
1	*Abrus precatorius*	*Aloe vera*	**R108**	Sd-Lv (2÷1)	Cataplasm	10(0.05)
2	*Annona senegalensis*	*Ageratum conyzoides*	**R109**	Sb-Wp (1÷2)	Cataplasm	12(0.06)
3	*Aframomum alboviolaceum*	*Alchornea floribunda*	**R110**	Fr-Lv (2÷3)	Decoction	15(0.07)
4	*Antidesma venosum*	*Azadirachta indica*	**R111**	Rb-Fr (1÷1)	Ointment	18(0.09)
5	*Boerhavia diffusa*	*Cajanus cajan*	**R112**	Lv- Fr (3÷1)	Cataplasm	22(0.11)
6	*Cissus schmitzii*	*Clausena anisata*	**R113**	R-Lv (2÷1)	Infusion	27(0.13)
7	*Combretum molle*	*Commelina africana*	**R114**	Lv-Sb (2÷1)	Maceration	30(0.15)
8	*Cucurbita pepo*	*Cynodon dactylon*	**R115**	Fr-Wp (2÷1)	Decoction	18(0.09)
9	*Ekebergia capensis*	*Erythrina abyssinica*	**R116**	R-Sb (2÷1)	Decoction	35(0.17)
10	*Ficus capensis*	*Flueggea virosa*	**R117**	Sb-Lv (2÷1)	Cataplasm	31(0.15)
11	*Grona adscendens*	*Asparagus africanus*	**R118**	Lv-Lv (2÷3)	Cataplasm	18(0.09)
12	*Hymenocardia acida*	*Juglans nigra*	**R119**	Rb-Fr(3÷2)	Cataplasm	68(0.33)
13	*Khaya nyasica*	*Kigelia aethiopum*	**R120**	Sb-Sb(3÷2)	Cataplasm	18(0.09)

N°	Specie 1	Specie 2	N° Recipe	UP (Ratio)	Preparation	Size (RCF) (n=207)
14	*Malus domestica*	*Millettia griffoniana*	**R121**	Fr-Rb(3÷2)	Cataplasm	22(0.11)
15	*Ocimum americanum*	*Piliostigma thonningii*	**R122**	Lv-Lv(3÷2)	Cataplasm	27(0.13)

Legend **-** PU-Ratio: Part used and proportion of the mixture; Lv: leaf; R: root; Rb: Root bark; Sb: Stem bark; Fr: fruit; Sd: Seed; N° Recipe. = recipe that makes childbirth easier.

3.3. Other human ailments treated by inventoried species

Beyond their role in facilitating childbirth by producing a uterotonic effect, the 98 documented plant species are also utilized in Lubumbashi folk medicine for the treatment of 60 additional ailments. Among these, gastrointestinal diseases, diabetes, wounds, and malaria represent the three primary indications, both in terms of citation frequency (over 100 citations), and botanical diversity, with each condition involving more than 40 plant species from the recorded cohort (Table 3).

Table 3 : Othe aliments treated by the plants survey

Ailments (n= 61)	Ns	Ailments (n= 61)	Ns	Ailments (n= 61)	Ns
Atheroscleros	1	Dengue fever	3	Dysentery	13
Cataract	1	Constipation	4	Urinary tract infection	14
Cholera	1	Gastritis	4	Hypertension	15
Cold	1	Cardiovascular diseases	5	Drepanocytosis	16
Conjunctivitis	1	Fungal ailments	5	Headache	19
Contraception	1	Intoxications	5	Inflammatory diseases	19
Dental problems	1	Leprosy	5	Hepatic diseases	20
Eye problems	1	Typhoid fever	5	Ulcers	20
HIV/AIDS	1	Hemorrhoids	6	Diarrhea	21
Kidney disorders	1	Parasites diseases	6	Sexually transmitted infections	21
Meningitis	1	sexual dysfunction	7	Coughs	25
Thrombosis	1	Asthma	7	Cancers	27
aphrodisiac	2	Haemorrhoids	7	Respiratory diseases	28
Dysmenorrhea	2	Hyperlipidemia	7	Stomach diseases	28
Epilepsy	2	Pain	7	skin diseases	29
Myoma	2	Rheumatism	8	Fever	37
Neurodegenerative disorders	2	Snake bites	8	gastrointestinal diseases	41
Pruritis	2	Tuberculosis	8	Diabetes	44
bilharziasis	3	Viral diseases	8	Wounds	44
Chickenpox	3	Helminths diseases	9	Malaria	46

Legend : Ns : Numbers of species.

3.4. UV and RCF of plant survey

The relative citation frequency of the plant species documented in this study ranges from 0.01 for *Piliostigma thonningii* to 0.25 for *Kigelia africana*, the species with the highest frequency. This is

followed by *Crassocephalum vitellinum* (RCF: 0.23), *Hylodesmum repandum* (RCF: 0.20), and *Solanum incanum* (RCF: 0.20). The use value of the recorded species varies from 0.05 for *Alchornea floribunda* to 0.32 for *Securidaca longepedunculata*, the species demonstrating the highest utilization. Next are *Asparagus africanus* (UV = 0.27) and *Hibiscus sabdariffa* (UV = 0.25).

3.4. Socio-demographic profile of informants.

The informants surveyed in this study fall into three main categories: housewives (49.8%), herbalists (12.6%) and traditional healers (37.6%). The majority are women (64.7%), with an average age of 41 years, ranging from 17 to 73. They predominantly reside in the Katuba (21.7%) and Annexe (19.8%) municipalities, and most have a secondary education (40.0%). The informants are distributed across six distinct occupational groups, with traditional healers the most numerous (37.5%). Furthermore, a significant proportion (45.4%) have over 11 years' experience applying medicinal plants to facilitate childbirth (Table 3).

Table 3: General characteristics of the informants.

**Class**	**Category**	**Housewives (n=103)**		**Herbalists (n=26)**		**TH (n=78)**		**All Informants (n=207)**
		n_i	**%**	n_i	**%**	n_i	**%**	**%**
Age	15-25	02	**6.3**	00	**00.0**	01	**07.1**	**06.2**
	26-35	14	**32.9**	01	**12.5**	03	**21.4**	**32.1**
	36-45	81	**48.6**	05	**62.5**	02	**14.3**	**47.7**
	46-55	04	**09.5**	01	**12.5**	02	**14.3**	**09.7**
	>55	02	**02.7**	01	**12.5**	06	**42.9**	**04.3**
Experience (Year)	1-5	65	**05.4**	00	**00.0**	01	**07.1**	**05.4**
	6-10	25	**33.7**	01	**12.5**	06	**42.9**	**33.6**
	11-15	05	**46.2**	04	**50.0**	03	**21.4**	**45.4**
	16-20	05	**12.0**	02	**25.0**	02	**14.3**	**12.3**
	21-25	03	**02.7**	01	**12.5**	02	**14.3**	**03.3**
Profession	Trade	20	**19.4**	00	**00.0**	00	**00.0**	**09.7**
	Public sector	21	**20.4**	00	**00.0**	00	**00.0**	**10.1**
	Liberal	59	**57.3**	00	**00.0**	00	**00.0**	**28.5**
	Housework	03	**02.9**	00	**00.0**	00	**00.0**	**01.5**
	TH	00	**00.0**	00	**00.0**	78	**100**	**37.7**
	Herbalist	00	**00.0**	26	**100**	00	**00.0**	**12.5**
Gender	Female	77	**74.8**	16	**61.5**	41	**52.6**	**64.7**
	Male	26	**25.2**	10	**38.5**	37	**47.4**	**35.3**
Municipality of residence	Annexe	23	**22.3**	02	**07.7**	16	**20.5**	**19.8**
	Kamalondo	13	**12.6**	00	**00.0**	02	**02.5**	**07.2**
	Kampemba	15	**14.6**	08	**30.8**	12	**15.4**	**16.9**
	Katuba	12	**11.7**	10	**38.5**	23	**29.5**	**21.7**
	Kenya	16	**15.5**	02	**07.7**	12	**02.5**	**14.5**
	Lubumbashi	18	**17.5**	02	**07.7**	03	**03.8**	**11.2**

Class	Category	Housewives (n=103)		Herbalists (n=26)		TH (n=78)		All Informants (n=207)
	Ruashi	06	**05.8**	02	**07.7**	10	**12.8**	**08.7**
Level of studies	None	24	**23.3**	02	**07.7**	05	**06.4**	**15.0**
	Primary	21	**20.4**	08	**30.8**	23	**29.5**	**25.1**
	Professional	25	**24.3**	03	**11.5**	01	**01.3**	**14.1**
	Secondary	26	**25.2**	10	**38.5**	47	**60.2**	**40.0**
	University	07	**06.8**	03	**11.5**	02	**02.6**	**05.8**

***Legend* -** TH: Traditional healer. Ni: number of Informants. The percentages for each category are shown in bold, as is the overall percentage. Each time, refer to n to determine it.

4. Discussion

4.1. Diversity of medicinal plants used as uterotonic in Lubumbashi.

For millennia, the inhabitants of Lubumbashi have used the flora of the Miombo woodlands to treat various health issues, particularly to facilitate childbirth as uterotonic. In the current investigation, 98 plant varieties were documented according to their application by housewives, herbalists, and traditional healers to support childbirth.

A systematic review of the available literature (Table 1) enabled these taxa to be divided into two main categories, which can be subdivided into five subclasses. The first category comprises 34 species newly documented to have oxytocic uses, while the second category includes 64 species for which oxytocic applications have already been reported in previous studies (Table 1).

The 34 species in the first category were organized into three subclasses:

The first subclasses with nineteen species that are already recognized as medicinal plant in Democratic Republic of Congo, particularly around Lubumbashi, namely: *Aframomum alboviolaceum, Albizia adianthifolia, Antidesma venosum, Bobgunnia madagascariensis, Cissus schmitzii, Combretum molle, Crassocephalum montuosum, Cyanthillium cinereum, Ficus stuhlmannii, Flueggea virosa, Harungana madagascariensis, Hexalobus monopetalus, Hymenocardia acida, Juglans nigra, Khaya nyasica, Kigelia aethiopica, Parinari curatellifolia, Phyllanthus microdendron*, and *Vitex congolensis var. thomasii*;

The second subclass with Eight species documented as medicinal elsewhere in DRC, but not previously reported from Lubumbashi: *Adenia gummifera, Albizia lebbeck, Alchornea floribunda, Baphiopsis parviflora, Boerhavia diffusa, Cassia sieberiana, Hylodesmum repandum*, and *Leonotis nepetifolia*.

The third subclass with seven species whose medicinal use has been established outside the DRC: *Afrothismia mhoroana, Aloe chabaudii, Anchusa ovata, Bombax ceiba, Cochlospermum planchonii, Grona adscendens*, and *Paramollugo nudicaulis*. Together, these 34 taxa are key to understanding the specific role of Lubumbashi's traditional medicine in managing difficult deliveries. Their documentation paves the way for targeted pharmacological research to validate the reported oxytocic effects and enrich the local pharmacopoeia with robust scientific evidence.

Although none of the 34 species catalogued in Lubumbashi have, to date, been explicitly reported or validated for oxytocic activity in the accessible literature, several taxa within the same genera exhibit well-documented uterotonic uses. For example, within the genus Aframomum, *A. melegueta* K.

Schum. (also known as Ataare) is traditionally chewed by the Akoko people of Nigeria to facilitate childbirth [123]. Four species in the genus Albizia have been the subject of ethnobotanical investigations: *Albizia zygia* (DC.) J.F.Macbr : Stem bark is used as a purgative in the Ivory Coast to facilitate childbirth [124]; *Albizia chinensis* Lam.: triterpenoid saponins isolated from the roots significantly modulate uterine contractions [125]; *Albizia ferruginea* (Guill. & Perr.) Benth.: a stem bark decoction is used in Nigeria to aid childbirth [126]; *Albizia gummifera* (J. F. Gmel.) C. A. Meyer: The glycoside 'albitocin', isolated from the bark, increases the frequency and amplitude of spontaneous uterine contractions in various animal models [127]. Additional genera have likewise yielded extracts with demonstrated oxytocic activity: aqueous stem-bark extract of *Adenia globosa* Engl. [128]; methanolic leaf extract of *Alchornea laxiflora* (Benth.) Pax: [72]; aqueous leaf extract of *Cassia fistula* L. [129]; aqueous leaf extract of *Cassia italica* Mill [130]; and aqueous stem bark extract of *Vitex doniana* Sweet [131]. These pharmacological evidence of oxytocic activity in plants of the same genus reported in our current investigations suggest that it is possible to identify plants among those in the inventory that also have proven oxytocic potential. This highlights the importance of conducting targeted pharmacological investigations on Lubumbashi inventory species to confirm and characterize their oxytocic potential.

Within the second category, two subclasses were delineated: The fourth subclass (n = 34) comprises taxa that have traditionally been utilized as medicinal plants, yet for which no pharmacological evidence has been documented thus far. The fifth subclass (n = 30) comprises taxa for which uterotonic activity has been documented in the scientific literature. The confirmed uterotonic effects observed in the fifth subclass serve as empirical validation of the ethnobotanical survey data and strongly suggest that a similar proportion of the unstudied taxa may also possess active uterotonic principles.

The fourth subclass comprises 38 species:*Aerva javanica*, *Ageratum conyzoides*, *Annona senegalensis*, *Asparagus africanus*, *Capsicum annuum*, *Clausena anisata*, *Clematis scabiosifolia*, *Crassocephalum vitellinum*, *Cucurbita pepo*, *Cynodon dactylon*, *Dalbergia boehmii*, *Diplorhynchus condylocarpon*, *Elaeis guineensis*, *Eleusine indica*, *Erythrina abyssinica*, *Euphorbia hirta*, *Ficus capensis*, *Leucas martinicensis*, *Malus domestica*, *Ochna schweinfurthiana*, *Ocimum americanum*, *Phyllanthus amarus*, *Phyllanthus ovalifolius*, *Physalis peruviana*, *Piliostigma thonningii*, *Securidaca longepedunculata*, *Senegalia polyacantha*, *Solanum incanum*, *Stereospermum kunthianum*, *Strychnos spinosa*, *Syzygium cordatum*, *Tagetes minuta*, *Thespesia garckeana*, *Uapaca kirkiana*, *Zanthoxylum chalybeum*, *Zanthoxylum zanthoxyloides*, *Ziziphus abyssinica,* and *Ziziphus mucronata*.

Many of these species are already recorded in diverse African pharmacopoeias for their uterotonic properties, substantiating an ethnomedicinal consensus on their use to facilitate parturition. This interregional convergence implies the conservation of bioactive uterotonic constituents within these species, and justifies further studies, including *ex vivo* assays on isolated uterine muscle, *in vivo* evaluation in gravid rodent models, and the isolation and structural elucidation of the responsible metabolites. Notably, only five of these species (approximately 13 %), those indicated above, have not previously been documented in the traditional lushoise pharmacopoeia. This relatively low proportion highlights the depth and coherence of local botanical knowledge while pinpointing novel candidate species for prioritized pharmacological screening.

4.2. Botanical families, morphology, and endemicity of inventoried plants.

Of the 98 taxa documented in our ethnobotanical surveys, three species predominate (39%), nearly half are endemic to tropical Africa (49%), and 16% belong to the Fabaceae family. The overrepresentation of Fabaceae trees endemic to tropical Africa corroborates earlier ethnobotanical investigations in the katangense region [20,62,85,132–134]. Fabaceae trees exhibit a suite of physiological and ecological traits conferring resilience in hot intertropical environments. Symbiotic associations with rhizobia enable efficient biological nitrogen fixation, permitting establishment on leached, nutrient-poor tropical soils [135]. A deep, laterally branching taproot system enhances water uptake during prolonged dry seasons. Foliar adaptations, compound leaf architecture, and a thick cuticle, minimize transpirational water loss while preserving high photosynthetic capacity under intense insolation [136,137].

Moreover, pronounced phenological plasticity, characterized by leaf abscission in the dry season followed by rapid foliar flush at the onset of rain, synchronizes growth with favorable moisture regimes, and underpins the ubiquity of Fabaceae in intertropical woody communities. Moreover, extant literature validates the existence of a probable correlation between the characteristic phytochemical composition of Fabaceae - notably their richness in triterpenoid, and steroidal saponins, and estrogenic flavonoids (phytoestrogens) - and their documented uterotonic activity. Indeed, preclinical trials have demonstrated that saponin-rich plant fractions induce, in a dose-dependent manner, an increase in the frequency and amplitude of contractions of isolated uterine smooth muscle, thus replicating the traditional use of these plants to facilitate labor [138]. Furthermore, phylogenetic analyses reveal that the Fabaceae lineages most enriched in estrogenic flavonoids, dedicated to fertility and parturition drugs, coincide with the "warm environments"[139]establishing a chemical basis likely to underpin uterotonic efficacy across this family.

Among the documented plant species, spontaneous, uncultivated varieties account for 91 species (92.8%). These wild plants are predominantly used in traditional medicine, offering substantial advantages in terms of accessibility and affordability. This makes them invaluable for healthcare systems with limited resources, such as those of the Democratic Republic of Congo [140,141]. However, utilizing them presents significant challenges. The chemical composition of these plants varies greatly due to environmental influences, which can affect their therapeutic efficacy and safety. Furthermore, the lack of standardization in most cases increases the risk of toxicity and complicates quality assessment. Furthermore, the excessive harvesting of certain species threatens biodiversity, necessitating the implementation of conservation strategies [142,143]. Therefore, although these plants have significant medicinal potential, their integration into healthcare practices necessitates rigorous scientific research and robust regulatory mechanisms to guarantee their safety and efficacy within a structured framework.

4.3. Uterotonic recipes of medicinal plant survey

In the 122 uterotonic recipes inventoried during our surveys, most of which used a single plant, the leaf (35%) was the most used organ, and decoction (35%) the most prevalent method of preparation in mono-herbal formulation. However, among all the recipes, poultices are the most common method, which is justified by the fact that most recipes are for local application. While decoction is the most

common method of preparation in several ethnobotanical studies carried out in the region [62,132,150,133,134,144–149]Various organs seem to be the most popular according to the studies. Both the leaves [20,62,85,132,144,151,152], sometimes roots [85,118,145,146,149] or stem bark [134,153,154].

The use of leaves in traditional medicine offers several significant advantages over other medicinal plant organs, both pharmacologically and ecologically. From a therapeutic point of view, leaves are often rich in the synthesis of most bioactive metabolites [42,155,156]. In addition, their anatomical structure facilitates the extraction of active ingredients, improving the efficacy of traditional preparations [42]. Ecologically, leaf harvesting is generally less destructive than root or stem bark harvesting, contributing to the preservation of plant species and the sustainability of medicinal practices [157]. Moreover, leaves are often available in greater quantities and over a longer period of the year, making them more accessible to local communities. However, their active ingredient content can vary according to season and environmental conditions, requiring rigorous standardization to guarantee their therapeutic efficacy.

During this study, fifteen polyherbal formulations (12 %) each combine two plant species, an approach with ambivalent outcomes. On one hand, phytotherapy exploits the synergy of bioactive constituents and mutual masking of adverse effects to achieve a broader therapeutic spectrum with reduced toxicity. On the other hand, the complexity of these mixtures can prove counterproductive: unforeseen interactions, excessive dilution of key active compounds, and botanical variability related to geographic origin or harvest season all impede the standardization and reproducibility of treatments [20,154]. Added to these challenges are the risks of cross-allergies among multiple species and the uncertain safety of compositions that lack robust clinical validation, which may reveal toxic or mutagenic properties. Nevertheless, the enduring use of most of these formulations for over twenty-five years attests to their acceptable safety profile and adequate efficacy: without a favorable benefit–risk balance, they would have been discontinued long ago.

4.4. Plants highlighted by this survey

In this study, we consider that the plants highlighted are notably the two most frequently cited plants: *Kigelia africana* (RCF: 0.25) & *Crassocephalum vitellinum* (RCF: 0.23); the two most widely used plants: *Securidaca longepedunculata* (UV: 0.27) & *Hibiscus sabdariffa* (UV = 0.25) as well as the two plants most frequently cited among those reported for the first time as oxytocic plants thanks to our present study: *Hylodesmum repandum* (RCF: 0.20) & *Crassocephalum montuosum* (RCF: 0.19) (Figure 5).

Indeed, a high degree of citation convergence around a species reflects a high Informant Consensus Index. This homogeneity of knowledge suggests empirical pharmacological validation: the more a plant is cited for its oxytocic properties, the more likely it is to contain bioactive compounds that could account for these properties. Furthermore, the most frequently used plants define the actual Use Value (UV) within the community. In contexts where access to modern healthcare is limited, these species are the pillars of health in this environment. Studying them is a public health emergency, both to validate their safety and to prevent the risks of toxicity associated with non-standardized dosages. However, it should be noted that poorly documented plants are not insignificant; they reflect the

endemic nature of knowledge and the bio-cultural specificity of the environment, which can very easily lead to original research that may result in new knowledge.

If a case had to be made for a single plant per category, such as most cited, most used or new discovery, then it would be the bark of *Kigelia africana*, the leaves of *Securidaca longepedunculata* and the leaves of *Hylodesmum repandum*, which are the plants that stand out most in this study (Figure 5).

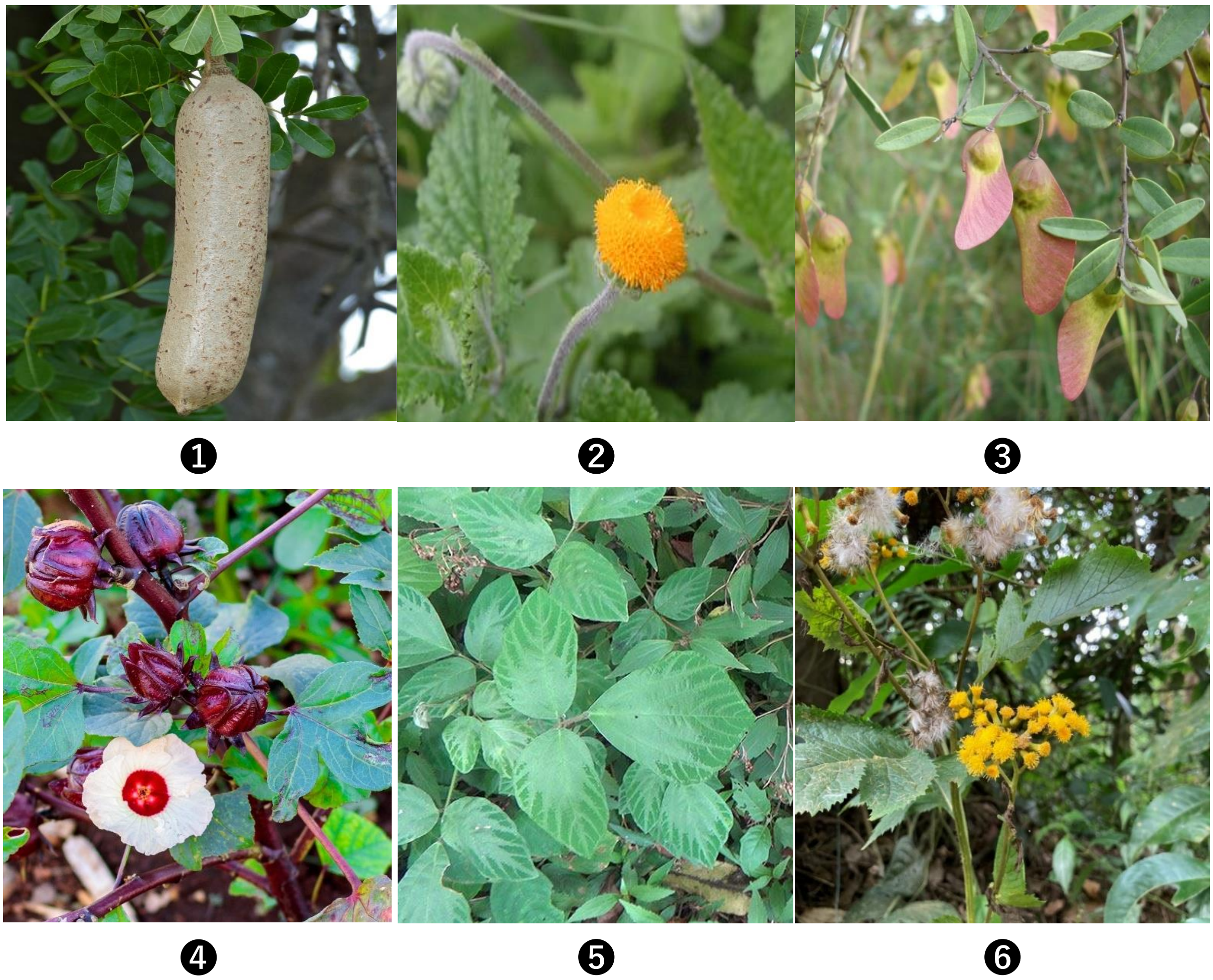

Figure 5 : Plants highlighted in this study in order of appearance: *Kigelia africana*, *Crassocephalum vitellinum*, *Securidaca longepedunculata*, *Hibiscus sabdariffa*, *Hylodesmum repandum*, *Crassocephalum montuosum*. (Source: Kipopo INERA station).

The stem bark of *Kigelia africana* (Lam.) Benth. is a key part of African traditional medicine, where it has been used to treat a wide range of illnesses for a long time. As we all know, traditional applications cover a range of dermatological disorders, like eczema and wound healing, as well as gastrointestinal disturbances, including dysentery [30,154]. They also cover critical gynaecological indications, like labour induction and control of postpartum haemorrhage [158,159]. Recent drug studies have shown that these traditional uses are legit and have reported strong antimicrobial, anti-inflammatory, and antioxidant effects, as well as the ability to kill melanoma and renal carcinoma cell lines [160,161]. These bioactivities arise from a range of phytochemicals, such as naphthoquinones (e.g., lapachol and kigelinone), iridoid glycosides (e.g., verminoside and specioside), and polyphenolic compounds (e.g., gallotannins and flavonoids) [162,163]. All this evidence shows that *K. africana* stem bark is important in terms of pharmacology, and that it should be further explored as a source of therapeutic agents.

Although the roots of *Securidaca longepedunculata* are most frequently employed in traditional medicine, the leaves are also used for a wide range of ethnomedical indications, including epilepsy, headache, abdominal pain, infertility, snakebite, toothache, cancer, dermal infections, dislocated jaw, contraceptive purposes, and placental expulsion [154,164]. Pharmacological investigations have provided evidence supporting several bioactivities of leaf extracts, notably antimicrobial activity [165,166], immunomodulatory and anti-inflammatory effects [167], antiplasmodial activity[168], antioxidant properties [169], and anticonvulsant activity [170]. These activities have been correlated with the presence of diverse phytochemical classes, principally xanthones, saponins, and flavonoids [165,171], some of which have been reported to possess oxytocic potential [172–176].

The leaves of *Hylodesmum repandum* (syn. *Desmodium repandum*) are a cornerstone of traditional primary healthcare, widely utilized as hemostatic poultices for treating fresh wounds, ulcers, and skin infections, and in decoctions to manage fever, dysentery, and pain. These traditional applications are supported by ethnopharmacological studies demonstrating significant antimicrobial, antioxidant, and anti-inflammatory activities, particularly against pathogens such as Staphylococcus aureus, as well as antimalarial activity. This biological efficacy is attributed to a distinct phytochemical profile rich in flavonoids (specifically isoflavonoids), tannins, and alkaloids, groups also reported among those with uterotonic potential [173–175].

5. Limits of the study

The findings of this study should be interpreted in light of several limitations: (*i*) the absence of quantitative data on harvesting pressure and its potential impact on the population dynamics of highly sought-after taxa; (*ii*) the lack of clinical validation for interview-derived claims concerning therapeutic indications and mechanisms of action; (*iii*) the study's emphasis on ethnomedicinal knowledge related to facilitation of childbirth combined with a limited geographic scope, which may have resulted in incomplete documentation of other local uses of the recorded taxa; (*iv*) the omission during field surveys of critical usage details, such as administration conditions, contraindications, dosage regimens, and adverse effects, underscoring the need for targeted pharmacological, toxicological, and dosage-finding studies for each reported taxon; and (*v*) insufficient documentation of herbal formulations and ancillary traditional applications beyond obstetric use, which warrants systematic ethnobotanical, phytochemical, and pharmacological follow-up investigations.

6. Conclusion

The findings of this study support that in Lubumbashi, both traditional healers and women community leaders routinely employ medicinal plants as uterotonic agents in the management of childbirth complications. The taxa identified for uterotonic use frequently possess additional ethnomedicinal applications, and several of the uses recorded locally correspond with reports from other African pharmacopoeias, indicating regional convergence in traditional obstetric practice.

These ethnobotanical findings provide a rationale for prioritized pharmacological and clinical investigation. Systematic phytochemical profiling, bioassay-guided fractionation, and *in vitro* and *in vivo* evaluation of uterotonic activity are required to validate efficacy and to identify the bioactive constituents responsible for the observed effects. Concurrent toxicological and finding studies, together with documentation of traditional formulations and administration regimens, are essential to assess safety and translational potential.

Finally, given the dual importance of these species for maternal health and local livelihoods, future research should integrate conservation assessments and sustainable use strategies alongside biomedical validation to ensure that any development of plant-derived uterotonics is both scientifically robust and culturally and ecologically responsible.

7. Data available statement

The data used in this study are provided and included within the article.

8. Conflicts of interest

The authors declare that there is no conflict of interest concerning the data presented in this study.

9. Funding statement

No funding exists

10. Acknowledgments

The authors would like to thank the Kipopo Herbarium for identifying plant species. They also thank traditional healers, herbalists, and Lubumbashi households for acquiring ethnobotanical data.

11. Authors' contribution

Design, formatting, and analysis of data: BCV; development of methods, and drafting of the first manuscript: BCV; study checks: ONP, CSM, MMF, JCN, MMP, SNT, BBM, SKG, MKG, NIE,MM,BAS,MMH; manuscript corrections: BCV, PNP and LSJB.

12. References

[1] Onambele L, Guillen-aguinaga S, Guillen-aguinaga L, Ortega-leon W, Montejo R, Alas-brun R, et al. Trends , Projections , and Regional Disparities of Maternal Mortality in Africa (1990 – 2030): An ARIMA Forecasting Approach. *Epidemiologia* 2023;**4**:322–351.

[2] Onambele L, Ortega-leon W, Guillen-aguinaga S, Jo M, Yoseph A, Guillen-aguinaga L, et al. Maternal Mortality in Africa : Regional Trends (2000 – 2017). *Int J Environ Res Public Heal* 2022;**19**:13146.

[3] WHO, UNICEF, UNFPA, WBG, UNDESA. *Trends in maternal mortality estimates 2000 to 2023*. World Health Organization: Geneva, Switzerland, 2025.

[4] Osadolor OO, Osadolor AJ, Osadolor OO, Enabulele E, Ezi A. Access to health services and health inequalities in remote and rural areas. *Janaki Med Coll J Med Sci* 2022;**10**:70–74.

[5] Esther K O, Kojo E, Mensah G. Maternal Mortality and Legal Accountability : Examining Government Responsibility for Preventable Deaths. *J Law Crim Justice* 2025;**13**:49–73.

[6] Aydar M Z, Ilnara I B, Viktor A M. Uterotonic drugs in obstetric bleeding prevention and

treatment. *J Obstet women's Dis* 2021;**70**(1):77–88.

[7] Felis S, Quaroni S, De Simone A. Uterotonic Agents. *Am J Med Clin Res Rev* 2023;**2**(12):1–14.

[8] Hertha Kasiku H, Larai A-A, Aboua YG. Scope of Medicinal Plants for Uterotonic, Tocolytic, and Wellness Effects in Pregnant Women: A Cultural Perspective. In: Megh R. G, Junaid Ahmad M, Ademola Olabode A, editors. *Curative and Preventive Properties of Medicinal Plants*. Apple Academic Press: New York, USA, 2023: 410.

[9] Sumankuuro J, Baatiema L, Crockett J, Young J. Women ' s use of non - conventional herbal uterotonic in pregnancy and labour : evidence from birth attendants. *BMC Pregnancy Childbirth* 2022;**22**:600.

[10] Medjedovic E, Stanojevic M, Jonuzovic-Prosic S, Ribic E, Begic Z, Cerovac A, et al. Artificial intelligence as a new answer to old challenges in maternal-fetal medicine and obstetrics. *Technol Heal Care* 2024;**32**(3):1273–1287.

[11] Id G, Yunas I, Aj D, Podesek M, Tobias A, Mj P, et al. Uterotonic agents for preventing postpartum haemorrhage: a network meta-analysis (Review). *Cochrane Database Syst Rev* 2025;**4**:CD011689.

[12] Sichalwe MM, Nangi NW, Daniel L, Egbobe JO, Tavengana G. Prevalence and determinants of medicinal plants utilization during labour among women of reproductive age in Butiama , Tanzania : A community-based cross-sectional study. *PLoS One* 2025;**233**:1–16.

[13] Tripathi V, Stanton C, Anderson FWJ. Traditional preparations used as uterotonics in Sub-Saharan Africa and their pharmacologic effects. *Int J Gynecol Obstet* 2013;**120**:16–22.

[14] Roqaiya M, Begum W, Majeedi SF, Saiyed A. A Review on Herbs with Uterotonic Property. *J Phytopharm* 2015;**4**(3):190–196.

[15] Cobos DS, Luisa M, López N, Alejandrina E, Hernández G, Cruz BV. Uterotonic activity of Heimia salicifolia leaves. *J Med Plants Stud* 2019;**7**(41653):98–102.

[16] Chanda A, Simwinga F, Kaonga P, Gono-bwalya A, Prashar L. The Uterotonic Screening of the Root Extract of Azanza garckeana (Malvaceae) on Isolated Wistar Rat Uterine Smooth Muscles. *Evidence-Based Complement Altern Med* 2020;**20**:ID 8873180.

[17] Neuenschwander C, Wu K, Degolier T, Neuenschwander C. An Investigation into the Use of an Herbal Labor Induction Tincture Containing Black Cohosh , Cramp Bark , Partridgeberry , and Motherwort on Contractile Responses Produced from Isolated Strips of Mouse Uterine Tissues. *Pharmacogn J* 2021;**13**(6):1478–1487.

[18] Nama MC, Lunkuntu KH, Malonga KF, Kahumba BJ, Kakoma S. Contribution de la médecine traditionnelle à base des plantes utérotoniques à la santé de la reproduction en RDC. *Int J Soc Sci Sci Stud* 2021;**1**(4):1–18.

[19] Esther KI, Kleph KZ, Mariette KK, Sylvie YN, Andre WOT, Niclette L, et al. Plants Used by Pregnant Women at Kipushi City in Democratic Republic of Congo: Prevalence and Indications. *Open Access Libr J* 2017;**04**(02):1–8.

[20] Valentin BC, Philippe ON, Melman M, Henry MM, Salvius BA, Baptiste LSJ. Ethnomedical knowledge of plants used in alternative medicine to treat hemorrhoidal diseases in Lubumbashi, Haut-Katanga province, Southern Democratic Republic of Congo. *BMC Complement Med Ther* 2024;**24**(1):365.

[21] Bashige C V, Pierre KI, Manya MH, Kasali mushagalusa F, Okusa NP, Lumbu SJ. Ethnobotanical study of plants used by traditional healers in Lubumbashi (Democratic Republic

of Congo) in the management of typhoid fever. *GSC Biol Pharm Sci* 2022;**21**(01):265–286.

[22] Leonti M. The relevance of quantitative ethnobotanical indices for ethnopharmacology and ethnobotany. *J Ethnopharmacol* 2022;**288**:1–19.

[23] Nwodo O, Botting J. Uterotonic activity of extracts of the seeds of Abrus precatorius. *Planta Med* 1983;**47**(4):230–3.

[24] Oladimeji AV, Valan MF. The Potential Therapeutic Advantage of Abrus precatorius Linn. an Alternative to Glycyrrhiza glabra: A Review. *J Pharm Res Int* 2021;**32**(40):79–94.

[25] Adia MM, Savina A, Jane N, Joel M, Godwin A, Esezah KK, et al. Medicinal plant species used for contraception and reproductive health care in rural Uganda. *Heliyon* 2025;**11**(1):e41518.

[26] Kasali FM, Kadima JN, Peter EL, Mtewa AG, Ajayi CO, Tusiimire J, et al. Antidiabetic Medicinal Plants Used in Democratic Republic of Congo: A Critical Review of Ethnopharmacology and Bioactivity Data. *Front Pharmacol* 2021;**12**:1–40.

[27] Maman Noura O, Ilagouma AT, Compaore S, Mahamane Idi Issa A, Bogninou GSR, Ouédraogo R, et al. Ethnopharmacological investigation of plants used for the management of childbirth complications in Niger. *Sci African* 2024;**24**:e02216.

[28] Abate L, Tadesse MG, Bachheti A, Bachheti RK. Traditional and Phytochemical Bases of Herbs, Shrubs, Climbers, and Trees from Ethiopia for Their Anticancer Response. *Biomed Res Int* 2022;**22**:1589877.

[29] Tafese Awulachew M. Hand Book of Common Ethiopian Traditional Medicinal Plants: Their Parts and Uses for Human and Animal Treatments. *J Dis Med Plants* 2021;**7**(3):48.

[30] Valentin BC, Philippe ON, Henry MM, Salvius BA, Suzanne MK, Kasali M. Ethnomedical Knowledge of Plants Used in Nonconventional Medicine for Wound Healing in Lubumbashi , Haut-Katanga Province , DR Congo. *Sci World J* 2024;**2024**:1–33.

[31] Tlhapi DB, Ramaite IDI, Anokwuru CP, Ree T Van, Hoppe HC. In Vitro Studies on Antioxidant and Anti-Parasitic Activities of Compounds Isolated from Rauvolfia caffra Sond. *Molecules* 2020;**25**:3781.

[32] Koman SR, Kpan WB, Yao K, Ouattara D. Medicinal uses of plants by traditional birth attendants to facilitate childbirth among djimini women in Dabakala (Center-north of Côte d'Ivoire). *Ethnobot Res Appl* 2021;**21**:1–12.

[33] Mbuta KKU, Mwima K, Kavuna M, Kalambayi M, Kazembe K, Booto K, et al. *Plantes médicinales de traditions Province de l'Equateur – R.D. Congo*. Institut de Recherche en Sciences de la Santé: Kinshasa, RDC, 2012.

[34] Agbo MO, Okoye FBC, Ebi GC, Osadebe PO. Alchornea floribunda (Müll. Arg.) - A review of its phytochemistry and biological activities. *Trop J Pharm Res* 2020;**19**(5):1113–1120.

[35] Gutiérrez AL, Barrantes JB, Navarro MP, Monge MCO, Vargas MJC, Redondo GLM. General aspects about Allium sativum – a review. *Ars Pharm* 2021;**62**(4):471–481.

[36] Kamel N, Boullani R El. Use of Medicinal Plants during Pregnancy , Childbirth and Postpartum in Southern Morocco. *Healthcare* 2022;**10**:2327.

[37] Bashige VC, Philippe ON, Henry MM, Salvius BA, Suzanne MK, Kasali M. Ethnomedical Knowledge of Plants Used in Nonconventional Medicine for Wound Healing in Lubumbashi , Haut-Katanga Province , DR Congo. *Sci World J* 2024;**2024**:4049263.

[38] Anywar G, Tugume P, Kakudidi EK. A review of Aloe species used in traditional medicine in

East Africa. *South African J Bot* 2022;**147**:1027–1041.

[39] Gobvu V, Poshiwaa X, Benhura MA. An ethnoveterinary survey of medicinal plants used to treat poultry diseases in drylands of Zimbabwe. *Ethnobot Res Appl* 2024;**29**(31):1–20.

[40] Nikolajsen T, Nielsen F, Rasch V, Sørensen PH, Ismail F, Kristiansen U, et al. Uterine contraction induced by Tanzanian plants used to induce abortion. *J Ethnopharmacol* 2011;**137**:921–925.

[41] Hajj M El, Holst L. Herbal Medicine Use During Pregnancy : A Review of the Literature With a Special Focus on Sub-Saharan Africa. *Front Pharmacol* 2020;**11**:866.

[42] Valentin BC, Burck KM, Philippe ON, Simbi L. Ethnomedicinal Knowledge of plants used in Alternative Medicine to treat Acnes in Lubumbashi (Democratic Republic of Congo). *GSC Biol Pharm Sci* 2025;**31**(2):137–160.

[43] Jaradat N, Qadi M, Abualhasan MN, Al-lahham S, Al-rimawi F, Hattab S, et al. European Journal of Integrative Medicine Carbohydrates and lipids metabolic enzymes inhibitory , antioxidant , antimicrobial and cytotoxic potentials of Anchusa ovata Lehm . from Palestine. *Eur J Integr Med* 2020;**34**:101066.

[44] El-Ishaq A, Alshawsh MA, Chik Z Bin. Evaluating the oestrogenic activities of aqueous root extract of Asparagus africanus Lam in female Sprague-Dawley rats and its phytochemical screening using Gas Chromatography-Mass Spectrometry (GC/MS). *PeerJ* 2019;**26**(7):e7254.

[45] Mfengwana P-M-AH, Mashele SS. Medicinal Properties of Selected Asparagus Species : A Review. In: *Phytochemicals in Human Health*. Great Portland Street, London, 2019: 1–23.

[46] Bussmann RW, Paniagua-zambrana NY. Ethnobotany in the Andes and the Amazon in a world of Nagoya Protocol and post SARS-CoV-2 pandemic. *Botany* 2022;**108**(11):97–108.

[47] Evans Ngwenah F, Mahamat O, Christopher T. Hypoglycaemic, anti-hyperlipidemic, and anti-inflammatory activities of Asparagus africanus (Asparaceae) extract on high-fat diet/streptozotocin-induced diabetes in Wistar rats. *Asian Pac J Trop Biomed* 2024;**14**(12):532–539.

[48] Latham P, Mbuta AK ku. Plantes utiles du Bas-Congo,République Démocratique du Congo (2e Ed). *Inst Rech en Sci la Santé* 2014;:409.

[49] Konda KM, Bavukinana N LP, Mbembe B, Konda KR, Dumu L, Mafuta M, Kelela B, Itufa Y KK. Plantes médicinales de traditions de la RD. Congo - données préliminaires. *Inst Rech en Sci la Santé* 2015;**1**:1–438.

[50] Adeniyi IA, Olufunke O, Usman IM, Owu DU. Unraveling the reproductive potential of selected flavonoids in Biden pilosa: A comprehensive review. *Phytomedicine Plus* 2025;**5**(2):100784.

[51] Jan M, Khare RK, Mir TA. Medicinal plants used during pregnancy and childbirth in baramulla district of jammu and kashmir, india. *Ethnobot Res Appl* 2021;**22**:70–80.

[52] Chiribagula Valentin B, Ndjolo Philippe O, Mboni Henry M, Mushagalusa Kasali F. Ethnomedicinal Knowledge of Plants Used in Nonconventional Medicine in the Management of Diabetes Mellitus in Kinshasa (Democratic Republic of the Congo). *Evidence-Based Complement Altern Med* 2023;**2023**:4621883.

[53] Das G, Shin HS, Ningthoujam SS, Talukdar A Das, Upadhyaya H, Tundis R, et al. Systematics, phytochemistry, biological activities and health promoting effects of the plants from the subfamily bombacoideae (Family malvaceae). *Plants* 2021;**10**:651.

[54] Katuura E, Kalabika E, Lubega A. Uterotonic Potential of Selected Plants Used by Ugandan Local Communities in the Treatment of Malaria. *European J Med Plants* 2018;**24**(2):1–12.

[55] Konda K, Bavukinina N, Latham P, Mbembe B, Konda K, Dumu L, et al. Plantes médicinales de la RD. Congo- données préliminaires. *Inst Rech en Sci La Santé* 2015;**1**:438.

[56] Olatunji-Bello I, Obijeih T, Mojiminiyi F. Tocolytic effect of Cajanus cajan (in-vitro studies using the rat uterus). *Nig Q J Hosp Med* 2000;**10**(4):279–81.

[57] Kamatenesi-Mugisha M, Oryem-Origa H. Medicinal plants used to induce labour during childbirth in western Uganda. *J Ethnopharmacol* 2007;**109**(1):1–9.

[58] Ngalamulume HB, Divengi J-PN, Mbuyi PL, Assumani IZ, Mukeba FB, Mutwale PK, et al. Ethnobotanical, Phytochemical and Pharmacological Studies of Capsicum annuum L. (Solanaceae) in the Healing of Traumatic Wounds (Case of Eye Wounds): A Mini Review. *Asian Plant Res J* 2022;**10**(1):46–56.

[59] Lampiao F, Maliwichi-Nyirenda C, Mponda J, Tembo L, Clements C. A preliminary investigation of the effects of labour inducing plant, cissampelos mucronata, on the outcomesof pregnancy using rat models. *Malawi Med J* 2018;**30**(3):159–161.

[60] Tshibangu JN, Chifundera K, Kaminsky R, Wright AD, König GM. Screening of African medicinal plants for antimicrobial and enzyme inhibitory activity. *J Ethnopharmacol* 2002;**80**(1):25–35.

[61] Maroyi A. A Synthesis and Review of Medicinal Uses, Phytochemistry and Pharmacological Properties of Cissampelos mucronata A. Rich. (Menispermaceae). *J Pharm Nutr Sci* 2020;**10**(5):213–222.

[62] Numbi Wa Ilunga E, Mbayo Kitambala M, Muya K, Lachenaud O, Mukekwa Maloba J, Lumbu Simbi JB, et al. Ethnobotanical survey and antimycobacterial activities of plants used against tuberculosis in Lubumbashi, DR Congo. *Trop Med Health* 2025;**53**(1):1–13.

[63] Omara T, Kiprop AK, Kosgei VJ, Kagoya S. Clausena anisata (Willd.) Hook.f. ex Benth. (Rutaceae): ethnomedicinal uses, phytochemistry, pharmacological activities, toxicity, and clinical application. *Tradit Med Res* 2022;**7**(6):51.

[64] Bashige C V, Bakari AS, Ndjolo PO, Kahumba BJ, Duez P, Lumbu SJ. Ethnobotanical study of plants used as antimalarial in traditional medicine in Bagira in Eastern RD Congo. *J Pharmacogn Phytochem* 2020;**9**(4):1–14.

[65] Dall'Acqua S, Kumar G, Sinan KI, Sut S, Ferrarese I, Mahomoodally MF, et al. An insight into Cochlospermum planchonii extracts obtained by traditional and green extraction methods: Relation between chemical compositions and biological properties by multivariate analysis. *Ind Crops Prod* 2020;**147**(January):112226.

[66] Kabre Z, Yerbanga RS, Meda RN, Haro A, Fofana A, Kam SE, et al. Traditional Uses of Seven Medicinal Plants for Malaria Treatment in Bobo-Dioulasso , Burkina Faso. *Adv Infect Dis* 2025;**15**(1):79–95.

[67] Attah AF, O'Brien M, Koehbach J, Sonibare MA, Moody JO, Smith TJ, et al. Uterine contractility of plants used to facilitate childbirth in Nigerian ethnomedicine. *J Ethnopharmacol* 2012;**143**(1):377–382.

[68] Kembelo P, Tuenter E, Vanhove W, Katula H, Van Damme P, Pieters L. Phytochemical profiling by UPLC-ESI-QTOF-MS of Commelina africana, widely used in traditional medicine in DR Congo. *South African J Bot* 2023;**157**:325–34.

[69] Valentin BC, Salvius BA, Simbi LJ-B. Antibacterial and Antioxidant Activities, Toxicity, and Physicochemical Properties of Crassocephalum montuosum (S Moore) Milne-Redh and Crassocephalum picridifolium (DC) S Moore. *Adv Pharmacol Pharm Sci* 2024;**24**:9954073.

[70] Olarewaju OO, Fajinmi OO, Arthur GD, Coopoosamy RM, Naidoo KK. Food and medicinal relevance of Cucurbitaceae species in Eastern and Southern Africa. *Bull Natl Res Cent* 2021;**45**(1):208.

[71] Ndayazi D, Kasali FM, Runigi B, Vianney J, Pierre YY, Kabangu T. Medicinal Plants Used in Complementary Medicinal for the Treatment of Burns in Rutshuru Territory (North-Kivu , Democratic Republic of Congo). *J Pharm* 2018;**8**(4):72–81.

[72] Bafor EE, Omogiade GU. Ex-Vivo Uterine Activity of Theobroma Cacao (Malvaceae) Aqueous Seed Extract and *Cymbopogon Citratus* (Poaceae) Aqueous Leaf Extract - A Preliminary Investigation. *Niger J Basic Appl Sci* 2020;**27**(2):108–118.

[73] Mercy GT, Amon A, Patrick EO, Casim UT, Clement OA, Esther K. Medicinal plants used in gynecological procedures in Uganda. *J Med Plants Res* 2020;**14**(4):185–194.

[74] Vwakyanakazi M, Petit P. *Bunganga ya Mici : Guérisseurs et plantes médicinales à Lubumbashi*. Observatoire du Changement Urbain (OCU) – Coopération universitaire au développement (CUD): Lubumbashi, 2004.

[75] Maroyi A. The importance of ecological, biological and medicinal properties of Diplorhynchus condylocarpon (Müll. Arg.) Pichon (Apocynaceae) in Tropical Africa. *Ecol Environ Conserv* 2021;**27**:27–35.

[76] Sewram V, Raynor MW, Mulholland DA, Raidoo DM. The uterotonic activity of compounds isolated from the supercritical fluid extract of Ekebergia capensis. *J Pharm Biomed Anal* 2000;**24**(1):133–145.

[77] Sadgrove NJ, Mitaine-Offer AC, Khumalo G, Van Wyk BE. An overview of the phytochemistry of medicinal bark (trunk, stem or root) from the most popular southern African species. *Phytochem Rev* 2025;**5**:1–21.

[78] Soha SAS, Dougnon TJ, Ohouko FHO, Kpodekon MTT, Youssao AKI. Activités biologiques et utilisations de Elaeis guineensis (Jacq) et de Khaya senegalensis (Desr) en médecine traditionnelle humaine et vétérinaire. *Int J Biol Chem Sci* 2019;**13**(1):525–542.

[79] Rastogi S, Pandey MM, Rawat AKS. An ethnomedicinal, phytochemical and pharmacological profile of Desmodium gangeticum (L.) DC. and Desmodium adscendens (Sw.) DC. *J Ethnopharmacol* 2011;**136**(2):283–296.

[80] Murumba DB, Jeruto P, Nyarang N. Ethnobotanical survey of the cultural uses , threats and conservation status of flora in Kiptogot sub-location , Endebess subcounty , western Kenya. *J Med Plants Stud* 2025;**13**(1):122–131.

[81] Kalu IK, Irene II, others. Action of contractile fraction of Vernonia amygdalina. Del ethanolic extract on hormonal profile of Estrogen and Progesterone in female albino Wistar rats. *Glob Res J Sci Nat* 2015;**1**(2):1–5.

[82] Ogundeko Timothy Olugbenga, Osagie Ize Anuoluwapo, Bitrus James, Okoye Nkiruka Philomena, Ogbole Emmanuel Anebi, Edugbe Adikpe Emmanuel, et al. The uterotonic screening of calyx extracts of Hibiscus sabdariffa on estrogenized isolated Uterus of Wister strain albino rats. *Int J Front Biol Pharm Res* 2022;**3**(1):037–045.

[83] Cedrick Mutombo Shakalenga, Henry Manya Mboni, Salvator Nsenga Nkulu, Gaël Nzuzi

Mavungu, Cynthia Kibwe Mwenya, James Maloba Mwinensenge, et al. Mineral elements analysis and total flavonoids content in the fresh leaves from two varieties of Hibiscus sabdariffa L. consumed as vegetable in Lubumbashi (DR Congo). *World J Adv Res Rev* 2021;**9**(1):147–155.

[84] Manya Mboni H, Faes M, Fraselle S, Compaoré M, Salvius BA, Joseph KB, et al. Evaluating phytochemical constituents and in-vitro antiplasmodial and antioxidant activities of Fadogiella stigmatoloba, Hygrophylla auriculata, Hylodesmum repandum, and Porphyrostemma chevalieri extracts. *Heliyon* 2023;**9**(9):e20103.

[85] François DO, Eddy NK, James MM, Marsi MK, Justin KK, Lambert MM, et al. Ethnobotanical Studies of Reputed Aphrodisiac Plants Used in Traditional Medicine in Haut-Katanga in DR of Congo. *Int J Innov Sci Res Technol* 2024;**9**(5):3317–3334.

[86] Bashige CV, Bakari AS, Martin BB, Lumbu SJB. Ethnomedical knowledge of plants used in traditional medicine in Mampa village , Haut-Katanga province , Democratic Republic of Congo. *Res Sq* 2024;:1–50.

[87] Pathy Kibungu K. *Ethnobotanical study and characterization of medicinal plants used by populations of Kisantu and Mbanza-Ngungu territories, Kongo-Central Province (DR Congo).* 2024.

[88] Bashige C, Bakari A, Okusa N, Kahumba B, Duez P, Lumbu S. Ethnobotanical study of plants used as antimalarial in traditional medicine in Bagira in Eastern RD Congo. *J Pharmacogn Phytochem* 2020;**9**(4):1–14.

[89] Kamatenesi-Mugisha M, Makawiti DW, Oryem-Origa H, Olwa-Odyek, Nganga J. The oxytocic properties of Luffa cylindrica (L.) M. Roem. and Bidens pilosa L., traditionally used medicinal plants from western Uganda. *Afr J Ecol* 2007;**45**(SUPPL. 3):88–93.

[90] Wanda GJMK, Starcke S, Zierau O, Njamen D, Richter T, Vollmer G. Estrogenic activity of griffonianone C, an isoflavone from the root bark of Millettia griffoniana: Regulation of the expression of estrogen responsive genes in uterus and liver of ovariectomized rats. *Planta Med* 2007;**73**(6):512–518.

[91] Banzouzi J, Prost A, Rajemiarimirato M, Ongoka P. Traditional uses of the African Millettia species (Fabaceae). *Int J Bot* 2008;**4**(4):406–420.

[92] Hoosen F, Derek L, Tagatsing N, Maurice F, Emmanuel Y. The anti - cancer potential of active compounds extracted from Millettia griffoniana on pancreatic and colorectal cancer cells. *Adv Tradit Med* 2024;**24**(3):789–800.

[93] Yusuf A, Abdullahi M, AA M. Ethnobotany, Phytochemistry and Pharmacology of Ochna Schweinfurthiana: a Review. *Univers J Pharm Res* 2020;**4**(6):75–79.

[94] Bashige C V, Bakari AS, Okusa NP, Longanga OA, Duez P, Lumbu SJ-B. Ochna schweinfurthiana F. Hoffm (Ochnaceae). *Phytothérapie* 2024;**22**:57–61.

[95] Thulin A, Cjbg G, Thulin M, Bidault E, Boullet V, Heidari N, et al. Phylogeny and systematics of Paramollugo Phylogeny and systematics of Paramollugo (Molluginaceae). *Candollea* 25AD;**79**(2):179–228.

[96] Okunrobo L, Nwagwuogbe S, Bafor E. Phytochemical Evaluation and In Vitro Inhibitory Effect of the Methanol Extract and Partitioned Chloroform Fraction of the Stem Bark of Pentaclethra Macrophylla Benth (Fabaceae) on Non-Pregnant Rat Uterus. *West African J Pharm* 2012;**23**(1):19–26.

[97] Ajala TO, Abraham A, Keck CM, Odeku OA, Elufioye TO, Olopade JO. Shea butter (Vitellaria paradoxa) and Pentaclethra macrophylla oil as lipids in the formulation of Nanostructured lipid carriers. *Sci African* 2021;**13**:e00965.

[98] Kipute DD, Katayi AL, Luambua NK. Quantitative Ethnobotany of Multiple- - Use Species and Management of the Yangambi Biosphere Reserve in the Democratic Republic of the Congo. *Ecol Evol* 2025;**15**:e71110.

[99] Mbayo M, Kalonda E, Muya R, Tshisand P, Kanangila A, Bakari S, et al. Test d ' activité antimitotique et étude chimique préliminaire de quelques Euphorbiaceae du Katanga méridional (RDC). *J Adv Bot Zool* 2016;**2012**(2):1–13.

[100] Doughari JH, Sunday D. Antibacterial Activity of Phyllanthus muellerianus. *Pharm Biol* 2008;**0209**:400–405.

[101] Tshibangu DST, Kavugho FS, Kabengele CN, Masunda AT, Bongo GN, Kasiama GN, et al. Phytochemical study and evaluation of the antidiabetic and antihyperglycemic activities of the fruit extracts of Physalis peruviana L. (Solanaceae). *Phytomedicine Plus* 2025;**5**(1):4–10.

[102] Sheeja E, Joshi SB, Jain DC. Antifertility activity of stems of plumbago rosea in female albino rats. *Pharm Biol* 2008;**46**(12):920–927.

[103] Chhodavadiya N, Vyas K, Patani P. A Review on Plumbago Zeylanica Linn. : As an Antifrtility Agent. *J Cardiovasc Dis Res* 2023;**14**(12):2023.

[104] Mboni HM, Keymeulen F, Ngezahayo J, Amuri SB, Mutombo EK, Byanga JK, et al. Antimalarial herbal remedies of Bukavu and Uvira areas in DR Congo: An ethnobotanical survey. *J Ethnopharmacol* 2020;**249**(1):112422.

[105] State E, Ekiti A, State E. Uterotonic effect of methanol extract and fractions of Psidium guajava l. (myrtaceae) stem bark on female white albino rats. *J Pharm Sci* 2023;**2**(1):36–44.

[106] Kaluwa Kaingu C, Oduma JA, Kanui T. Preliminary investigation of contractile activity of Ricinus communis and Euclea divinorum extracts on isolated rabbit uterine strips. *J Ethnopharmacol* 2012;**142**(2):496–502.

[107] Moroole MA, Materechera SA, Mbeng WO, Aremu AO. Medicinal plants used for contraception in South Africa: A review. *J Ethnopharmacol* 2019;**235**:19–27.

[108] Abba U, Kankara S, Nasir M, Lawal U. Ethnobotanical survey and pharmocognistic features of Senegalia polyacantha (Willd.) Seigler & Ebinger. *Niger J Pharm Sci* 2023;**23**(2):115–27.

[109] Govindaraj Y, Melanaphuru V, Gupta S, Agrahari V, Nema RK. Antifertility Activity of the Ethanolic Extract of Cassia occidentalis, Derris brevipes Variety Brevipes and Justicia simplex. *World J Chem* 2009;**4**(2):118–122.

[110] eze obioma, nwodo okwesili, ogugua victor, joshua parker. Uterine Contractile Effect of Ethanol Extracts of Sida acuta Burm F. Leaves. *J Investig Biochem* 2016;**5**(2):37.

[111] Ngolsou F, Obono P, Ndongo MN, Minkande JZ. Analyse Phytochimique des Extraits Aqueux de Sida Acuta et Triumfetta Cordifolia , Deux Plantes Utilisées pour Faciliter l ' Accouchement en Médecine Traditionnelle au Cameroun. *Heal Sci Dis* 2022;**23**(2S):14–18.

[112] Poli S, Gbekley E, Alognon A, Montant M, Hoinsou Y, Gbati L, et al. Monographie d'une plante médicinale : Sida acuta Burm. *J la Rech Sci l'Université Lomé* 2023;**25**(3):9–21.

[113] Paniagua-Zambrana N, Bussmann R, Kikvidze Z. Solanum dulcamara L. Solanum lycopersicum L. Solanum melongena L. Solanum nigrum L. Solanum tuberosum L. Solanaceae.

In: Bussmann R, Elachouri M, Kikvidze Z, editors. *Ethnobotany of the Mountain Regions of Eastern Europe: Carpathians*. Springer Cham: Cham, Switzerland, 2024: 31–50.

[114] Ior LD, Pekuk BN, Otimenyin SO. Behavioural and anticonvulsant properties of methanol extract of the stem bark of Stereospermum kunthianum Cham in mice. *J Pharm Bioresour* 2021;**18**(1):47–52.

[115] Thothela SNS, Kola E, Dalu MTB, Ndhlovu PT. Indigenous Knowledge and Utilisation of Strychnos spinosa Lam. in Sub-Saharan Africa: A Systematic Review of Its Medicinal, Nutritional, and Cultural Significance. *Diversity* 2025;**17**(4):228.

[116] Maroyi A. Syzygium cordatum hochst. Ex krauss: An overview of its ethnobotany, phytochemistry and pharmacological properties. *Molecules* 2018;**23**(1084):1–18.

[117] Gbolade AA, Adedokun OA, Ibrahim TL. Assessment of contractile effect of Trema orientale (cannabaceae) root bark extracted with methanol on uterus of non-pregnant rat. *Sci World J* 2024;**19**(4):955–958.

[118] Mavungu GN, Mutombo CS, Numbi DM, Nsenga SN, Muyumba WN, Pongombo CS, et al. Smallholders' knowledge about healing goat gastrointestinal parasite infections with wild plants in southern DR Congo. *Front Pharmacol* 2023;**14**:1124267.

[119] Nwafor AV, Ohanme EO, Ofonakara U, Eze CN, Okoroafor FC, Iwe CB, et al. Oxytocic and Abortifacient Activities of Xylopia Aethiopica Ethanol Fruit Extract on Female Wistar Rats. *Int J Hum Heal Sci* 2024;**08**(03):268–274.

[120] Innocent UO, Joseph CN, Emmanuel CA, Chibuike CU. zanthoxylum species: A comprehensive review of traditional uses, phytochemistry, pharmacological and nutraceutical applications. *Molecules* 2021;**26**(13):2–38.

[121] Ghayur MN, Gilani AH. Inhibitory activity of ginger rhizome on airway and uterine smooth muscle preparations. *Eur Food Res Technol* 2007;**224**(4):477–481.

[122] Adamolekun MM, Akpor OA, Olorunfemi O, Akpor OB. Traditional medicine use during pregnancy and labor in African context: A scoping review. *J Integr Nurs* 2023;**5**(1):66–72.

[123] Oluwajuyigbe B, Ige O. Ethnobotanical plants used for fertility control and delivery enhancement among Akoko and Ose aborigines in Ondo state, Nigeria. *Ife J Sci* 2025;**27**(1):129–145.

[124] Konan EK, Tie MS. Plantes utilisées dans le traitement des troubles gynéco-obstétriques par les peuples Abbey et Krobou d ' Agboville côte d ' Ivoire. *Phytothéra* 2009;**09**:262–274.

[125] Lipton BYA. An active glycoside from albizia species and its action on isolated uterus and ileum. *J Pharm Pharmacol* 1963;**15**(1):816–824.

[126] Antimicrobial and uterine smooth muscle activities of Albizia ferruginea extracts. *Boletín Latinoam y del Caribe Plantas Med y Aromáticas* 2006;**5**(2):31–35.

[127] He Y, Wang Q, Ye Y, Liu Z, Sun H. The ethnopharmacology , phytochemistry , pharmacology and toxicology of genus Albizia : A review. *J Ethnopharmacol* 2020;**257**:112677.

[128] Sinei K, Mwangi J. An in vitro Study on the Oxytocic Action of Adenia Globosa Engl. *East Cent African J Pharm Sci* 2015;**18**:96–99.

[129] Yadav R, Jabalpur M, Jain GC. Effect of aqueous extract of seeds of Cassia fistula on the uterine biochemical milieu of female albino rats. *Pharmacologyonline* 2017;**1**:859–867.

[130] No A, Bharathi V, Raja M, Students UG. Review Article on Senna italica Mill. *Int J Pharm Sci*

Rev Res 2018;**52**(08):44–46.

[131] Ladeji O, Udoh FV, Okoye ZSC. Activity of Aqueous Extract of the Bark of Vitex doniana on Uterine Muscle Response to Drugs. *Phytother Res* 2005;**806**:804–806.

[132] Kalonji M, Muya RK, Mutombo EK, Cimanga BCC, Numbi EWI, Kahumba JB. Aperçu ethnobotanique de plantes réputées antipaludéennes utilisées dans la ville de Lubumbashi et ses environs , dans le Haut-Katanga en RD Congo. *Ethnopharmacologia* 2019;**61**(6):75–84.

[133] Bashige C V, Alombong AG, Kamwimba MA, Bakari AS, Okusa NP. Ethnobotanical study of medicinal plants used in the treatment of sexual dysfunctions in traditional medicine in Kampemba-Lubumbashi, DR Congo. *World J Adv Res Rev* 2020;**7**(3):016–032.

[134] Valentin BC, Pierre KI, Henry MM, Mushagalusa F, Philippe ON, Simbi L, et al. Ethnobotanical study of plants used by traditional healers in Lubumbashi (Democratic Republic of Congo) in the management of typhoid fever. *GSC Biol Pharm Sci* 2022;**22**(1):164–284.

[135] Sprent JI, Parsons R. Nitrogen fixation in legume and non-legume trees. *F Crop Res* 2000;**65**:183–196.

[136] Leal A, Fidelis A, de Araujo M, Cozin B, Martins A. Belowground systems in tropical savanna: Fabaceae morphoanatomical traits and their relation to fire. *Plant Biol* 2024;**26**(7):1118–30.

[137] Siddiqui S. Global patterns and drivers of species and genera richness of Fabaceae. *Front Plant Sci* 2025;**16**:1581814.

[138] Fromm O, Degolier T. The contractile capabilities of various herbal constituents on uterine smooth muscle and their shared constituent presence involved with anti- inflammatory / antioxidant mechanisms. *J Pharmacogn Phytochem* 2021;**10**(4):28–37.

[139] Thaweepanyaporn K, Thompson JB, Vasudevan N, Hawkins JA. Phylogeny, ethnomedicinal use and the distribution of phytoestrogens in the Fabaceae. *Front Pharmacol* 2025;**16**:1583365.

[140] Alqethami A. Ethnobotanical Assessment of the Diversity of Wild Edible Plants and Potential Contribution to Enhance Sustainable Food Security in Makkah , the Kingdom of Saudi Arabia. *Diversity* 2025;**17**:785.

[141] Mokgehle SN, Du Plooy CP, Araya HT, Amoo SO, Mokgehle SN, Pofu KM, et al. Medicinal plant cultivation for sustainable use and commercialisation of high-value crops. *S Afr J Sci* 2022;**118**(7):1–7.

[142] Kumari R, Deepali, Bhatnagar S. Biodiversity Loss: Threats and Conservation Strategies. *Int J Pharm Sci Rev Res* 2021;**68**(37):242–254.

[143] Mykhailenko O, Jalil B, Mcgaw LJ, Echeverría J, Takubessi M, Heinrich M. Climate change and the sustainable use of medicinal plants : a call for “ new ” research strategies. *Front Pharmacol* 2025;**15**:1496792.

[144] Kalonda E, Mbayo M, Muhume S, Kasereka M, Mulamba J, Manya H, et al. Ethnopharmacological survey of plants used against malaria in Lubumbashi city (D.R. Congo). *J Adv Bot Zool* 2014;**1**(2):1–8.

[145] Muya K, Tshoto K, Cioci CC, Aseho MM, Kalonji M, Byanga K, et al. Survol ethnobotanique de quelques plantes utilisées contre la schistosomiase urogénitale à Lubumbashi et environs. *Phyothérapie* 2014. doi:10.1007/s10298-014-0877-z.

[146] Mbayo KM, Kalonda ME, Tshisand TP, Kisimba KE, Mulamba M, Richard MK, et al. Contribution to ethnobotanical knowledge of some Euphorbiaceae used in traditional medicine

in Lubumbashi and its surroundings (DRC). *J Adv Bot Zool* 2016;**4**(2):1–16.

[147] Bashige C V, Bakari, Amuri S, Mbuyi KS, Kahumba BJ, Duez P, Lumbu SJ. Étude ethnobotanique, phytochimique et évaluation de l'activité antiplasmodiale de 13 plantes réputées antipaludéennes dans la commune du Kenya (Lubumbashi, RDC). *Phytothérapie* 2020;**18**(2):71–80.

[148] Bashige-Chiribagula V, Manya-Mboni H, Ntabaza-Ndage V, Numbi Ilunga E, Bakari-Amuri S, Kalonda Mutombo E, et al. Étude ethnobotanique, biologique et chimique de plantes réputées anticariogènes à Lubumbashi – RD Congo. *Phytotherapie* 2017;**15**(1):2–9.

[149] Amuri B, Maseho M, Lumbu S, Pierre D, Byanga K. Ethnobotanical survey of herbs used in the management of diabetes mellitus in Southern Katanga Area/DR Congo. *Pan African Med Journal* 2018;**30**(2018). doi:10.11604/pamj.2018.30.218.11718.

[150] Maloba MJ, Muyumba NW, Kasanya KJ, Mutombo SC, Mbayo KM, Ngoy KE, et al. Ethnobotanical survey and preliminary chemical screening of 14 plants reputed antigastritis in Lubumbashi and surroundings (DR Congo). *Int Eur Ext Enablement Sci Eng Manag* 2020;**8**(12):62–69.

[151] Okombe V, Simbi JL, Stévigny C, Vandenput S, Pongombo C, Duez P. Traditional plant-based remedies to control gastrointestinal disorders in livestock in the regions of Kamina and Kaniama (Katanga province , Democratic Republic of Congo). *J Ethnopharmacol* 2014;**153**(3):686–693.

[152] Bashige-Chiribagula V, Bakari-Amuri S, Mbuyi-Kalonji S, Kahumba-Byanga J, Duez P, Lumbu-Simbi JB. Étude ethnobotanique, phytochimique et évaluation de l'activité antiplasmodiale de 13 plantes réputées antipaludéennes dans la commune du Kenya (Lubumbashi, RDC). *Phytothérapie* 2017;(September). doi:10.1007/s10298-017-1152-x.

[153] Bashige C V, Manya MH, Ntabaza N V, Numbi IE, Bakari AS, Kalonda ME, et al. Étude ethnobotanique, biologique et chimique de plantes réputées anticariogènes à Lubumbashi – RD Congo. *Phytothérapie* 2017;**15**(1):2–9.

[154] Valentin BC, Martin BB, Salvius BA, Baptiste LSJ. Ethnomedical Knowledge of Plants Used in Traditional Medicine in Mampa Village, Haut-Katanga Province, Democratic Republic of the Congo. *Sci World J* 2025;**2025**(1):2635735.

[155] Twaij BM, Hasan N. Bioactive Secondary Metabolites from Plant Sources : Types , Synthesis , and Their Therapeutic Uses. *Int J Plant Biol* 2022;**13**:4–14.

[156] June M-, Dar RA, Shahnawaz M, Ahanger MA, Majid I. Exploring the Diverse Bioactive Compounds from Medicinal Plants : A Review. *J Phytopharm* 2023;**12**(3):189–195.

[157] Devi K, Chaudhary V, Kalia S, Mishra R. Ustainable harvesting of non timber forest produce (NTFPS). An operational manual. *Indian Counc For Res Educ* 2020;**1**:1–91.

[158] Nabatanzi A, Nkadimeng SM, Lall N, Kabasa JD, Mcgaw LJ. Ethnobotany, Phytochemistry and Pharmacological Activity of Kigelia africana (Lam.) Benth. (Bignoniaceae). *Plants* 2020;**9**:753.

[159] Dossou-yovo HO, Vodouh FG, Kindomihou V. Investigating the Use Profile of Kigelia africana (Lam .) Benth . through Market Survey in Benin. *Conservation* 2022;**2**:275–285.

[160] Mihaylova R, Bebrivenski N, Zheleva-dimitrova D, Simeonova R. Integrative Evaluation of Kigelia africana Fruit Extract : Broad-Spectrum Anticancer Activity , Synergism with Cisplatin and Mechanistic Insights in Colorectal Carcinoma. *Molecules* 2026;**31**:107.

[161] Maiyo ZC, Njeru SN, Toroitich FJ, Indieka SA, Obonyo MA. Ethnobotanical study of medicinal plants used by the people of Mosop , Nandi County in Kenya. *Front Ph* 2024;**14**:1328903.

[162] Murthy SSN, Jainab SIB, Bhuvaneswari G, Subhashini S, Radhakrishnan S, Saravanan S. Phytochemical screening , antioxidant activity and spectral characterization of Kigelia africana. *Biochem Cell Arch* 2025;**25**(2):1559–1570.

[163] Priya B, Menkudale A, Gahlot M, Joshi P, Agarwal M. Pharmacognostical study, phytochemical analysis and phenolic content of Kigelia africana leaves. *Int J Pharm Pharm Sci* 2013;**5**:3–6.

[164] Us A, Uh D, Ibrahim H, Bb M. A Review on African Violet Tree (Securidaca longipedunculata) : A Traditional Drug with Multiple Medicinal Uses. *Spec J Chem* 2019;**4**(3):7–14.

[165] Sani A, Ashezi NA, Abubakar US, Garba S. Phytochemical screening and antimicrobial activity of methanolic leaf extract of Securidaca longipedunculata Fresen (Polygalaceae). *J Clin Basic Res* 2023;**3**(2):921–932.

[166] Hassan LG, Umar KJ, Yusuf AJ, Rabah AB, Umar AU. Comparative Antimicrobial Activity of Securidaca longipedunculata (Fresen) Extracts on Selected Human Pathogens. *Saudi J Med Pharm Sci* 2024;**4929**:26–31.

[167] Dermane A, Kodjo A, Kporvie G, Kindji KP, Metowogo K. Immunomodulatory and anti-inflammatory activities of hydro-ethanolic extract of Securidaca longipedunculata Fresen leaves. *J HerbMed Pharmacol* 2024;**13**(2):280–288.

[168] Ochora DO, Kakudidi E, Namukobe J, Coghi P, Yang LJ, Mwakio EW, et al. A new benzophenone , and the antiplasmodial activities of the constituents of Securidaca longipedunculata fresen (Polygalaceae). *Nat Prod Res* 2021;**0**(0):1–9.

[169] Obasi TC, Benedec D, Hanganu D, Gheldiu M, Vlase L, Oniga I, et al. Free radical scavenging activity and total polyphenol content of Securidaca longipedunculata roots and leaves extracts. *Farmacia* 2020;**68**(1):116–120.

[170] Abdulkadir A, Marakisiyya M, Garba M, Hamza U, Hadiza R, Abdulrazak A, et al. Phytochemical Screening and Anticonvulsant Activities of Leaf Methanol Extract of Securidaca longipedunculata (Fresen). *Lapai J Appl Nat Sci* 2024;**9**(1):77–83.

[171] Shemishere B, Anyebe DA, Bashir YA, John E, Ifie J, Yahaya TO. Phytochemical Screening And Free Radical Scavenging Activities Of Methanol Leaf And Flower Extract Of Securidaca longipedunculata. *FUDMA J Sci* 2020;**4**(1):37–42.

[172] Gruber CW, Brien MO. Uteronic Plants and their Bioactive Constituents. *Planta Med* 2012;**77**(3):207–220.

[173] Wu C, Shieh T, Wang K. Quercetin , a main flavonoid in onion , inhibits the PGF 2 α -induced uterine contraction in vitro and in vivo. *J Funct Foods* 2015;**19**:495–504.

[174] Carreiro N, Leão I, Souza L De, Pereira JC, Henrique L, Vasconcelos C, et al. Tocolytic action and underlying mechanism of galetin 3 , 6-dimethyl ether on rat uterus. *BMC Complement Altern Med* 2017;**17**:514.

[175] Bafor EE, Kupittayanant S. Medicinal plants and their agents that affect uterine contractility. *Curr Opin Psychol* 2020;**13**:20–26.

[176] Onimisi I, Ayinde BA, Ofeimun JO, Amaechina FC, Kamal N, Gruber CW, et al. Investigation of the uterine modulating activities and mechanisms of Brachystegia eurycoma Harms (Leguminosae) methanol stem bark extract and fractions. *Sci African* 2024;**25**:e02298.

Click here to access/download
Supporting Information
Supplementary data of manuscript - BCV.docx
