# Supplementary material for: Ethnomedicinal knowledge of plants used in alternative medicine as uterotonics in Lubumbashi, Haut-Katanga, Democratic Republic of Congo
Source: PLoS One. 2026 Aug 14;21(8):e0355373. doi: 10.1371/journal.pone.0355373 (PMC13475857; doi:10.1371/journal.pone.0355373)
Supplement: Supplementary file 2 [file pone.0355373.s001.docx]

**Ethnomedicinal Knowledge of plants used in Alternative Medicine as uterotonics in Lubumbashi, Haut-Katanga, Democratic Republic of Congo**.

Bashige Chiribagula Valentin ^1^*, Okusa Ndjolo Philippe^1^, Manya Mboni Henry^2^, Cedrick Shakalenga Mutombo ^2^, Mwamba Maseho Faustin^2^, Jean Claude Nkwanga^2^, Biayi Benaja Martin^2^, Sangwa Kamulete Gregoire^2^, Mbayo Kalubandika Glaubert^2^, Numbi wa Ilunga Evodie ^2^, Muhona Melman^1^, Bakari Amuri Salvius^2^, Lumbu Simbi Jean Baptiste ^3^

**1** Department of Pharmacology, Laboratory of Therapeutic Chemistry and Analysis of Natural Substances-Faculty of Pharmaceutical Sciences (UNILU), 27, avenue Kato, Commune Kampemba, Lubumbashi-DR Congo

**2** Department of Pharmacology, Laboratory of Pharmacognosy, Faculty of Pharmaceutical Sciences, University of Lubumbashi (UNILU) 27, av Kato, Commune Kampemba, Lubumbashi, DR Congo

**3** Department of Chemistry, Faculty of Sciences, University of Lubumbashi (UNILU) N°1 Maternity Avenue, Commune of Lubumbashi, DR Congo.

**Corresponding author**: Bashige Chiribagula Valentin: [bashige.chiribagula@unilu.ac.cd](mailto:bashige.chiribagula@unilu.ac.cd);

**Supplementary data**

**Supplement 1 (S1). Survey questionnaire**

### Survey questionnaire (traditional practitioners, herbalists, housewife) N°……..

NB. The interviewer must not leave the questionnaire with the respondent, but must complete it himself during the interviews after informed consent. The reverse side can be used to complete the information on the plants.

**A. Identity of resource person**
1. Names:………………………………………………………. Phone Number :……………………………
2. Gender : M	F
3. Age : a. 18-27 b. 28-37 c. 38-47 d. 48-57 e. +58 (If age is known, it's best to specify it) ………..
4. Ethnicity : ……. Adress : sector (Municipality), district : .………………………………………………………………………………………….……………………**....**
5. Education level
a) None	b) Primary	c) Secondary	d) Superior	e) Professional	f) Other ……………………

**B. Ethnomedicinal knowledge**
6. Are you? : a) Traditional healer	b) devin	c) Other ………………………………………………………………
7. When did you become a TH (Herbalist) (or medicinal plant user)?................................................................
8. How did you become a TH (herbalist) and by what means?
a) Initiation (learning) b) Dream c) legacy d) curiosity f) other …………………………………………….
9. Do you know what uterotonic is? a. Yes	b. No

10. Have you ever treated people who have difficulty giving birth using plants? a) Yes	b) No	c. number of patients: ………./ month d. Number of patients received one month prior to this interview (in each case, specify age) ? ……. c. What type of childbirth complications do you treat? .............................

11. How do you ensure that the person is experiencing difficulty giving birth due to a lack of uterine muscle contractions? …...........................................................................................................................................................................................

12. What are the consequences of the uterine muscle failing to contract during childbirth?

……………………………………………………………………………………………………………………………………………………..

13. Based on your experience, what are the causes of uterine muscle contraction failure during childbirth?

……………………………………………………………………………………………………………..

14. What plants do you use to correct uterine muscle contraction defects during childbirth?

| **NVr (ethnicity)** | **PU** | **MoRe** | **CC** | **PatS** | **Hab** | **Nsc** | **PoS** | **TCD** |
| --- | --- | --- | --- | --- | --- | --- | --- | --- |
|  |  |  |  |  |  |  |  |  |

NVr: vernacular name and origin; PU: part used; MoRe /time of harvesting; CC: storage condition; PatS: pathology treated or indication; Hab: habitat and/or location; NSc: scientific name; PoS: dosage; THe: Type of childbirth difficulty.
15. What recipes are used as uterotonic in combination of herbs?

| **NVr (ethnicity)** | **PU** | **MoRe** | **CC** | **PatS** | **Hab** | **Nsc** | **PoS** | **TCD** |
| --- | --- | --- | --- | --- | --- | --- | --- | --- |
|  |  |  |  |  |  |  |  |  |

NVr: vernacular name and origin; PU: part used; MoRe /time of harvest; CC: storage condition; PatS: pathology treated or indication; Hab: habitat and/or location; NSc: scientific name.

**We certify that the translation has been made in accordance with the primary information provided in Swahili.**
