# Supplementary material for: Ethnomedicinal knowledge of plants used in alternative medicine as uterotonics in Lubumbashi, Haut-Katanga, Democratic Republic of Congo
Source: PLoS One. 2026 Aug 14;21(8):e0355373. doi: 10.1371/journal.pone.0355373 (PMC13475857; doi:10.1371/journal.pone.0355373)
Supplement: Supplementary file 3 [file pone.0355373.s002.docx]

**Ethnomedicinal Knowledge of plants used in Alternative Medicine as uterotonics in Lubumbashi, Haut-Katanga, Democratic Republic of Congo**.

Bashige Chiribagula Valentin ^1^*, Okusa Ndjolo Philippe^1^, Manya Mboni Henry^2^, Cedrick Shakalenga Mutombo ^2^, Mwamba Maseho Faustin^2^, Jean Claude Nkwanga^2^, Biayi Benaja Martin^2^, Sangwa Kamulete Gregoire^2^, Mbayo Kalubandika Glaubert^2^, Numbi wa Ilunga Evodie ^2^, Muhona Melman^1^, ,Bakari Amuri Salvius^2^, Lumbu Simbi Jean Baptiste ^3^

**1** Department of Pharmacology, Laboratory of Therapeutic Chemistry and Analysis of Natural Substances-Faculty of Pharmaceutical Sciences (UNILU), 27, avenue Kato, Commune Kampemba, Lubumbashi-DR Congo

**2** Department of Pharmacology, Laboratory of Pharmacognosy, Faculty of Pharmaceutical Sciences, University of Lubumbashi (UNILU) 27, av Kato, Commune Kampemba, Lubumbashi, DR Congo

**3** Department of Chemistry, Faculty of Sciences, University of Lubumbashi (UNILU) N°1 Maternity Avenue, Commune of Lubumbashi, DR Congo.

**Corresponding author**: Bashige Chiribagula Valentin: [bashige.chiribagula@unilu.ac.cd](mailto:bashige.chiribagula@unilu.ac.cd);

## **Graphical Abstract**

**The map in the graphical abstract was created using the free software QGIS version 3.22.14 (available at: www.qgis.org). It was then edited, like the other images, using Microsoft Paint.**
