# Supplementary material for: Ethnomedicinal knowledge of plants used in alternative medicine as uterotonics in Lubumbashi, Haut-Katanga, Democratic Republic of Congo
Source: PLoS One. 2026 Aug 14;21(8):e0355373. doi: 10.1371/journal.pone.0355373 (PMC13475857; doi:10.1371/journal.pone.0355373)
Supplement: Supplementary file 4 [file pone.0355373.s005.docx]

**Dear Editor,**

Please find below our reaction to the various comments made by the reviewers of our manuscript.

**Manuscript number:** PONE-D-26-11446

**Title:** Ethnomedicinal Knowledge of plants used in Alternative Medicine as uterotonics in Lubumbashi, Haut-Katanga, Democratic Republic of Congo

**Article type**: Research Paper

**Corresponding Author**: Prof Bashige Chiribagula Valentin, [bashige.chiribagula@unilu.ac.cd](mailto:bashige.chiribagula@unilu.ac.cd) (BCV)

**Responses to reviewers' comments**

**Academic editor**

1. **Please ensure that your manuscript meets PLOS ONE's style requirements, including those for file naming**.

We have followed the format suggested in the guidelines.

2. **In the ethics statement in the Methods, you have specified that verbal consent was obtained. Please provide additional details regarding how this consent was documented and witnessed, and state whether this was approved by the IRB.**

Among traditional practitioners, consent was obtained in the presence of the provincial president of UNAGCO (National Union of Healers of the Congo). Among herbalists, consent was obtained in the presence of the market administrators where they were met. In households, women who had experienced a difficult childbirth gave their consent in the presence of an influential member of the avenue (street) where they live or the avenue chief, as applicable. It goes without saying that without consent, no interviews would have been possible. The consent was approved by the Medical Ethics Committee of the University of Lubumbashi through approval of the experimental protocol under number: UNILU/CEM/035/2024. As we stated in the “Ethical Considerations” section (Lines 170-171).

3. **Your ethics statement should only appear in the Methods section of your manuscript. If your ethics statement is written in any section besides the Methods, please delete it from any other section**.

The verification was performed, and no issues were found.

4. **Request for permission to publish a map of the city of Lubumbashi (Fig 1.)**

Regarding the image in Figure 1: I can assure you that this figure was generated by our research team using QGIS software. It is therefore not subject to any copyright.

5. **Request for permission to publish the photos shown in the figure 5.**

This is Figure 5, for which you are requesting permission to publish from the authors. It was created using photos taken in the field during our field trips to the INERA station in Kipopo. It is therefore not subject to any copyright. It is possible that the photos resemble preexisting designs. This could be because we take photos to help us identify plants, and as a result, they may show similarities to preexisting databases (Lines 447).

**Reviewer #2**

1. **When abbreviating The Democratic republic of Congo, you alternatively used DR Congo and DRC. Please, choose one.**

The term “DRC” has been selected and standardized.

2. **Modify title as ‘Ethnomedicinal Knowledge of plants used in Alternative Medicine as uterotonics in Lubumbashi, Haut-Katanga, Democratic Republic of Congo’.**

The title was changed to spell out “DRC” in full.

3. **Lines 43 and 44: Replace ‘Democratic Republic of Congo; with ‘Democratic Republic of Congo (DRC)’ or ‘Democratic Republic of Congo (DR Congo)’ as you choose.**

The requested replacement has been made.

4. **Lines 73-76 : Modify statement as ‘These complications which are common due to socioeconomic inequalities, poverty, limited access to healthcare facilities and a shortage of qualified staff, particularly in rural areas [4,5], could be prevented with rapid access to quality care [5]’.**

The sentence has been revised as suggested (Lines 67-70).

5. **Lines 86-90: Rewrite statement as ‘A review of the literature from 1980 to 2010 identified 280 African oxytocic plants [13], while another reported 16 plants [14] of which the following demonstrated experimental uterotonic activity: Heimia salicifolia Link, Lythraceae [15], the roots of Azanza garckeana (F. Hoffm.) Exell & Hillc., a synonym for Thespesia garckeana F. Hoffm, Malvaceae [16] and the stem bark of Viburnum opulus L.,Viburnaceae [17]’.**

The sentence has been revised as suggested (Lines 81-84).

6. **Line 98 : I am not clear with the phrase ‘and constituting the present experimental context’.**

The sentence has been reworded to incorporate your comment (Lines 91-93).

7. **Line 103 : Replace the phrase ‘This study aims to’ with ‘This study aimed to identify and document’.**

The sentence has been revised based on your suggestion (Lines 97-98).

8. **Line 108 : Is the total number of communities in Lubumbashi seven. If they are more than seven, what sampling method did you use to sample these seven communities?**

Lubumbashi is home to several communities. In the sentence you are referring to, it is not a single community but rather the communes. The city of Lubumbashi actually has 7 communes (Lines 102-103).

9. **Line 122 : Replace ‘informants,’ with ‘informants comprising’.**

The replacement has been completed (Line 117).

10. **Line 123 : Need for provision of a brief description here of the difference between herbalists and traditional healers.**

There is, of course, a difference between herbalists and traditional healers. Herbalists use medicinal plants solely for commercial purposes, whereas traditional healers use natural resources—guided by tradition—to heal. They do not necessarily work for therapeutic purposes, unlike herbalists, who sell plants commercially (Lines 280-284).

11. **Line 124 : I do not think a structured questionnaire is suitable for open-ended interviews. It is rather employed for a close-ended interview.**

The questionnaire was semi-structured. We revised the text to reflect this observation (Line 119).

12. **Line 136 : Please, state the sampling method you used to recruit the 36 herbalists for interviews ?**

In response to this question, we have reworded this paragraph as follows : « The herbalists included in this study were recruited from six major traditional medicine markets in Lubumbashi (Mzee, Kenya, Zambia, Kamalondo, Kalala Banza, and Moise), which serve as dynamic hubs of ethnobotanical knowledge and living plant traditions. We approached all herbalists encountered in these markets who consented to participate. In total, thirty-six practitioners were interviewed. Through direct interviews, they shared the therapeutic recipes they employ, revealing that twenty-six (72%) reported at least one specific uterotonic herbal formulation. » (Lines 129-134).

13. **Lines 140-143 : According to the statement, the total number of traditional healers included in this study should be 77, not 78. [45 + (33-1)] = 77. Please check. Additionally, why did you stopped at 33 healers during the snowball sampling ?**

That’s not actually the correct calculation; the “traditional healers” we found comes from the list of 50, not the list of 45, so the correct calculation is 45 + 32 + 1 = 78. We didn’t intentionally arrive at 33, it’s simply the result of our snowball sampling. It doesn’t depend on us, but on the informants.

14. **Line 162 : Replace ‘significant species’ with ‘significance of the reported species’.**

The replacement has been completed.

15. **Lines 172-174 : This statement needs rephrasing for better clarity.**

In response to your comment, this paragraph has been reworded as follows: In this study, the UV index is used to assess the medicinal importance of a plant in the study area; the RCF index measures a plant’s popularity among people who use it as an uterotonic. This also implies a consensus among informants regarding its use to stimulate uterine contractions during childbirth.

16. **Line 186: Replace ‘surveys of’ with ‘surveys of medicinal plants of’.**

The parenthetical note was added.

17. **Line 192 (Table 1) : Modify the title of the table as ‘General information of plants used as uterrotonic agents in Lubumbashi’.**

The title has been changed as you suggested (Line 186).

18. **Line 192 (Table 1): There is too much use of symbols and abbreviations in this table. Thus, try to reduce them for the sake of clarity. It is a bit awkward for a reader to often go back and forth between the table and the legend to understand the meaning of the symbols and abbreviations. Additionally, I am not sure whether every given abbreviation or symbol has been explained in the legend. Throughout the list of scientific names, please also carefully check whether abbreviated author names need periods or not**.

The formatting of abbreviations follows the standard used in the field. It was preferred by a panel of consulted experts—colleagues in ethnopharmacology—during a preliminary trial, as it had the advantage of allowing the data to be condensed in portrait orientation rather than landscape. As for scientific names, as we mentioned in the methodology section, they have been formatted according to the POWO platform, unless you have a specific comment regarding a particular species.

19. **Line 214 (heading 3.1.1): Modify title as ‘Taxonomic diversity of medicinal plants recorded’.**

The title has been changed as suggested (Line 208).

20. **Line 227 (Figure 2): replace caption as ‘Relative citation frequencies of plant families with n ≥ 2 plants.**

The figure caption has been changed as suggested (Line 214).

21. **Lines 229 and 230 : For better clarity, please use a separate caption for each of the three figures.**

That is a good suggestion; however, it has the drawback of increasing the number of illustrations for a non-sequential presentation. Without compromising our goal of keeping the number of figures to a minimum, and to address your concern, we have reorganized the figure.

22. **Line 241: What do you mean by the phrase ‘18 recipes and 3’? Please modify.**

We have made the changes based on your suggestion.

23. **Line 247 (Figure 4): In the figure caption, please, rephrase the phrase ‘facilitate childbirth formulations’ for sake of clarity.**

We have replaced this term with “uterotonic formulation. (Line 241)

24. **Line 264 (Table 3): In the title of the table, replace ‘othe’ with ‘other’.**

The title has been changed (Line 258).

25. **Line 267 (heading 3.4): Replace ‘survey’ with ‘surveyed’.**

The title has been changed as suggested (Line 261).

26. **Line 268: Replace ‘relative citation frequency’ with ‘RCF’.**

The replacement has been completed (Line 262).

27. **Line 274 (heading 3.4): This heading should be put at the beginning of the ‘Results’ section.**

The results section can only begin with the overall table, from which all the other aspects highlighted in the text are derived. It is for this reason that we did not begin with the ethnobotanical indices, even though, to a certain extent, they emphasize specific findings.

**Reviewer #3**

1. **Line 103-104: The word invent is not the right choice of word. Authors are not inventing anything here rather they are documenting the traditional knowledge of ................**

The sentence was revised as follows: “This study aimed to identify medicinal plants and recipes with uterotonic effects used in traditional medicine in Lubumbashi to facilitate childbirth.”(Lines 97-98)

2. **Line 154 : remove google it is not a bibliographic databases.**

In response to your request, Google has been removed (Line 156), provided that, for the purposes of this study, all the studies we found on Google were also found on Google Scholar. However, it should be noted that nowadays, there are peer-reviewed journals that are indexed on Google. It would therefore be incomplete not to take a look there to see what is published there (Line 150).

3. **Table 1. The first two plant species does not have herbarium voucher specimen number.**

The herbarium codes have been added for both plants (Table 1).

4. **Table 1. R5, R10 16, R21, and many more are empty.**

That’s a good point. However, this situation is perfectly normal. It stems from the fact that these recipes differ from the preceding ones only in the information listed in this column—namely, the part of the plant used and the method of preparation. This is because a single plant can have multiple medicinal uses, particularly when different parts of the plant are used.
