# Supplementary material for: Ethnomedicinal knowledge of plants used in alternative medicine as uterotonics in Lubumbashi, Haut-Katanga, Democratic Republic of Congo
Source: PLoS One. 2026 Aug 14;21(8):e0355373. doi: 10.1371/journal.pone.0355373 (PMC13475857; doi:10.1371/journal.pone.0355373)
Supplement: Peer review history

# Peer review: Decision Letter 0

**DOI:** 10.1371/journal.pone.0355373.r001

**Authors:** Ramesh Kumar Ahirwar

2 Jul 2026

Dear Dr. Chiribagula,

Thank you for submitting your manuscript to PLOS ONE. After careful consideration, we feel that it has merit but does not fully meet PLOS ONE’s publication criteria as it currently stands. Therefore, we invite you to submit a revised version of the manuscript that addresses the points raised during the review process.

Please submit your revised manuscript by Aug 16 2026 11:59PM. If you will need more time than this to complete your revisions, please reply to this message or contact the journal office at plosone@plos.org. When you're ready to submit your revision, log on to https://www.editorialmanager.com/pone/ and select the 'Submissions Needing Revision' folder to locate your manuscript file.

- A letter that responds to each point raised by the academic editor and reviewer(s). You should upload this letter as a separate file labeled 'Response to Reviewers'.
- A marked-up copy of your manuscript that highlights changes made to the original version. You should upload this as a separate file labeled 'Revised Manuscript with Track Changes'.
- An unmarked version of your revised paper without tracked changes. You should upload this as a separate file labeled 'Manuscript'.

If you would like to make changes to your financial disclosure, please include your updated statement in your cover letter. Guidelines for resubmitting your figure files are available below the reviewer comments at the end of this letter.

If applicable, we recommend that you deposit your laboratory protocols in protocols.io to enhance the reproducibility of your results. Protocols.io assigns your protocol its own identifier (DOI) so that it can be cited independently in the future. For instructions see: https://journals.plos.org/plosone/s/submission-guidelines#loc-laboratory-protocols. Additionally, PLOS ONE offers an option for publishing peer-reviewed Lab Protocol articles, which describe protocols hosted on protocols.io. Read more information on sharing protocols at https://plos.org/protocols?utm_medium=editorial-email&utm_source=authorletters&utm_campaign=protocols.

As the corresponding author, your ORCID iD is verified in the submission system and will appear in the published article. PLOS supports the use of ORCID, and we encourage all coauthors to register for an ORCID iD and use it as well. Please encourage your coauthors to verify their ORCID iD within the submission system before final acceptance, as unverified ORCID iDs will not appear in the published article. *Only*  the individual author can complete the verification step; PLOS staff *cannot*  verify ORCID iDs on behalf of authors.

We look forward to receiving your revised manuscript.

Kind regards,

Ramesh Kumar Ahirwar, Ph.D.

Academic Editor

PLOS One

Journal Requirements:

When submitting your revision, we need you to address these additional requirements.

1. Please ensure that your manuscript meets PLOS ONE's style requirements, including those for file naming. The PLOS ONE style templates can be found at

https://journals.plos.org/plosone/s/file?id=wjVg/PLOSOne_formatting_sample_main_body.pdf and https://journals.plos.org/plosone/s/file?id=ba62/PLOSOne_formatting_sample_title_authors_affiliations.pdf

2. In the ethics statement in the Methods, you have specified that verbal consent was obtained. Please provide additional details regarding how this consent was documented and witnessed, and state whether this was approved by the IRB.

3. Your ethics statement should only appear in the Methods section of your manuscript. If your ethics statement is written in any section besides the Methods, please delete it from any other section.

4. We note that Figure 1,in your submission contain [map/satellite] images which may be copyrighted. All PLOS content is published under the Creative Commons Attribution License (CC BY 4.0), which means that the manuscript, images, and Supporting Information files will be freely available online, and any third party is permitted to access, download, copy, distribute, and use these materials in any way, even commercially, with proper attribution. For these reasons, we cannot publish previously copyrighted maps or satellite images created using proprietary data, such as Google software (Google Maps, Street View, and Earth). For more information, see our copyright guidelines: http://journals.plos.org/plosone/s/licenses-and-copyright.

We require you to either (1) present written permission from the copyright holder to publish these figures specifically under the CC BY 4.0 license, or (2) remove the figures from your submission:

a. You may seek permission from the original copyright holder of Figure 1, to publish the content specifically under the CC BY 4.0 license.

We recommend that you contact the original copyright holder with the Content Permission Form (http://journals.plos.org/plosone/s/file?id=7c09/content-permission-form.pdf) and the following text:

“I request permission for the open-access journal PLOS ONE to publish XXX under the Creative Commons Attribution License (CCAL) CC BY 4.0 (http://creativecommons.org/licenses/by/4.0/). Please be aware that this license allows unrestricted use and distribution, even commercially, by third parties. Please reply and provide explicit written permission to publish XXX under a CC BY license and complete the attached form.”

Please upload the completed Content Permission Form or other proof of granted permissions as an "Other" file with your submission.

In the figure caption of the copyrighted figure, please include the following text: “Reprinted from [ref] under a CC BY license, with permission from [name of publisher], original copyright [original copyright year].”

b. If you are unable to obtain permission from the original copyright holder to publish these figures under the CC BY 4.0 license or if the copyright holder’s requirements are incompatible with the CC BY 4.0 license, please either i) remove the figure or ii) supply a replacement figure that complies with the CC BY 4.0 license. Please check copyright information on all replacement figures and update the figure caption with source information. If applicable, please specify in the figure caption text when a figure is similar but not identical to the original image and is therefore for illustrative purposes only.

The following resources for replacing copyrighted map figures may be helpful:

USGS National Map Viewer (public domain): http://viewer.nationalmap.gov/viewer/

The Gateway to Astronaut Photography of Earth (public domain): http://eol.jsc.nasa.gov/sseop/clickmap/

Maps at the CIA (public domain): https://www.cia.gov/library/publications/the-world-factbook/index.html and https://www.cia.gov/library/publications/cia-maps-publications/index.html

NASA Earth Observatory (public domain): http://earthobservatory.nasa.gov/

Landsat: http://landsat.visibleearth.nasa.gov/

USGS EROS (Earth Resources Observatory and Science (EROS) Center) (public domain): http://eros.usgs.gov/#

Natural Earth (public domain): http://www.naturalearthdata.com/

5. We note that Figure 5 in your submission contain copyrighted images. All PLOS content is published under the Creative Commons Attribution License (CC BY 4.0), which means that the manuscript, images, and Supporting Information files will be freely available online, and any third party is permitted to access, download, copy, distribute, and use these materials in any way, even commercially, with proper attribution. For more information, see our copyright guidelines: http://journals.plos.org/plosone/s/licenses-and-copyright.

We require you to either (1) present written permission from the copyright holder to publish these figures specifically under the CC BY 4.0 license, or (2) remove the figures from your submission:

a. You may seek permission from the original copyright holder of Figure 5 to publish the content specifically under the CC BY 4.0 license.

We recommend that you contact the original copyright holder with the Content Permission Form (http://journals.plos.org/plosone/s/file?id=7c09/content-permission-form.pdf) and the following text:

“I request permission for the open-access journal PLOS ONE to publish XXX under the Creative Commons Attribution License (CCAL) CC BY 4.0 (http://creativecommons.org/licenses/by/4.0/). Please be aware that this license allows unrestricted use and distribution, even commercially, by third parties. Please reply and provide explicit written permission to publish XXX under a CC BY license and complete the attached form.”

Please upload the completed Content Permission Form or other proof of granted permissions as an "Other" file with your submission.

In the figure caption of the copyrighted figure, please include the following text: “Reprinted from [ref] under a CC BY license, with permission from [name of publisher], original copyright [original copyright year].”

b. If you are unable to obtain permission from the original copyright holder to publish these figures under the CC BY 4.0 license or if the copyright holder’s requirements are incompatible with the CC BY 4.0 license, please either i) remove the figure or ii) supply a replacement figure that complies with the CC BY 4.0 license. Please check copyright information on all replacement figures and update the figure caption with source information. If applicable, please specify in the figure caption text when a figure is similar but not identical to the original image and is therefore for illustrative purposes only.

6. Please include captions for your Supporting Information files at the end of your manuscript, and update any in-text citations to match accordingly. Please see our Supporting Information guidelines for more information: http://journals.plos.org/plosone/s/supporting-information.

7. If the reviewer comments include a recommendation to cite specific previously published works, please review and evaluate these publications to determine whether they are relevant and should be cited. There is no requirement to cite these works unless the editor has indicated otherwise.

[Note: HTML markup is below. Please do not edit.]

Reviewers' comments:

Reviewer's Responses to Questions

**Comments to the Author**

1. Is the manuscript technically sound, and do the data support the conclusions?

Reviewer #1: Yes

Reviewer #2: Yes

Reviewer #3: Yes

**********

2. Has the statistical analysis been performed appropriately and rigorously?

Reviewer #1: Yes

Reviewer #2: N/A

Reviewer #3: Yes

**********

3. Have the authors made all data underlying the findings in their manuscript fully available?

The PLOS Data policy

Reviewer #1: Yes

Reviewer #2: Yes

Reviewer #3: Yes

**********

4. Is the manuscript presented in an intelligible fashion and written in standard English?

Reviewer #1: Yes

Reviewer #2: Yes

Reviewer #3: Yes

**********

Reviewer #1: We commend and encourage the authors for this very interesting study, which contributes to the valorization of traditional Congolese medicine (DRC). Several studies in this region have shown that a large proportion of the population relies on traditional medicine (medicinal plants) for healthcare. Plants used in the management of childbirth have not been sufficiently studied; thus, the authors have made a valuable contribution to this field of research.

Reviewer #2: Dear Editor,

I went through the manuscript titled ‘Ethnomedicinal Knowledge of plants used in Alternative Medicine as uterotonic in Lubumbashi, Haut-Katanga (DR Congo)’. Each line number mentioned below is based on the manuscript in PDF format.

General comments

- The whole document needs polishing for better clarity and ease of reading.

- Throughout the manuscript, avoid using full stops at the end of the title, headings and subheadings.

- When abbreviating The Democratic republic of Congo, you alternatively used DR Congo and DRC. Please, choose one.

Section by section comments

Title

- Modify title as ‘Ethnomedicinal Knowledge of plants used in Alternative Medicine as uterotonics in Lubumbashi, Haut-Katanga, Democratic Republic of Congo’.

Abstract

- Lines 43 and 44: Replace ‘Democratic Republic of Congo; with ‘Democratic Republic of Congo (DRC)’ or ‘Democratic Republic of Congo (DR Congo)’ as you choose.

1. Introduction

- Lines 73-76: Modify statement as ‘These complications which are common due to socioeconomic inequalities, poverty, limited access to healthcare facilities and a shortage of qualified staff, particularly in rural areas [4,5], could be prevented with rapid access to quality care [5]’.

- Lines 86-90: Rewrite statement as ‘A review of the literature from 1980 to 2010 identified 280 African oxytocic plants [13], while another reported 16 plants [14] of which the following demonstrated experimental uterotonic activity: Heimia salicifolia Link, Lythraceae [15], the roots of Azanza garckeana (F. Hoffm.) Exell & Hillc., a synonym for Thespesia garckeana F. Hoffm, Malvaceae [16] and the stem bark of Viburnum opulus L.,Viburnaceae [17]’.

- Line 98: I am not clear with the phrase ‘and constituting the present experimental context’.

- Line 103: Replace the phrase ‘This study aims to’ with ‘This study aimed to identify and document’.

Materials and methods

- Line 108: Is the total number of communities in Lubumbashi seven. If they are more than seven, what sampling method did you use to sample these seven communities?

- Line 122: Replace ‘informants,’ with ‘informants comprising’.

- Line 123: Need for provision of a brief description here of the difference between herbalists and traditional healers.

- Line 124: I do not think a structured questionnaire is suitable for open-ended interviews. It is rather employed for a close-ended interview.

- Line 136: Please, state the sampling method you used to recruit the 36 herbalists for interviews?

- Lines 140-143: According to the statement, the total number of traditional healers included in this study should be 77, not 78. [45 + (33-1)] = 77. Please check. Additionally, why did you stopped at 33 healers during the snowball sampling?

- Line 162: Replace ‘significant species’ with ‘significance of the reported species’.

- Lines 172-174: This statement needs rephrasing for better clarity.

Results

- Line 186: Replace ‘surveys of’ with ‘surveys of medicinal plants of’.

- Line 192 (Table 1): Modify the title of the table as ‘General information of plants used as uterrotonic agents in Lubumbashi’.

- Line 192 (Table 1): There is too much use of symbols and abbreviations in this table. Thus, try to reduce them for the sake of clarity. It is a bit awkward for a reader to often go back and forth between the table and the legend to understand the meaning of the symbols and abbreviations. Additionally, I am not sure whether every given abbreviation or symbol has been explained in the legend. Throughout the list of scientific names, please also carefully check whether abbreviated author names need periods or not.

- Line 214 (heading 3.1.1): Modify title as ‘Taxonomic diversity of medicinal plants recorded’.

- Line 227 (Figure 2): replace caption as ‘Relative citation frequencies of plant families with n ≥ 2 plants.

- Lines 229 and 230: For better clarity, please use a separate caption for each of the three figures.

- Line 241: What do you mean by the phrase ‘18 recipes and 3’? Please modify.

- Line 247 (Figure 4): In the figure caption, please, rephrase the phrase ‘facilitate childbirth formulations’ for sake of clarity.

- Line 264 (Table 3): In the title of the table, replace ‘othe’ with ‘other’.

- Line 267 (heading 3.4): Replace ‘survey’ with ‘surveyed’.

- Line 268: Replace ‘relative citation frequency’ with ‘RCF’.

- Line 274 (heading 3.4): This heading should be put at the beginning of the ‘Results’ section.

Reviewer #3: The research article "Ethnomedicinal Knowledge of plants used in Alternative Medicine as uterotonic in

Lubumbashi, Haut-Katanga (DR Congo)." is interesting and can be accepted for publication after a few minor correction

Comments

1.) Line 103-104: The word invent is not the right choice of word. Authors are not inventing anything here rather they are documenting the traditional knowledge of ................

2.) Line 154: remove google it is not a bibliographic databases

3.) Table 1.: The first two plant species does not have herbarium voucher specimen number.

4.) Table 1.: R5, R10 16, R21, and many more are empty.

**********

what does this mean?). If published, this will include your full peer review and any attached files.

If you choose “no”, your identity will remain anonymous but your review may still be made public.

**Do you want your identity to be public for this peer review?**  For information about this choice, including consent withdrawal, please see our Privacy Policy

Reviewer #1: **Yes:** Gaël Nzuzi Mavungu

Reviewer #2: No

Reviewer #3: No

**********

[NOTE: If reviewer comments were submitted as an attachment file, they will be attached to this email and accessible via the submission site. Please log into your account, locate the manuscript record, and check for the action link "View Attachments". If this link does not appear, there are no attachment files.]

To ensure your figures meet our technical requirements, please review our figure guidelines: https://journals.plos.org/plosone/s/figures

You may also use PLOS’s free figure tool, NAAS, to help you prepare publication quality figures: https://journals.plos.org/plosone/s/figures#loc-tools-for-figure-preparation.

NAAS will assess whether your figures meet our technical requirements by comparing each figure against our figure specifications.

Attachment Submitted filename: PONE-D-26-11446.pdf

# Author comment: Author response to Decision Letter 1

**DOI:** 10.1371/journal.pone.0355373.r002

8 Jul 2026

Dear Editor,

Please find below our reaction to the various comments made by the reviewers of our manuscript.

Responses to reviewers' comments

Academic editor

1. Please ensure that your manuscript meets PLOS ONE's style requirements, including those for file naming.

We have followed the format suggested in the guidelines.

2. In the ethics statement in the Methods, you have specified that verbal consent was obtained. Please provide additional details regarding how this consent was documented and witnessed, and state whether this was approved by the IRB.

Among traditional practitioners, consent was obtained in the presence of the provincial president of UNAGCO (National Union of Healers of the Congo). Among herbalists, consent was obtained in the presence of the market administrators where they were met. In households, women who had experienced a difficult childbirth gave their consent in the presence of an influential member of the avenue (street) where they live or the avenue chief, as applicable. It goes without saying that without consent, no interviews would have been possible. The consent was approved by the Medical Ethics Committee of the University of Lubumbashi through approval of the experimental protocol under number: UNILU/CEM/035/2024. As we stated in the “Ethical Considerations” section (Lines 170-171).

3. Your ethics statement should only appear in the Methods section of your manuscript. If your ethics statement is written in any section besides the Methods, please delete it from any other section.

The verification was performed, and no issues were found.

4. Request for permission to publish a map of the city of Lubumbashi (Fig 1.)

Regarding the image in Figure 1: I can assure you that this figure was generated by our research team using QGIS software. It is therefore not subject to any copyright.

5. Request for permission to publish the photos shown in the figure 5.

This is Figure 5, for which you are requesting permission to publish from the authors. It was created using photos taken in the field during our field trips to the INERA station in Kipopo. It is therefore not subject to any copyright. It is possible that the photos resemble preexisting designs. This could be because we take photos to help us identify plants, and as a result, they may show similarities to preexisting databases (Lines 447).

Reviewer #2

1. When abbreviating The Democratic republic of Congo, you alternatively used DR Congo and DRC. Please, choose one.

The term “DRC” has been selected and standardized.

2. Modify title as ‘Ethnomedicinal Knowledge of plants used in Alternative Medicine as uterotonics in Lubumbashi, Haut-Katanga, Democratic Republic of Congo’.

The title was changed to spell out “DRC” in full.

3. Lines 43 and 44: Replace ‘Democratic Republic of Congo; with ‘Democratic Republic of Congo (DRC)’ or ‘Democratic Republic of Congo (DR Congo)’ as you choose.

The requested replacement has been made.

4. Lines 73-76 : Modify statement as ‘These complications which are common due to socioeconomic inequalities, poverty, limited access to healthcare facilities and a shortage of qualified staff, particularly in rural areas [4,5], could be prevented with rapid access to quality care [5]’.

The sentence has been revised as suggested (Lines 67-70).

5. Lines 86-90: Rewrite statement as ‘A review of the literature from 1980 to 2010 identified 280 African oxytocic plants [13], while another reported 16 plants [14] of which the following demonstrated experimental uterotonic activity: Heimia salicifolia Link, Lythraceae [15], the roots of Azanza garckeana (F. Hoffm.) Exell & Hillc., a synonym for Thespesia garckeana F. Hoffm, Malvaceae [16] and the stem bark of Viburnum opulus L.,Viburnaceae [17]’.

The sentence has been revised as suggested (Lines 81-84).

6. Line 98 : I am not clear with the phrase ‘and constituting the present experimental context’.

The sentence has been reworded to incorporate your comment (Lines 91-93).

7. Line 103 : Replace the phrase ‘This study aims to’ with ‘This study aimed to identify and document’.

The sentence has been revised based on your suggestion (Lines 97-98).

8. Line 108 : Is the total number of communities in Lubumbashi seven. If they are more than seven, what sampling method did you use to sample these seven communities?

Lubumbashi is home to several communities. In the sentence you are referring to, it is not a single community but rather the communes. The city of Lubumbashi actually has 7 communes (Lines 102-103).

9. Line 122 : Replace ‘informants,’ with ‘informants comprising’.

The replacement has been completed (Line 117).

10. Line 123 : Need for provision of a brief description here of the difference between herbalists and traditional healers.

There is, of course, a difference between herbalists and traditional healers. Herbalists use medicinal plants solely for commercial purposes, whereas traditional healers use natural resources—guided by tradition—to heal. They do not necessarily work for therapeutic purposes, unlike herbalists, who sell plants commercially (Lines 280-284).

11. Line 124 : I do not think a structured questionnaire is suitable for open-ended interviews. It is rather employed for a close-ended interview.

The questionnaire was semi-structured. We revised the text to reflect this observation (Line 119).

12. Line 136 : Please, state the sampling method you used to recruit the 36 herbalists for interviews ?

In response to this question, we have reworded this paragraph as follows : « The herbalists included in this study were recruited from six major traditional medicine markets in Lubumbashi (Mzee, Kenya, Zambia, Kamalondo, Kalala Banza, and Moise), which serve as dynamic hubs of ethnobotanical knowledge and living plant traditions. We approached all herbalists encountered in these markets who consented to participate. In total, thirty-six practitioners were interviewed. Through direct interviews, they shared the therapeutic recipes they employ, revealing that twenty-six (72%) reported at least one specific uterotonic herbal formulation. » (Lines 129-134).

13. Lines 140-143 : According to the statement, the total number of traditional healers included in this study should be 77, not 78. [45 + (33-1)] = 77. Please check. Additionally, why did you stopped at 33 healers during the snowball sampling ?

That’s not actually the correct calculation; the “traditional healers” we found comes from the list of 50, not the list of 45, so the correct calculation is 45 + 32 + 1 = 78. We didn’t intentionally arrive at 33, it’s simply the result of our snowball sampling. It doesn’t depend on us, but on the informants.

14. Line 162 : Replace ‘significant species’ with ‘significance of the reported species’.

The replacement has been completed.

15. Lines 172-174 : This statement needs rephrasing for better clarity.

In response to your comment, this paragraph has been reworded as follows: In this study, the UV index is used to assess the medicinal importance of a plant in the study area; the RCF index measures a plant’s popularity among people who use it as an uterotonic. This also implies a consensus among informants regarding its use to stimulate uterine contractions during childbirth.

16. Line 186: Replace ‘surveys of’ with ‘surveys of medicinal plants of’.

The parenthetical note was added.

17. Line 192 (Table 1) : Modify the title of the table as ‘General information of plants used as uterrotonic agents in Lubumbashi’.

The title has been changed as you suggested (Line 186).

18. Line 192 (Table 1): There is too much use of symbols and abbreviations in this table. Thus, try to reduce them for the sake of clarity. It is a bit awkward for a reader to often go back and forth between the table and the legend to understand the meaning of the symbols and abbreviations. Additionally, I am not sure whether every given abbreviation or symbol has been explained in the legend. Throughout the list of scientific names, please also carefully check whether abbreviated author names need periods or not.

The formatting of abbreviations follows the standard used in the field. It was preferred by a panel of consulted experts—colleagues in ethnopharmacology—during a preliminary trial, as it had the advantage of allowing the data to be condensed in portrait orientation rather than landscape. As for scientific names, as we mentioned in the methodology section, they have been formatted according to the POWO platform, unless you have a specific comment regarding a particular species.

19. Line 214 (heading 3.1.1): Modify title as ‘Taxonomic diversity of medicinal plants recorded’.

The title has been changed as suggested (Line 208).

20. Line 227 (Figure 2): replace caption as ‘Relative citation frequencies of plant families with n ≥ 2 plants.

The figure caption has been changed as suggested (Line 214).

21. Lines 229 and 230 : For better clarity, please use a separate caption for each of the three figures.

That is a good suggestion; however, it has the drawback of increasing the number of illustrations for a non-sequential presentation. Without compromising our goal of keeping the number of figures to a minimum, and to address your concern, we have reorganized the figure.

22. Line 241: What do you mean by the phrase ‘18 recipes and 3’? Please modify.

We have made the changes based on your suggestion.

23. Line 247 (Figure 4): In the figure caption, please, rephrase the phrase ‘facilitate childbirth formulations’ for sake of clarity.

We have replaced this term with “uterotonic formulation. (Line 241)

24. Line 264 (Table 3): In the title of the table, replace ‘othe’ with ‘other’.

The title has been changed (Line 258).

25. Line 267 (heading 3.4): Replace ‘survey’ with ‘surveyed’.

The title has been changed as suggested (Line 261).

26. Line 268: Replace ‘relative citation frequency’ with ‘RCF’.

The replacement has been completed (Line 262).

27. Line 274 (heading 3.4): This heading should be put at the beginning of the ‘Results’ section.

The results section can only begin with the overall table, from which all the other aspects highlighted in the text are derived. It is for this reason that we did not begin with the ethnobotanical indices, even though, to a certain extent, they emphasize specific findings.

Reviewer #3

1. Line 103-104: The word invent is not the right choice of word. Authors are not inventing anything here rather they are documenting the traditional knowledge of ................

The sentence was revised as follows: “This study aimed to identify medicinal plants and recipes with uterotonic effects used in traditional medicine in Lubumbashi to facilitate childbirth.”(Lines 97-98)

2. Line 154 : remove google it is not a bibliographic databases.

In response to your request, Google has been removed (Line 156), provided that, for the purposes of this study, all the studies we found on Google were also found on Google Scholar. However, it should be noted that nowadays, there are peer-reviewed journals that are indexed on Google. It would therefore be incomplete not to take a look there to see what is published there (Line 150).

3. Table 1. The first two plant species does not have herbarium voucher specimen number.

The herbarium codes have been added for both plants (Table 1).

4. Table 1. R5, R10 16, R21, and many more are empty.

That’s a good point. However, this situation is perfectly normal. It stems from the fact that these recipes differ from the preceding ones only in the information listed in this column—namely, the part of the plant used and the method of preparation. This is because a single plant can have multiple medicinal uses, particularly when different parts of the plant are used.

Attachment Submitted filename: PONE-D-26-11446-Response to Reviewers.docx

# Editor report: Decision Letter 1

**DOI:** 10.1371/journal.pone.0355373.r003

**Authors:** Ramesh Kumar Ahirwar

21 Jul 2026

Ethnomedicinal Knowledge of plants used in Alternative Medicine as uterotonic in Lubumbashi, Haut-Katanga (DR Congo).

PONE-D-26-11446R1

Dear Dr. Bashige Valentin Chiribagula,

We’re pleased to inform you that your manuscript has been judged scientifically suitable for publication and will be formally accepted for publication once it meets all outstanding technical requirements.

Within one week, you’ll receive an e-mail detailing the required amendments. When these have been addressed, you’ll receive a formal acceptance letter and your manuscript will be scheduled for publication.

An invoice will be generated when your article is formally accepted. Please note, if your institution has a publishing partnership with PLOS and your article meets the relevant criteria, all or part of your publication costs will be covered. Please make sure your user information is up-to-date by logging into Editorial Manager at Editorial Manager® and clicking the ‘Update My Information' link at the top of the page. For questions related to billing, please contact billing support.

If your institution or institutions have a press office, please notify them about your upcoming paper to help maximize its impact. If they’ll be preparing press materials, please inform our press team as soon as possible -- no later than 48 hours after receiving the formal acceptance. Your manuscript will remain under strict press embargo until 2 pm Eastern Time on the date of publication. For more information, please contact onepress@plos.org.

Kind regards,

Ramesh Kumar Ahirwar, Ph.D.

Academic Editor

PLOS One

Additional Editor Comments (optional):

Dear Author,

Congratulations!

We are pleased to inform you that your manuscript has been accepted for publication in its present form. The reviewers' comments have been satisfactorily addressed, and the manuscript meets the journal's publication standards.

Thank you for choosing our journal for the publication of your research. We appreciate your valuable contribution and wish you continued success in your future research endeavors.

Best regards,

Academic Editor

Dr. Ramesh Kumar Ahirwar

Reviewers' comments:

# Editor report: Acceptance letter

**DOI:** 10.1371/journal.pone.0355373.r004

**Authors:** Ramesh Kumar Ahirwar

PONE-D-26-11446R1

PLOS One

Dear Dr. Chiribagula,

I'm pleased to inform you that your manuscript has been deemed suitable for publication in PLOS One. Congratulations! Your manuscript is now being handed over to our production team.

At this stage, our production department will prepare your paper for publication. This includes ensuring the following:

* All references, tables, and figures are properly cited

* All relevant supporting information is included in the manuscript submission,

* There are no issues that prevent the paper from being properly typeset

You will receive further instructions from the production team, including instructions on how to review your proof when it is ready. Please keep in mind that we are working through a large volume of accepted articles, so please give us a few days to review your paper and let you know the next and final steps.

Lastly, if your institution or institutions have a press office, please let them know about your upcoming paper now to help maximize its impact. If they'll be preparing press materials, please inform our press team within the next 48 hours. Your manuscript will remain under strict press embargo until 2 pm Eastern Time on the date of publication. For more information, please contact onepress@plos.org.

You will receive an invoice from PLOS for your publication fee after your manuscript has reached the completed accept phase. If you receive an email requesting payment before acceptance or for any other service, this may be a phishing scheme. Learn how to identify phishing emails and protect your accounts at https://explore.plos.org/phishing.

If we can help with anything else, please email us at customercare@plos.org.

Thank you for submitting your work to PLOS ONE and supporting open access.

Kind regards,

PLOS ONE Editorial Office Staff

on behalf of

Dr. Ramesh Kumar Ahirwar

Academic Editor

PLOS One
